# Global Mapping of Population Exposure to Upstream Gas Flaring Using Integrated VIIRS Nightfire and GHSL Data, 2016–2023, with Projections to 2030

**DOI:** 10.3390/toxics13121053

**Published:** 2025-12-05

**Authors:** Sotiris Zikas, Christos Christakis, Loukas-Moysis Misthos, Ioannis Psomadakis, Angeliki I. Katsafadou, Ioannis Tsilikas, George C. Fthenakis, Vasilis Vasiliou, Yiannis Kiouvrekis

**Affiliations:** 1 Mathematics, Computer Science and Artificial Intelligence Laboratory, Faculty of Public and One Health, University of Thessaly, V. Griva 13, 43100 Karditsa, Greece; szikas@uth.gr (S.Z.); lmmisthos@uniwa.gr (L.-M.M.); psomadak@uth.gr (I.P.); 2 Faculty of Public and One Health, University of Thessaly, V. Griva 13, 43100 Karditsa, Greece; cchristakis@uth.gr (C.C.); agkatsaf@uth.gr (A.I.K.); 3 Department of Surveying & Geoinformatics Engineering, University of West Attica, Egaleo Park Campus, Ag. Spyridonos Str., 12243 Athens, Greece; 4 Department of Environmental Health Sciences, Yale School of Public Health, New Haven, CT 06511, USA; 5 Department Applied Physics and Mathematics, National Technical University of Athens, Iroon Polytechniou 9, Zografou, 15772 Athens, Greece; tsilikasgiannis@hotmail.com; 6 Veterinary Faculty, University of Thessaly, 43100 Karditsa, Greece; 7 Department of Information Technologies, University of Limassol, Limassol 3020, Cyprus; 8 Business School, University of Nicosia, 46 Makedonitissas Avenue, Nicosia 2417, Cyprus

**Keywords:** gasflaring, population exposure, upstream oil and gas, VIIRS nightfire, GHSL, SMOD, urbanization, health risk, spatial analysis, 2030 projections

## Abstract

Gas flaring from upstream oil and gas production remains a significant source of air pollution and toxic emissions, with major implications for human health and climate. However, the number of people living near flaring has not been quantified globally. This study presents the first worldwide, settlement-scale assessment of populations living within 1 km and 3 km of active upstream flare sites between 2016 and 2023, with projections to 2030. Using the VIIRS Nightfire satellite product, which provides global detections of high-temperature combustion sources, and the Global Human Settlement Layer (GHSL) population and settlement data, we developed a transparent and reproducible geospatial workflow to compute proximity-based exposure indicators by buffering flare locations and intersecting them with population rasters The analysis provides consistent estimates across five settlement categories: rural, peri-urban/suburban, semi-dense urban, dense urban, and urban centres. The VIIRS-based flaring time series combined with GHSL projections allows us to estimate how many people are likely to live near upstream flares under current flaring patterns by 2030. Results show that exposure is concentrated in a few oil-producing countries. Nigeria remains the most affected, with over 100,000 urban residents exposed in 2023. India and Pakistan dominate peri-urban and semi-urban exposures, while Indonesia and Iraq persist as multi-settlement hotspots. Although moderate declines are observed in China and Iran, little progress is evident in Nigeria, Mexico, and Indonesia. Projections for 2030 suggest exposure will increase substantially, driven by population growth and urban expansion, with about 2.7 million people living within 1 km and 14.8 million within 3 km of flaring sites. The findings establish the first globally consistent baseline for population exposure to gas flaring, supporting the monitoring and mitigation objectives of the Zero Routine Flaring by 2030 initiative.

## 1. Introduction

Gas flaring refers to the combustion of natural gas released during oil extraction, particularly when adequate infrastructure for its collection or utilization is lacking [1]. Understanding the extent of flaring is crucial not only for assessing its contribution to greenhouse gas emissions but also for evaluating its impacts on ecosystems and public health. Although the environmental consequences—such as air pollution and climate change—are widely recognized, the direct and indirect effects on people living near flaring sites are increasingly a cause for concern. Emissions from flaring significantly degrade air quality and exacerbate climate change [2].

Within the framework of the Paris Agreement (2016), nations committed to reducing their emissions [3]. In alignment with these objectives, the World Bank launched the “Zero Routine Flaring by 2030” initiative, which aims to eliminate the routine burning of approximately 140 bcm of natural gas annually, thereby preventing the release of about 300 million tons of CO_2_ [4]. Achieving this target is of vital importance, as gas flaring not only affects the climate but also has direct health implications for populations residing near oil and gas facilities.

Fawole et al. [5] strongly criticize existing literature, arguing that the exclusion of flaring from emission inventories and global climate models results in an underestimation of pollutants. They propose a conceptual framework for developing more accurate and resilient emission factors for flaring activities.

**Figure 1:**
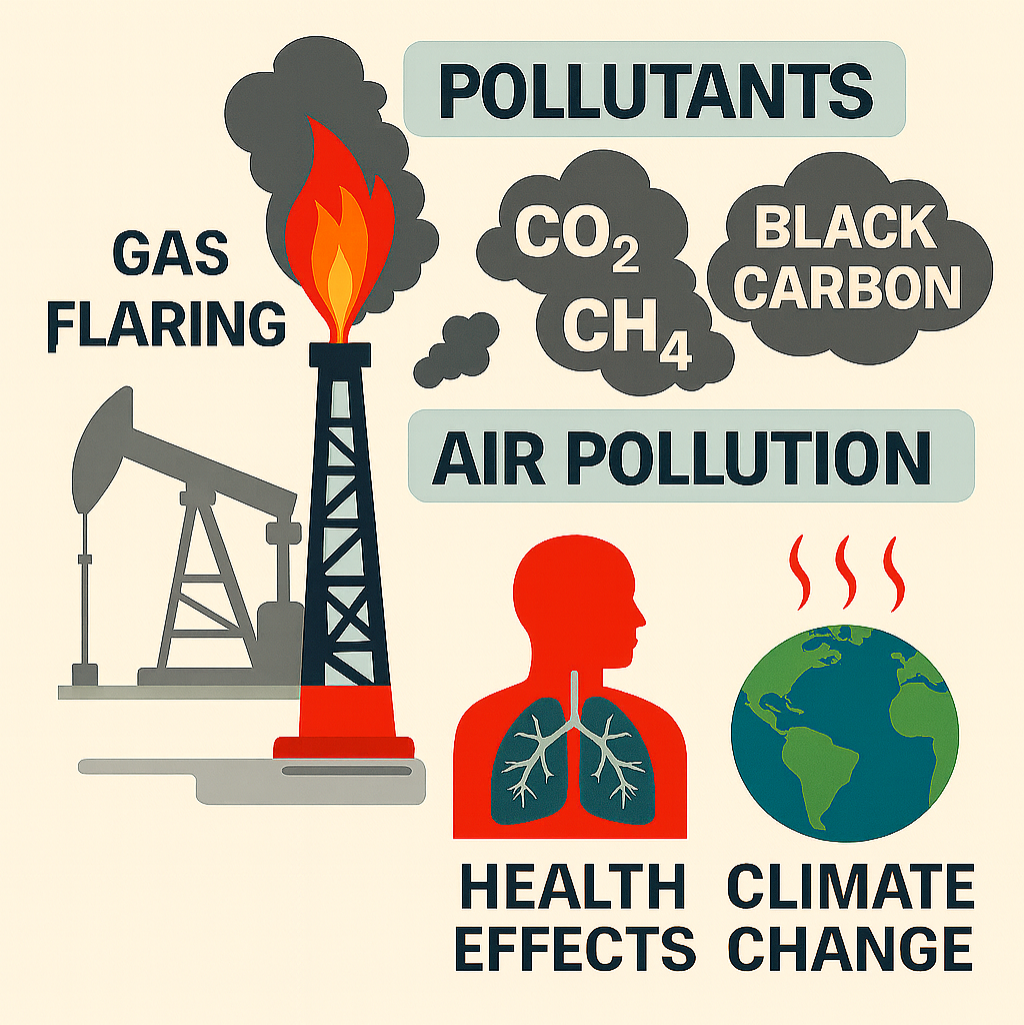
Infographic illustrating the process of gas flaring, highlighting emitted pollutants and their impacts on air pollution, climate change, and human health.

From an environmental and public health perspective (Figure 1), it is important to note that flaring releases not only CO_2_ but also methane (CH_4_), which has a much higher global warming potential—over 80 times greater than CO_2_ over a 20-year horizon. According to World Bank estimates, in 2022, flaring accounted for approximately 357 million tons of CO_2_-equivalent emissions, of which 42 million tons were methane, a quantity comparable to the emissions from about three million vehicles.

With respect to public health, studies conducted across various regions associate flaring with increased respiratory problems, premature deaths, and chronic diseases among residents living near flaring sites [6,7]. The risks are particularly elevated for pregnant women and newborns [8], and higher rates of childhood asthma and other respiratory disorders have been documented [9]. Ajugwo’s study [10] vividly highlights the health impacts of gas flaring in Nigeria.

Janitz et al. [11] investigated health risks associated with natural gas drilling, particularly adverse outcomes for newborns. A comprehensive review of the literature on human exposure and health risks linked to oil extraction is provided by Johnston et al. [12]. From an initial pool of 2236 publications, 63 relevant studies were identified: 22 human, 5 occupational, 5 animal, 6 experimental, and 31 community-based. The review underscores multiple exposure pathways—air, soil, water, and waste—through which communities are affected. Evidence links oil extraction to a range of adverse health outcomes, including cancer, liver dysfunction, immunodeficiency, and neurological disorders. Furthermore, McKenzie et al. [13] examined the relationship between the intensity of oil and gas (O&G) activity and biological indicators of cardiovascular health, finding that higher activity intensity correlates with an increased augmentation index (a measure of arterial stiffness).

Finally, the global and national burden of disease associated with flaring is assessed by Motte et al. [14] using disability-adjusted life years (DALYs). Their evaluation links flaring emissions with mid-point indicators commonly used in life cycle assessment (LCA), such as climate change. The results indicate that, globally, flaring contributes approximately 4.83 × 10^5^ DALYs per year, equivalent to 6.19 × 10^−5^ DALYs per person per year. All of the above highlight the necessity and rationale for the present research.

### Scope and Innovation of the Study

This study quantifies the number of people living in proximity to oil and gas flaring facilities on a global scale. The analysis focuses on flares from upstream production facilities and does not include midstream energy system activities. We use the term exposure in accordance with the definitions established by the United Nations Office for Disaster Risk Reduction (UNDRR) and the United Nations Office for Outer Space Affairs (UN-SPIDER) [15,16]. UNDRR defines exposure as “the situation of people, infrastructure, housing, production capacities and other tangible human assets located in hazard-prone areas”, while UN-SPIDER specifies that measures of exposure may include “the number of people or types of assets in an area”. Following this internationally recognized terminology, we quantify exposure as the number of individuals residing within a defined proximity to gas-flaring sites, which are treated as localized environmental hazards. This spatially based measure reflects the presence of populations in hazard-prone areas and is distinct from hazard-intensity metrics (e.g., volume of gas flared or pollutant concentrations), which relate to the severity or magnitude of potential impacts rather than to exposure itself.

We define proximity indicators as the number of individuals residing within 1 km and 3 km of active flaring sites during the period 2016–2023. To estimate the spatial distribution of the population, we employ the Visible Infrared Imaging Radiometer Suite (VIIRS) Nightfire dataset in combination with GHSL POP R2023A and SMOD Level 2, enabling the assessment of exposure across settlement categories (urban centre, dense-urban, semi-dense urban, suburban/peri-urban, and rural). We use population and settlement information from the Global Human Settlement Layer (GHSL) POP R2023A and the Settlement Model (SMOD) Level 2. GHSL POP R2023A provides global gridded population estimates at 100 m to 1 km resolution for multiple years and projections to 2030, while SMOD Level 2 classifies each grid cell into settlement types (urban centre, dense urban cluster, semi-dense urban cluster, suburban/peri-urban, rural cluster). Together, these datasets allow us to quantify how many people in each settlement class live within defined distances of flaring sites.

The VIIRS is a satellite instrument that measures visible and infrared light to observe Earth’s surface and atmosphere. Its Nightfire data product is the primary global source for detecting and quantifying gas flaring from space [17].

To date, global studies have examined flaring intensity and emitted volumes, while epidemiological and environmental research has typically focused on specific countries or local communities. What has been missing is a global and systematic representation of how many people live near flares from production facilities, using a unified methodology that ensures spatial and temporal comparability.

The present study contributes to the growing body of evidence linking residential proximity to flaring with adverse health effects. Epidemiological research has documented associations between flaring and respiratory morbidity [7], as well as preterm birth and reduced birth weight [8]. Consistent with these observations, reviews and satellite-enabled assessments indicate that flaring contributes to air-pollution mixtures relevant for population health, particularly fine particulate matter and co-pollutants in affected regions [5,18,19].

The innovation of this study lies in several key aspects. First, it represents the first global, settlement-specific quantification of populations residing near upstream flares, applying a consistent methodology across multiple years. Second, it provides a comparable eight-year time series (2016–2023) to monitor trends and assess policy impacts. Third, the indicators are designed for direct use by governmental and international organizations, supporting the prioritization of interventions and identification of exposure hotspots. Fourth, the methodology is transparent and reproducible, featuring a straightforward workflow that can be re-executed as VIIRS or population data are updated. Fifth, the analysis highlights spatial exposure patterns across settlement types, showing where populations live near to flares are most concentrated and how these patterns evolve over time. Sixth, the analytical scope is explicitly aligned with the domain where commitments to eliminate routine flaring apply (upstream production). Finally, the resulting dataset can be directly linked with health, socioeconomic, or environmental data in future studies, without the need to redefine exposure metrics.

It should be noted that the study does not simulate atmospheric pollutant dispersion nor directly estimate health outcomes; rather, the results describe potential exposure, not disease burden.

## 2. Materials and Methods

### 2.1. The Dataset, Data Collection, and Data Preparation

The dataset [20] contains site-level estimates of annual gas flaring volumes for 102 countries from 2012 to 2023 (see Table 1). The data set comprises 145,642 site-year records representing approximately 90,230 unique flare locations, identified by country and geographic coordinates. Over the full period, the total estimated flared volume is 1721.75 billion cubic meters (bcm). Flaring is predominantly onshore (80.24%), with offshore flaring contributing 19.76%. The dataset supports analyses of temporal trends, geographic patterns, and comparisons across asset types (OIL/GAS/LNG) and flare magnitude classes.

Administrative boundary data (country polygons) were retrieved from the Natural Earth Geoportal [21], while urbanization degree and population density data in raster format were sourced from the Global Human Settlement Layer (GHSL) maintained by the European Commission [22]. The GHSL provides consistent, global-scale spatial data on where people live and how settlements evolve, making it essential for studies linking population exposure to environmental or industrial risks [22].

All spatial datasets were projected into a common coordinate reference system using the Mollweide projection, and any spatial inconsistencies in the country shapefile were corrected to ensure accuracy in subsequent analyses.

The total number of flaring sites per country was calculated and stored within the attribute table of the country shapefile. Geographic centroids were also computed for each country to facilitate cartographic visualization using proportional red symbols, whose size reflects the number of flare sites. To estimate the population potentially exposed to emissions, buffer zones of 1 and 3 km were generated around each flaring site. Previous epidemiological and environmental studies have shown that health risks associated with flaring and other combustion-related emissions decrease substantially with increasing distance, with notable reductions beyond 3–5 km [19] from the source. For instance, traffic-related pollutants such as PM2.5 and NO_2_ decline sharply at distances greater than 3 km from major roads [23], while studies of gas flaring report elevated risks of adverse birth outcomes within approximately 5 km [24]. Based on this evidence, a maximum buffer of 5 km could have been considered. However, in densely populated urban areas, 5 km circles frequently overlap, leading to excessive double-counting and potential misrepresentation of exposure. To balance epidemiological relevance with spatial accuracy, we therefore adopted buffer distances of 1 km and 3 km. These thresholds capture the populations most at risk of direct exposure while minimizing the artificial inflation of exposed counts caused by overlapping buffers in high-density settlement zones. Overlapping buffers were dissolved to prevent double-counting of affected populations. The resulting buffer zones were intersected with national boundaries, and zonal statistics were computed on the population raster to estimate the total population residing within these areas. The calculated population data were then joined with the country shapefile and visualized with choropleth maps to represent exposure levels. We therefore selected 1 km to represent immediate toxicological exposure and 3 km to capture near-immediate gradients of health risk. Furthermore, to obtain a deeper understanding of average population exposure per flaring site, the total affected population in each country was divided by the number of active flaring sites in that country. These normalized values were subsequently linked to the respective country shapefiles and visualized for comparative analysis. The use of distance-based proxies does not account for meteorological variation, combustion efficiency, flare-stack design, or emission intensity. However, these proxies are widely applied in environmental health research and provide a reproducible, scalable basis for global exposure estimation. The VIIRS Nightfire dataset may underdetect low-temperature or intermittent flares, particularly in regions with persistent cloud cover or low radiative efficiency; nonetheless, it remains the most validated and comprehensive dataset currently available for global flaring surveillance [25,26].

#### 2.1.1. Global Distribution of Gas Flaring Sites (2023)

**Figure 2:**
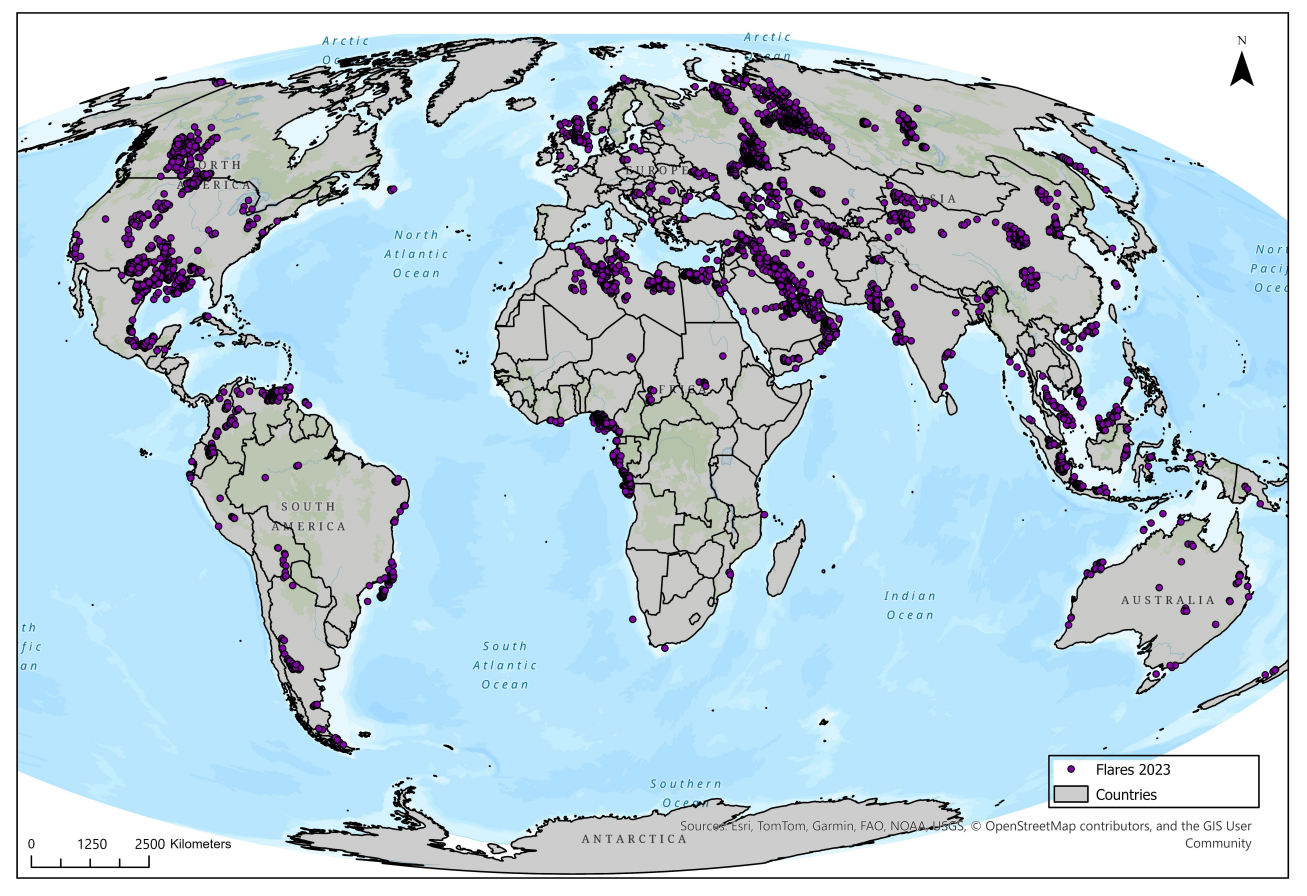
The distribution of flaring points per country.

Figure 2 presents the global distribution of gas flaring points recorded in 2023. Each point represents a confirmed flaring site. High concentrations of flaring activity are visible in Russia and the Caspian Region, as well as in the Middle East, including Iran, Iraq, and Saudi Arabia. In North America, the United States shows widespread flaring, with notable clusters in Texas and coastal regions. In West and Central Africa, dense flare clusters are observed in Nigeria and parts of Angola. South America shows significant flaring in Venezuela, Colombia, Brazil, and Argentina. Europe exhibits moderate flare concentrations in Eastern Europe and the North Sea basin. Asia shows scattered flaring across China, India, and Southeast Asia, with visible activity in offshore areas. Australia displays dispersed flaring sites, while Canada shows flare activity in oil sands and shale regions. Smaller, isolated flare sites are present in New Zealand, Papua New Guinea, and several island nations. Western and Central Europe show minimal flaring activity. No flaring points are recorded in Antarctica and Greenland.

#### 2.1.2. National-Level Distribution of Gas Flaring Sites (2023)

**Figure 3:**
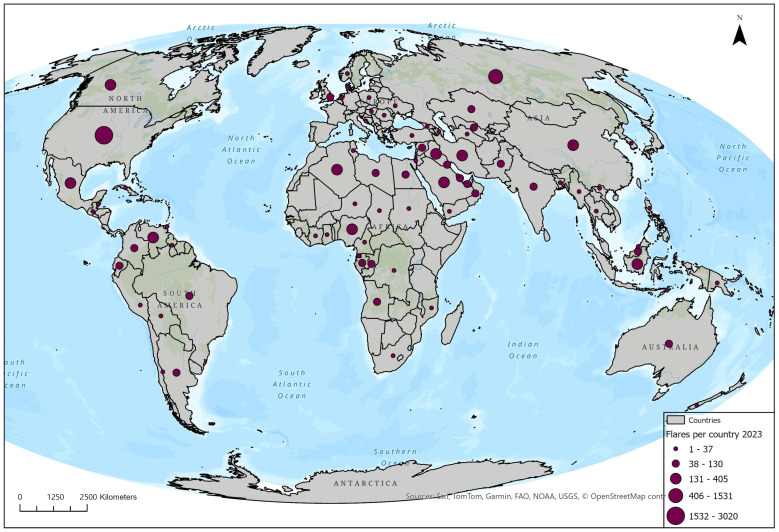
The volume of flaring points per country.

Figure 3 presents the number of recorded gas flaring sites per country for the year 2023, using graduated symbols to represent flare counts. While the number of flaring sites indicates spatial proliferation, it does not directly translate into the magnitude of flaring, as the volume of gas burned varies by site. For this reason, the analysis is complemented by Figure 3, which reports flaring volumes at the country level, providing a weighted representation of overall flaring intensity. This national-scale visualization categorizes countries into five classes based on flare activity, highlighting the spatial concentration and relative burden of flaring practices worldwide. A small group of countries (a) emerges with exceptionally high flare counts, ranging between 1532 and 3020 sites. This includes Russia, which continues to lead globally in total flare numbers due to its extensive upstream oil and gas infrastructure. The United States also ranks high in this category, with flaring concentrated in shale-rich regions such as the Permian Basin and the Bakken Formation, where infrastructure constraints and surges in unconventional extraction have contributed to persistent flare activity. Iran and Iraq are similarly prominent, with routine flaring across widespread oil-producing regions in the absence of sufficient gas-recovery systems. Nigeria completes this high-burden group, with extensive flaring centered in the Niger Delta, reflecting both infrastructural deficits and regulatory challenges. A second group of countries (b) falls within the intermediate range of 406 to 1531 flares. These include China, Libya, Indonesia, and Venezuela, where flaring reflects both growing demand for domestic energy. Algeria and Angola also belong to this group, representing major hydrocarbon producers in Africa with ongoing difficulties in implementing effective flare mitigation strategies. Brazil and India round out this category, driven by increasing oil and gas activities, including offshore developments. In the third and fourth categories (c), which include countries with 38 to 405 flare sites, we observe a mix of mature and emerging oil producers, including Egypt, Mexico, Kazakhstan, Argentina, and several countries in Southeast Asia. Flaring in these cases tends to be more localized and may be seasonal or operationally dependent, often corresponding to specific basins or infrastructure types. The last group (d) consists of countries with minimal flare activity, ranging from 1 to 37 sites. These are widely distributed and include several European nations, parts of Southern Africa, Central America, and smaller island states. Low flare counts in these countries often correspond to limited oil extraction activity, stronger regulatory enforcement, or the successful implementation of gas recovery and utilization systems.

#### 2.1.3. Analysis of Total Volume per Total Flares (2016–2023)

**Figure 4:**
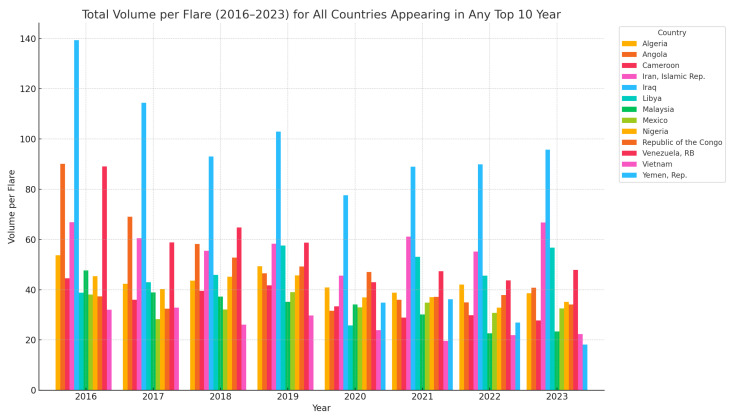
Total volume per flare (2016–2023) for the top-10 countries.

Figure 4 is showing all countries that appeared in the top 10 at least once from 2016 to 2023. This gives a complete picture of how each significant country has changed over time. Based on data from 2016 to 2023 on Total Volume per Total Flares, several clear trends and patterns emerge across countries.

Iraq stands out unmistakably as the leading country every single year, starting with an extremely high volume of 139.3 bcm in 2016 and maintaining values above 89 bcm in most years, with a slight dip in 2020 to 77.6 bcm. This consistent dominance suggests either particularly high flare volumes, lower flare counts, or a combination of both, possibly due to structural characteristics of its oil and gas infrastructure. Iran (Islamic Republic) appears regularly in the top 4, with values ranging from 45.6 bcm in 2020 to a peak of 66.8 bcm in 2016 and again in 2023 at 66.7 bcm. Its stable presence in the upper tier reflects sustained high-volume flaring with some year-to-year variation. Venezuela remains in the top 5 throughout the period, despite a general downward trend from 89.1 bcm in 2016 to 43.7 bcm in 2022. In 2023, a slight rebound to 47.8 bcm is observed, which may suggest operational recovery or renewed flaring activity. Angola, initially at 90.1 bcm in 2016, has shown a steady decline, reaching 40.8 bcm in 2023. This pattern might indicate improvements in flare efficiency or reductions in production volumes. Libya has been consistently observed since 2017, with increasing flare volume, peaking at 56.8 bcm in 2023. This upward trend may reflect expanded operations or worsening flare control. Republic of the Congo enters the top rankings in 2018 and maintains moderate values thereafter 52.8 bcm in 2018; 34–37 bcm in later years, highlighting a growing role in global flare activity. Nigeria, Algeria, and Cameroon appear frequently in the mid-tier range 30–45 bcm, showing moderate but relatively stable flare volumes over time. Malaysia and Mexico generally rank in the lower half of the top 10 list. Malaysia bcm drops out after 2020, while Mexico bcm consistently shows lower values, at 30–32 bcm, indicating relatively lower flare intensity.

### 2.2. Methodology: Urbanization Classification Using SMOD

The study adopts the Settlement Model (SMOD) layers [27], which implement the Degree of Urbanisation Stage I methodology as recommended by the United Nations Statistical Commission. The classification was applied to the global population grid produced by the Joint Research Centre (JRC) for the years 1975 to 2030 in 5-year intervals.

The SMOD layers are determined by integrating built-up surface data extracted from Landsat and Sentinel-2 imagery (GHS-BUILT-S R2023A) with gridded population data from the CIESIN GPW v4.11 dataset (GHS-POP R2023A). The version used in this analysis (SMOD R2023A v2) reflects updated definitions, including refinements to the classification of Semi-Dense Urban Clusters.

We utilized the SMOD at Level 2 (L2) detail, enabling precise classification of settlement patterns. Specifically, five urbanization classes were analyzed:

- 30: Urban Centre Grid Cell
- 23: Dense Urban Cluster Grid Cell
- 22: Semi-Dense Urban Cluster Grid Cell
- 21: Suburban or Peri-Urban Grid Cell
- 13: Rural Cluster Grid Cell

All volumetric quantities of oil and gas are expressed in billion cubic meters (bcm). Although the term “bcm” is most commonly used for natural gas, expressing liquid petroleum volumes in cubic meters facilitates direct comparison on a metric basis.

These classes provide a hierarchical structure to assess spatial and temporal variations in population exposure and urban development. Aggregation to a broader classification (L1) is also supported when needed. The study employed a combination of Python 3.12 geospatial libraries and professional GIS software to conduct spatial analysis and visualization. Core Python tools included Pandas and NumPy for data handling, GeoPandas and Shapely for vector geometry operations, Rasterio for reading and processing raster datasets, Pyproj for coordinate system transformations, Contextily for basemap integration, and Folium for interactive mapping. In addition to these libraries, the analysis was supported by QGIS [28], an open-source desktop GIS platform, for visual inspection, spatial data validation, and map layout design. ArcGIS [29], ESRI [30] were also used for advanced geoprocessing and high-precision cartographic outputs. This integrated toolset enabled robust geospatial workflows throughout the study.

We do not report margins of error or confidence intervals because our results are not based on predictions or statistical sampling. Instead, they are derived through a deterministic geospatial computation: overlaying high-resolution population rasters with buffer zones around flaring sites and directly aggregating the counts. The outcome is therefore a precise calculation of exposed populations given the input datasets, rather than an estimate with statistical uncertainty. Any potential inaccuracies arise from the underlying data sources (e.g., VIIRS flare detection, population distribution in GHSL), not from stochastic variation in our workflow.

Data for 2016 and 2017 were processed using the GHSL 2015 dataset, whereas data for all subsequent years were derived from the 2020 dataset to reflect the closest available temporal match. The most recent calculations were performed using flaring data from 2023, together with GHSL population projections for 2030. The GHSL population datasets are available at spatial resolutions of 100 m and 1000 m. For population exposure estimations, the 100 m dataset was primarily utilized. However, for calculations involving SMOD, a compatibility issue required using the 1000 × 1000 m population dataset to ensure alignment with the SMOD data structure.

## 3. Results

It is important to note that the countries shown in the heatmaps and the trend chart are not the top 12, 15, or 17 countries for each individual year, but rather the set of countries that appear among the top 5 or top 10 highest-impact cases at least once during the period 2016–2023. Some countries exhibit high population exposure within 1 km of flaring sites only in specific years, while in other years they fall below the upper ranks. For this reason, the final list includes countries that appear repeatedly over the eight-year window, even if they do not consistently rank in the top positions each year. This also explains why some heatmaps display 12, others 15, and others 17 countries instead of a fixed number, such as the top 10, since several countries reach notable exposure values intermittently rather than continuously. This approach ensures that the analysis captures all countries that ever reach a high exposure value during the study period, rather than limiting the selection to those that consistently rank highly every year. The heatmaps illustrate this clearly: several countries exhibit pronounced exposure peaks only in particular years, confirming that the selected set is based on overall prominence across the full period rather than on strict year-by-year rankings.

### 3.1. Population Within 1 km of Flares Elements by Country (2016–2023)

**Figure 5:**
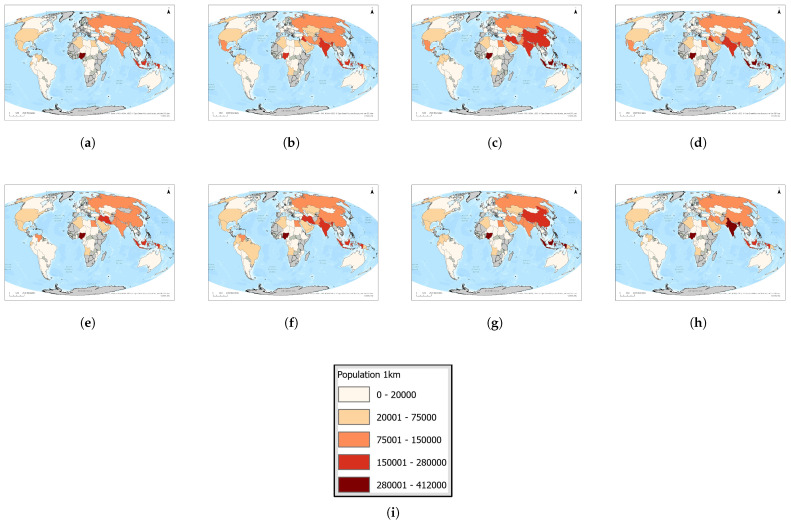
Global maps of the maximum number of people residing within 1 km of active upstream flaring sites, 2016–2023. Panels: (**a**) 2023, (**b**) 2022, (**c**) 2021, (**d**) 2020, (**e**) 2019, (**f**) 2018, (**g**) 2017, (**h**) 2016, and (**i**) the legend. For full-resolution images and detailed visual analysis, please refer to the Supplementary Materials (Figures S1–S8).

**Figure 6:**
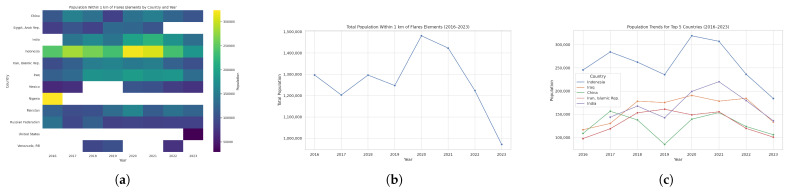
(**a**) Heatmap of population near flares by country and year. (**b**) Total population within 1 km of flaring elements, 2016–2023. (**c**) Countries that ever ranked in the top 5 by population living within 1 km of flaring sites during 2016–2023. For full-resolution images and detailed visual analysis, please refer to the Supplementary Materials (Figures S9–S11).

In Figure 5, we present a time series (2016–2023) of each country’s maximum population residing in a single 1 km^2^ grid cell. Figure 6a highlights the twelve countries that consistently rank among the global top ten over this period, revealing a remarkably stable group of high-density locations.

Figure 6b,c summarizes the total number of people living within 1 km of flaring sites and identifies the five most affected countries for each year. In 2016, roughly 1,296,816 people lived near flares, with Nigeria, Indonesia, Russia, Pakistan, and Iraq most affected. The following year, the total declined to 1,202,945, led by Indonesia, China, India, Iraq, and Iran. In 2018, the total number rose again to 1,296,488, driven by Indonesia, Iraq, India, Iran, and China, before falling to 1,247,252 in 2019 (Indonesia, Iraq, Iran, India, Pakistan). In 2020, population exposure peaked at 1,480,563—primarily in Indonesia, India, Iraq, Pakistan, and Iran—then decreased to 1,423,045 in 2021 (Indonesia, India, Iraq, Iran, China) and 1,223,337 in 2022 (Indonesia, Iraq, India, Pakistan, China). By 2023, the total reached its lowest point at 971,618, with Indonesia, India, Iraq, Pakistan, and China remaining the most affected. Throughout 2016–2023, Indonesia, India, Iraq, Pakistan, China, and Iran consistently appear among the five countries with the largest populations near flaring sites.

### 3.2. Population Within 3 km of Flares Elements by Country (2016–2023)

**Figure 7:**
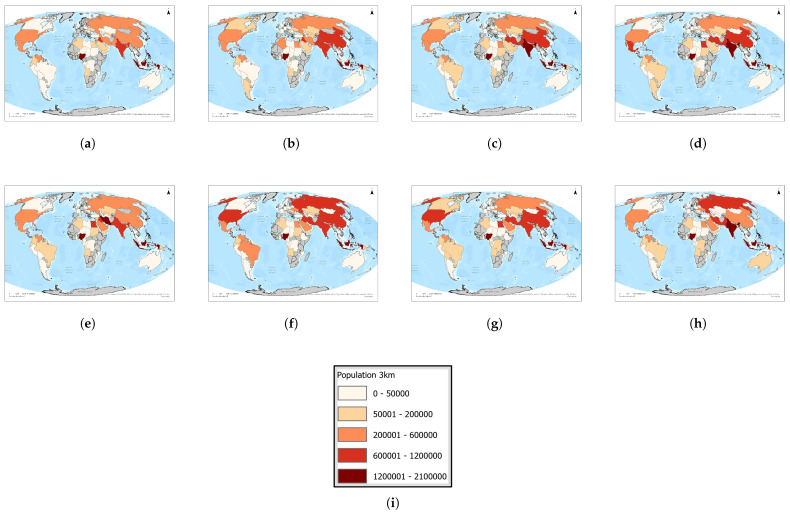
Global maps showing, for each country, the maximum number of people residing within 3 km of active upstream flaring sites for the years (**a**) 2023, (**b**) 2022, (**c**) 2021, (**d**) 2020, (**e**) 2019, (**f**) 2018, (**g**) 2017, and (**h**) 2016. (**i**) The legend. For full-resolution images and detailed visual analysis, please refer to the Supplementary Materials (Figures S12–S19).

**Figure 8:**
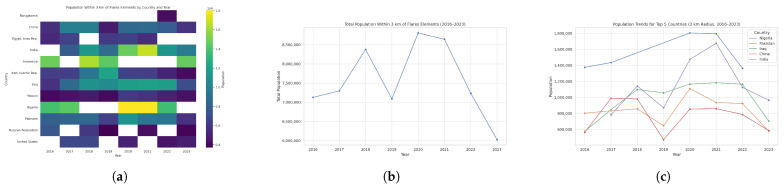
(**a**) Heatmap of population near flares by country and year (3 km radius). (**b**) Total population within 3 km of flaring elements for the period 2016–2023. (**c**) Countries that ever ranked in the top 5 by population living within 3 km of flaring sites during 2016–2023. For full-resolution images and detailed visual analysis, please refer to the Supplementary Materials (Figures S20–S22).

In Figure 7, we show a time series (2016–2023) of each country’s maximum population within a radius of 3 km. Figure 8a highlights the twelve countries that appear in the annual top ten over this period, revealing a remarkably stable group of high-density locations.

Figure 8b,c summarizes the total number of people living within 3 km of flaring sites and identifies the five most affected countries each year. In 2016, approximately 7,127,174 people lived within 3 km of flares across nine countries, with Indonesia (1,479,452), Nigeria (1,375,098), Pakistan (803,243), the Russian Federation (760,320), and Iran (632,853) most exposed; in 2017, the total rose to 7,297,205, led by Nigeria (1,434,636), China (989,203), Iraq (847,668), Pakistan (833,324), and India (783,149); in 2018 it climbed to 8,378,305, driven by Indonesia (1,628,811), India (1,141,450), Iraq (1,098,070), China (979,707), and Iran (927,997); 2019 saw a decline to 7,088,776, with Indonesia (1,445,555), Iran (1,200,590), Iraq (1,056,994), India (871,525), and Pakistan (647,885) most affected; in 2020 exposure peaked at 8,808,104—primarily in Nigeria (1,803,833), India (1,473,674), Iraq (1,165,139), Pakistan (1,110,260), and China (853,826); 2021 recorded 8,643,855 people near flares, notably in Nigeria (1,794,524), India (1,676,036), Iraq (1,183,083), Pakistan (936,520), and China (859,549); in 2022 the total fell to 7,235,113, led by Nigeria (1,364,012), Iraq (1,165,038), India (1,125,643), Pakistan (924,009), and China (786,599); and by 2023 it reached its lowest point at 6,026,231, with Indonesia (1,465,760), India (965,446), Iraq (705,791), China (586,830), and Pakistan (576,843) remaining the most exposed.

### 3.3. Population near Urban Centre Areas by Country: 2016–2023

This report analyzes the population living in urban centres within 1 km of gas flaring sites in countries that enter the top 10 by exposed population at least once during the period 2016–2023. These data provide insights into population exposure to flaring in densely populated urban hubs.

**Figure 9:**
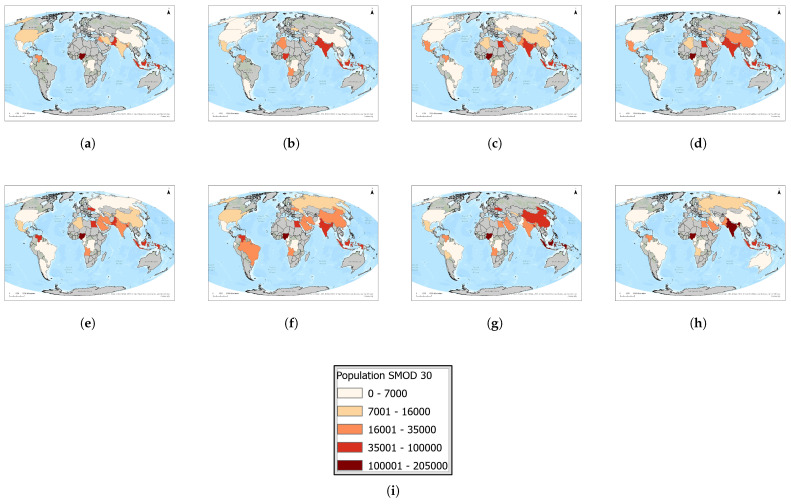
Global maps showing, for each country, the maximum number of people residing in urban centre areas within a 1 km radius of active flaring sites for the years (**a**) 2023, (**b**) 2022, (**c**) 2021, (**d**) 2020, (**e**) 2019, (**f**) 2018, (**g**) 2017, (**h**) 2016. (**i**) The legend. For full-resolution images and detailed visual analysis, please refer to the Supplementary Materials (Figures S23–S30).

**Figure 10:**
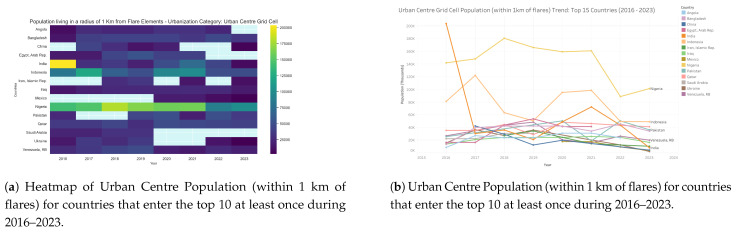
Urban centre population living within 1 km of active flaring sites for countries that enter the top 10 at least once during 2016–2023: (**a**) Heatmap visualization showing temporal and geographic patterns. (**b**) Comparative totals by country and year. For full-resolution images and detailed visual analysis, please refer to the Supplementary Materials (Figures S31 and S32).

The largest absolute exposures to flaring occur in dense urban centres (Figure 9 and Figure 10), where very large populations are concentrated. Nigeria is the dominant case globally, beginning with approximately 140,000 exposed individuals in 2016, peaking near 180,000 in 2018, and remaining above 100,000 in 2023. India showed extremely high exposure in 2016, exceeding 200,000, but this dropped sharply to around 20,000 by 2019, partially recovered to 70,000 in 2021, and fell again to fewer than 10,000 by 2023. This pattern suggests either rapid regulatory change or inconsistencies in the detection of the satellite record. Indonesia maintained a consistently high exposure, ranging between 60,000 and 100,000, with 50,000 still affected in 2023. Pakistan also showed a steady increase, peaking at 50,000 in 2020 before modest declines thereafter. Other countries, including Venezuela, Mexico, Iraq, and Bangladesh, recorded moderate exposures in the 20,000–50,000 range. By contrast, exposures in China, Iran, Egypt, and the Gulf States remained relatively low. These findings highlight that urban centres in Nigeria, Indonesia, and Pakistan are the most persistent global hotspots of human exposure to gas flaring, whereas India’s dramatic reduction warrants further investigation. Urban centre exposures are of particular concern because they affect the largest absolute populations, compounding risks in environments already burdened by air pollution and high population density.

### 3.4. Population near Dense Urban Areas by Country: 2016–2023

This report focuses on the population living in dense urban areas within 1 km of gas flaring sites in countries that enter the top 10 by exposed population at least once during the period 2016–2023. The data reflect population exposure to flaring in highly populated urban zones.

**Figure 11:**
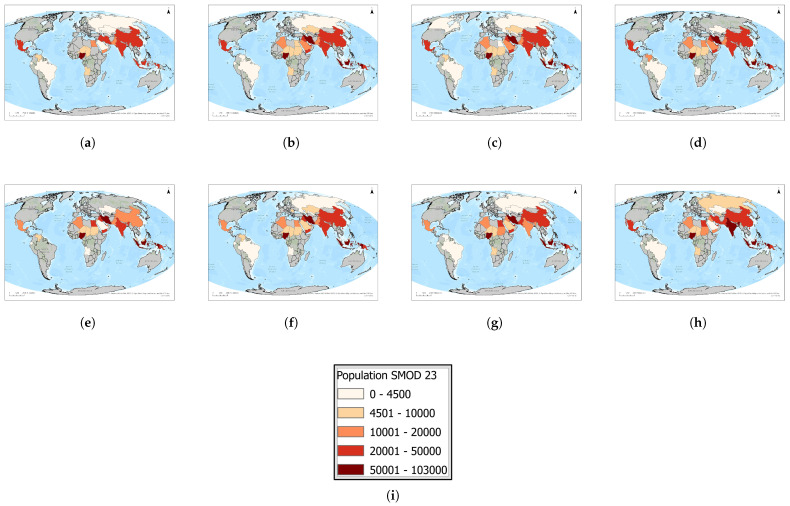
Global maps showing, for each country, the maximum number of people residing in dense urban areas within a 1 km radius of active flaring sites for the years (**a**) 2023, (**b**) 2022, (**c**) 2021, (**d**) 2020, (**e**) 2019, (**f**) 2018, (**g**) 2017, (**h**) 2016. (**i**) The legend. For full-resolution images and detailed visual analysis, please refer to the Supplementary Materials (Figures S33–S40).

**Figure 12:**
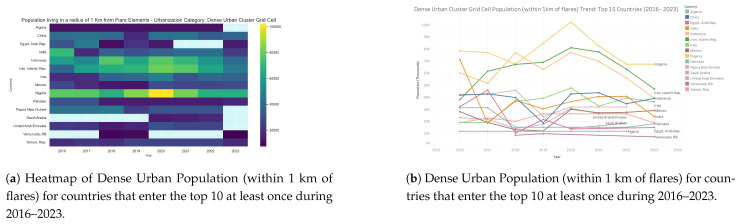
Dense urban population living within 1 km of active flaring sites for countries that enter the top 10 at least once during 2016–2023: (**a**) Heatmap visualization showing temporal and geographic patterns. (**b**) Comparative totals by country and year. For full-resolution images and detailed visual analysis, please refer to the Supplementary Materials (Figures S41 and S42).

Dense urban clusters represent the largest single concentrations of populations living near gas flaring (Figure 11 and Figure 12). Nigeria stands out as the most affected country, with consistently high exposure levels averaging around 70,000–80,000 individuals annually and peaking at more than 100,000 in 2020. Iran and Indonesia form the second tier of hotspots: both countries recorded steady increases to peaks of approximately 80,000 and 77,000, respectively, in 2020 before declining to 47,000–56,000 by 2023. Iraq also exhibited significant exposure, rising to nearly 47,000 in 2020 before falling to 32,000 by 2023. China and India, while among the world’s largest flare producers, registered more moderate dense urban exposures of 30,000–40,000, reflecting different spatial overlaps between flaring sites and dense urban settlements. Mexico remained stable in the 25,000–30,000 range throughout the period, while other producer states, such as Saudi Arabia, the United Arab Emirates, Algeria, and Venezuela, recorded comparatively low exposures (<20,000). Importantly, 2020 marked the global peak in dense urban exposure, likely reflecting both production dynamics and pandemic-related disruptions. These findings highlight that dense urban flare exposure is concentrated in a handful of producer states, with Nigeria, Iran, Indonesia, and Iraq emerging as critical global hotspots where mitigation would deliver the greatest benefits to human health.

### 3.5. Population near Semi-Dense Urban Areas by Country: 2016–2023

**Figure 13:**
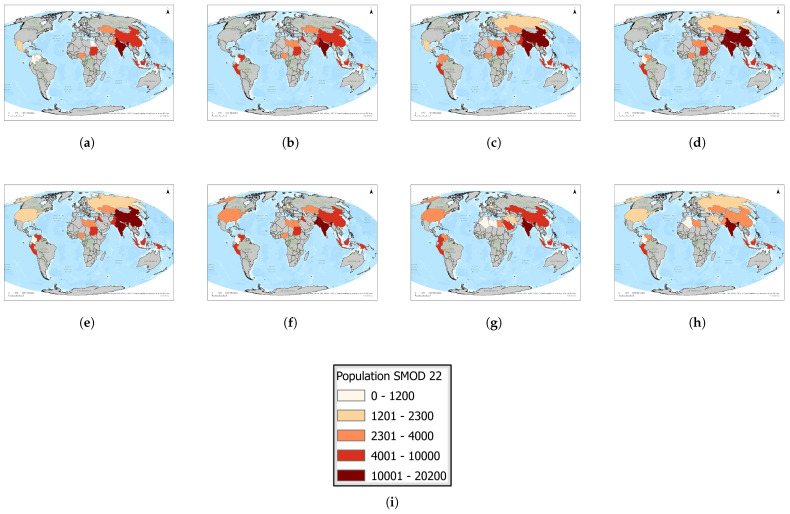
Global maps showing, for each country, the maximum number of people residing in semi-dense urban areas within a 1 km radius of active flaring sites for the years (**a**) 2023, (**b**) 2022, (**c**) 2021, (**d**) 2020, (**e**) 2019, (**f**) 2018, (**g**) 2017, (**h**) 2016. (**i**) The legend. For full-resolution images and detailed visual analysis, please refer to the Supplementary Materials (Figures S43–S50).

**Figure 14:**
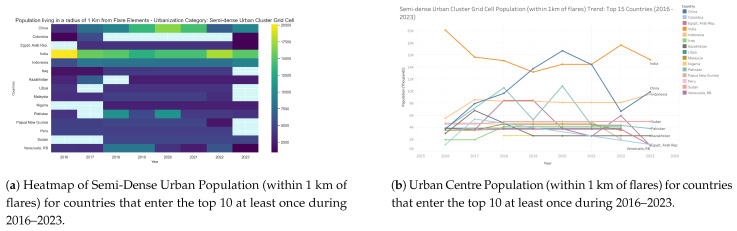
Semi-dense urban population living within 1 km of active flaring sites for countries that enter the top 10 at least once during 2016–2023: (**a**) Heatmap visualization showing temporal and geographic trends. (**b**) Comparative totals by country and year. For full-resolution images and detailed visual analysis, please refer to the Supplementary Materials (Figures S51 and S52).

Semi-dense urban settlements exposed to flaring within 1 km (Figure 13 and Figure 14) represent a smaller but highly vulnerable category of affected populations. India consistently accounts for the largest exposures, beginning at approximately 20,000 individuals in 2016, declining to about 13,000 by 2019, then rising again to nearly 18,000 in 2022, before a slight reduction in 2023. China and Indonesia form the second tier, with China reaching 16,000–17,000 exposed individuals by 2020 but dropping sharply to 6000 in 2022 before recovering to around 10,000 in 2023, while Indonesia maintained a steady upward trajectory to nearly 10,000. Pakistan and Venezuela experienced temporary peaks above 10,000 around 2018–2020 but later declined, whereas Sudan consistently showed smaller but non-negligible exposures of around 5000–8000. Other countries, such as Iraq, Kazakhstan, Libya, Malaysia, and Peru, remained below 5000 throughout the study period. These results illustrate that, although absolute population numbers in semi-dense urban clusters are lower than in rural or peri-urban contexts, the relative risk is higher due to population density and existing environmental burdens. Persistent hotspots in India and fluctuating but significant exposures in China underscore the importance of targeted flare-mitigation measures in semi-urban areas where vulnerable populations may be disproportionately affected.

### 3.6. Population near Suburban Areas by Country: 2016–2023

**Figure 15:**
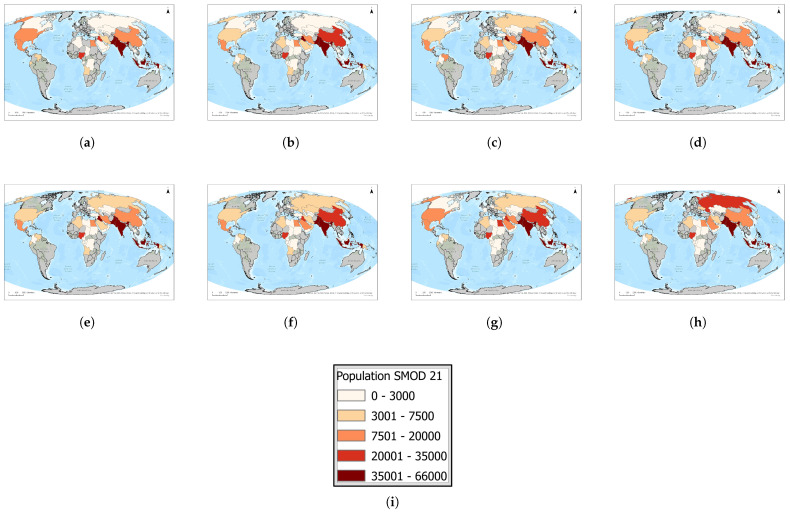
Global maps showing, for each country, the maximum number of people residing in suburban/peri-urban areas within a 1 km radius of active flaring sites for the years (**a**) 2023, (**b**) 2022, (**c**) 2021, (**d**) 2020, (**e**) 2019, (**f**) 2018, (**g**) 2017, (**h**) 2016. (**i**) The legend. For full-resolution images and detailed visual analysis, please refer to the Supplementary Materials (Figures S53–S60).

**Figure 16:**
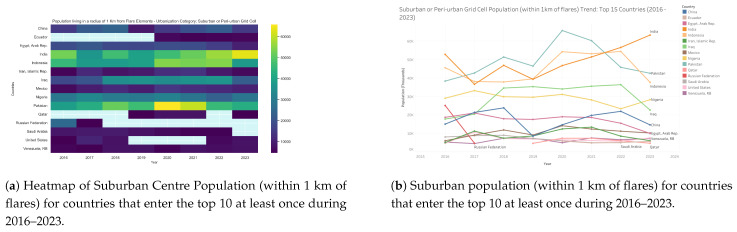
Suburban population living within 1 km of active flaring sites for countries that enter the top 10 at least once during 2016–2023: (**a**) Heatmap showing temporal and geographic distribution. (**b**) Comparative totals by country and year. For full-resolution images and detailed visual analysis, please refer to the Supplementary Materials (Figures S61 and S62).

Suburban populations within 1 km of flares (Figure 15 and Figure 16) are concentrated primarily in South and Southeast Asia. India and Pakistan emerge as the most affected countries, together accounting for the majority of global exposure in this settlement category. India exhibits a consistent upward trend, rising from approximately 45,000 exposed individuals in 2016 to more than 62,000 in 2023, making it the single largest suburban exposure hotspot. Pakistan reached a peak of nearly 65,000 in 2020, then declined somewhat in subsequent years, but still recorded more than 45,000 exposed individuals in 2023. Indonesia represents the third-highest case, maintaining a steady upward trend to nearly 40,000 in 2023. Other producer states, including Nigeria, Iraq, and Iran, sustained medium levels of suburban exposure ranging from 25,000 to 35,000 over the study period, with little evidence of decline. By contrast, several countries, most notably China, Russia, and Saudi Arabia, recorded much lower suburban exposure (<15,000 by 2023), in some cases reflecting downward trends. Taken together, these results highlight that suburban and peri-urban communities in India and Pakistan are currently the most affected globally by gas flaring activity, while persistent medium exposure in Nigeria, Iraq, and Indonesia underscores a continued lack of effective mitigation in key producing regions.

### 3.7. Population near Rural Areas by Country: 2016–2023

**Figure 17:**
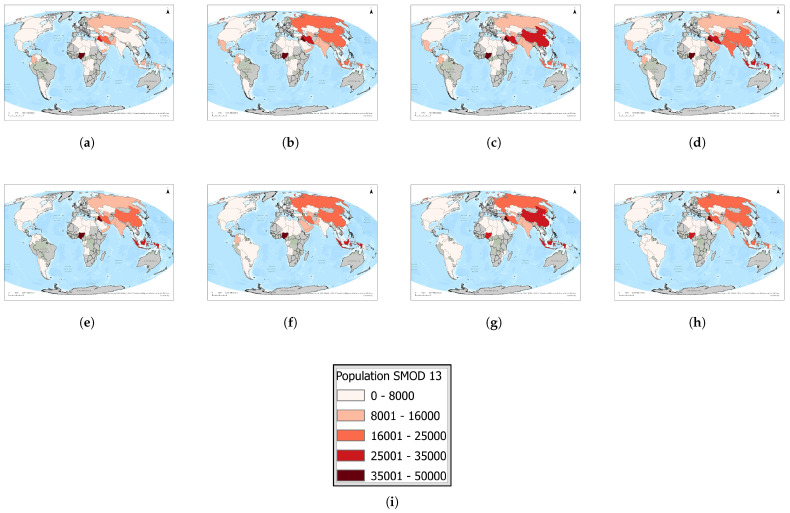
Global maps showing, for each country, the maximum number of people residing in **rural areas** within a 1 km radius of active flaring sites for the years (**a**) 2023, (**b**) 2022, (**c**) 2021, (**d**) 2020, (**e**) 2019, (**f**) 2018, (**g**) 2017, (**h**) 2016. (**i**) The legend. For full-resolution images and detailed visual analysis, please refer to the Supplementary Materials (Figures S63–S70).

**Figure 18:**
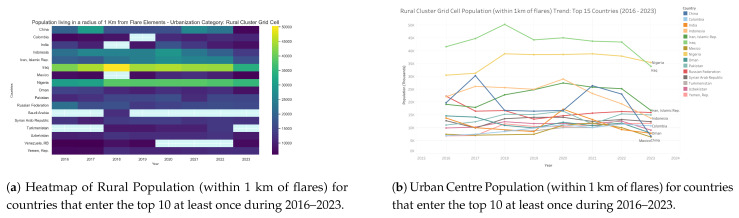
Rural population living within 1 km of active flaring sites for countries that enter the top 10 at least once during 2016–2023: (**a**) Heatmap visualization showing temporal and geographic patterns. (**b**) Comparative totals by country and year. For full-resolution images and detailed visual analysis, please refer to the Supplementary Materials (Figures S71 and S72).

Analysis of rural cluster populations (Figure 17 and Figure 18) living within 1 km of flares between 2016 and 2023 reveals persistent and uneven exposure across the top flaring countries. Iraq, Nigeria, and Mexico consistently record the largest exposed populations, with Iraq peaking at more than 50,000 people in 2018 before declining to approximately 35,000 by 2023. Nigeria maintained consistently high exposure levels of around 38,000–40,000 throughout most of the study period, with only a modest decline in the final years. Mexico similarly shows little improvement, with rural populations exposed to flares remaining stable at approximately 38,000–39,000 across the full period. By contrast, China and India exhibit sharp declines: China peaked at nearly 30,000 exposed individuals in 2017 but fell to under 10,000 by 2023, while India followed a similar downward trajectory after 2020. Medium-exposure countries such as Iran, Indonesia, and Colombia maintained levels between 10,000 and 20,000, with only gradual reductions. Lower-exposure countries, including the Russian Federation, Syria, Oman, and Pakistan, remained relatively stable near 10,000–15,000. These results highlight that while some countries appear to benefit from stricter regulation or energy transitions, others, particularly Nigeria and Mexico, have seen little progress, leaving large rural populations persistently exposed to flaring-related emissions.

### 3.8. Predictive Modeling of Human Exposure to Gas Flaring in 2030

This section presents predicted estimates of the population expected to be exposed to gas flaring activities by the year 2030, based on flaring activity recorded in 2023, combined with GHSL population projections for 2030 (Table 2). These projections provide a forward-looking perspective, offering critical insights into how demographic trends and urban expansion will influence the spatial distribution of at-risk populations in the coming years. The analysis is designed to support planning and policy interventions to mitigate the health and environmental impacts of gas flaring, in alignment with global objectives, such as the Zero Routine Flaring by 2030 initiative.

#### 3.8.1. Overall Population Exposure

The results indicate a substantial projected increase in the number of people residing near flaring sites by 2030. Within a 1 km radius, the total affected population is predicted to reach approximately 2.70 million individuals (2,700,770.64). Expanding the radius to 3 km dramatically increases the projected exposure to 14.85 million individuals (14,853,912.41). This represents a more than fivefold increase when the buffer is extended from 1 km to 3 km, reflecting the far-reaching potential of flaring emissions and the population growth expected in areas surrounding oil and gas production sites. These projections suggest that without significant mitigation measures, millions of people will remain at heightened risk from direct and indirect impacts of flaring.

#### 3.8.2. Projected Distribution by Settlement Type (SMOD Classification)

To better understand the dynamics of population growth and exposure, the analysis incorporates Settlement Model (SMOD) Level 2 classifications, which categorize human settlements into five distinct classes: Rural Clusters (SMOD 13), Suburban/Peri-Urban Areas (SMOD 21), Semi-Dense Urban Clusters (SMOD 22), Dense Urban Clusters (SMOD 23), and Urban Centres (SMOD 30). This classification enables the identification of spatial and demographic trends that will shape future exposure scenarios.

#### 3.8.3. 1 km Proximity—Immediate Impact Zone

Within the 1 km buffer, the projected exposure patterns for 2030 reveal a significant concentration of populations in urban and densely developed areas. Dense Urban Clusters (SMOD 23) and Urban Centres (SMOD 30) each account for approximately 637,406 and 631,131 exposed individuals, respectively, representing nearly half of the total population at this distance. Suburban/Peri-Urban Areas (SMOD 21) are projected to have 439,954 exposed individuals, underscoring the role of expanding metropolitan peripheries in shaping future risk. Rural Clusters (SMOD 13) are expected to include 374,329 affected individuals, highlighting persistent risks for populations in less developed regions. Semi-Dense Urban Clusters (SMOD 22) have the lowest predicted exposure, with 104,087 individuals, indicating that transitional settlement zones remain relatively less impacted compared to urban cores and peri-urban fringes. These findings suggest that by 2030, urban populations will bear the greatest direct exposure, exacerbating existing health and environmental stressors in cities.

#### 3.8.4. 3 km Proximity—Broader Influence Zone

When the analysis is extended to a 3 km radius, the population at risk increases dramatically, particularly within high-density urban areas. Urban Centres (SMOD 30) dominate the projections, with an estimated 6.32 million people exposed, representing 42.6% of the total population within this buffer. Dense Urban Clusters (SMOD 23) are projected to reach 2.82 million exposed individuals, highlighting the continued vulnerability of secondary urban zones. Suburban/Peri-Urban Areas (SMOD 21) show a sharp increase to 2.54 million individuals, reflecting rapid outward urban sprawl and the increasing overlap between residential zones and industrial activities. Rural Clusters (SMOD 13) will encompass approximately 963,257 exposed individuals, while Semi-Dense Urban Clusters (SMOD 22) are projected to reach 456,676, remaining the smallest group overall.

### 3.9. Synthesis of Main Exposure Patterns

The analysis of potential population exposure to upstream gas flaring between 2016 and 2023 reveals a three-fold pattern critical for mitigation efforts. Firstly, exposure is highly concentrated geographically, with a small group of large oil producers, namely India, Indonesia, Iraq, Nigeria, Pakistan, and China, consistently accounting for the majority of people living within 1 km and 3 km of flaring sites. Secondly, the structure of exposure is strongly dependent on settlement type: at the closest proximity (1 km), urban centers host a large fraction of exposed individuals, while a wider radius (3 km) shows the majority of exposed populations residing in a mix of urban, peri-urban, and rural clusters, reflecting how settlement growth has encroached upon the spatial extent of flaring. Finally, temporal trends are heterogeneous: While countries such as China and Iran show gradual declines in exposed populations, others, such as Nigeria, Mexico, and Indonesia, exhibit persistent or only weakly declining exposure, despite international commitments to reduce flaring. These findings collectively underscore that (i) mitigation can be effectively prioritized in a select number of high-exposure countries, and (ii) without immediate, targeted intervention, the ongoing processes of urbanization and demographic growth are projected to increase the total number of people living near flaring sites by 2030.

## 4. Discussion

This study presents the first globally harmonized assessment of populations residing near active upstream gas-flaring sites, with consistent spatial and temporal coverage from 2016 to 2023 and projections to 2030. Findings indicate that potential population exposure to flaring is highly concentrated, with six countries, India, Indonesia, Iraq, Nigeria, Pakistan, and China, accounting for over 60% of the global annual exposure. A notable trend is the increasing spatial overlap between active flaring locations and urban or peri-urban areas, suggesting that ongoing urban expansion is intersecting with oil and gas infrastructure, thereby raising concerns about population-level exposure to flaring emissions. These patterns align with the literature, indicating that proximity-based metrics can capture meaningful gradients in potential exposure around upstream oil and gas activity.

Proximity bands of 1 km and 3 km were used as exposure proxies, following methodologies used in prior studies reporting health associations within similar ranges [19].

Emissions commonly associated with gas flaring, such as nitrogen oxides (NO_*x*_), sulphur dioxide (SO_2_), volatile organic compounds (VOCs, including benzene), fine particulate matter (PM2.5), and black carbon, are known to adversely affect respiratory, cardiovascular, and developmental health [5,18]. Analysis of global exposure patterns revealed that more than 40% of individuals living within 1 km of flaring sites reside in urban or dense urban settlements. In many of these settings, access to healthcare and environmental monitoring infrastructure remains limited. Prior studies have highlighted that health impacts from upstream oil and gas activity tend to disproportionately affect socioeconomically marginalised groups [7]. The intersection of high population density with flaring activity underscores the need to frame gas flaring as a public health issue in addition to its environmental and climate dimensions.

Beyond human health, gas flaring may also pose risks to animal and ecosystem health, in accordance with a One Health perspective. Studies have linked flaring exposure to reproductive and immunological effects in livestock near oil and gas sites [31,32], and biomonitoring using animal health indicators has proven effective in contaminated rural settings [33]. Such cross-species impacts highlight the relevance of integrated monitoring approaches in which human and animal populations share exposure pathways. The high-resolution, globally consistent exposure estimates developed in this study offer an opportunity to support public health surveillance, environmental justice analyses, and air quality planning. These data can be integrated into epidemiological studies, particularly when linked with atmospheric dispersion modelling or health registry data. As recommended by prior reviews, combining settlement-resolved proximity with clinical and registry outcomes can improve exposure–response estimation and clarify vulnerable subgroups [12]. Implications for mitigation follow directly from the emissions profile and technological literature.

Enclosed or otherwise improved flare systems, stronger capture and utilisation of associated gas, and operational controls have been shown to reduce pollutant releases and safety risks [2]. By identifying where flaring intersects with large population centres, the present results can help prioritise deployments of these technologies to maximise health co-benefits. Interpretation of these findings should consider methodological constraints inherent to large-scale geospatial analyses. Although pollutant concentrations and health outcomes were not directly measured, the settlement-level population exposure estimates presented here fill a critical data gap and offer a robust foundation for environmental health research, surveillance planning, and risk assessment. The present study complements these findings by identifying where such exposures are most widespread and persistent, thereby offering a practical basis for evaluating mitigation progress and potential health co-benefits.

The projections for 2030 reveal several critical insights into the future dynamics of populations residing near flaring sites. By 2030, more than 60% of the exposed population within 3 km will reside in urban centres or dense urban clusters, illustrating how urban expansion and population growth are intensifying flaring-related risks. These trends are consistent with broader global urbanization forecasts, suggesting that regulatory and technological interventions must prioritize urban contexts to protect the largest absolute populations. The sharp increase in peri-urban exposure, from 439,954 individuals at 1 km to 2.54 million at 3 km, underscores the challenge of managing industrial activities on the urban fringe. Without targeted land-use planning and stricter zoning regulations, these areas may become critical hotspots for human-flaring interactions. Although rural populations constitute a smaller proportion of the total population, their projected exposure remains significant, particularly given limited access to healthcare and environmental monitoring infrastructure. Nearly one million rural residents are expected to live within 3 km of active flaring sites by 2030, indicating that mitigation strategies must extend beyond urban areas to ensure equity.

These projections highlight the urgent need for strategic interventions as the world approaches 2030. Without substantial progress toward the Zero Routine Flaring by 2030 target, millions of individuals will continue to face elevated health risks, especially in urban and peri-urban zones. Policy measures should focus on urban hotspot mitigation, where absolute exposure levels will be highest, by strengthening monitoring and regulation in rapidly growing peri-urban regions and providing targeted support for rural populations to address their heightened vulnerability and resource constraints.

## 5. Conclusions

This study introduces the first globally consistent dataset that quantifies population proximity to upstream gas flaring, broken down by settlement type, spanning 2016–2023 and incorporating projections for 2030. The resulting annually comparable, proximity-based estimates provide a powerful and scalable technique for identifying hotspots, guiding public health surveillance, and informing mitigation strategies in regions where flaring intersects with dense human settlements. By offering a reproducible and transparent spatial framework, the study supports integration into environmental health research, policy planning, and climate action. This work also lays the groundwork for linking proximity quality modelling and epidemiological analysis, and future efforts may further enhance its impact through integrated approaches such as One Health, particularly in shared human–animal environments. The 2030 projections demonstrate a widening spatial and demographic footprint of flaring-related risks, driven by urban growth and continued industrial activity. If current trends persist, more than 14 million people worldwide will live within 3 km of flaring sites, with a substantial proportion residing in urban areas. These findings reinforce the importance of integrating predictive population models with geospatial flaring data to guide evidence-based policies and interventions, ensuring that mitigation efforts are effectively targeted to protect the most vulnerable populations. These findings offer an actionable framework for toxicological risk assessment, enabling policymakers, regulators, and health practitioners to prioritize hotspots for monitoring and intervention.

## Figures and Tables

**Table 1:** Description of variables included in the flared gas dataset, including variable names, data types, measurement units, and brief explanations of their meanings.

Name	Type	Units	Description
Country	categorical	—	Country/territory name
Latitude, longitude	float	degrees (WGS84)	Site coordinates
Year	int	year	Calendar year of estimate
bcm	float	billion cubic meters	Annual flared volume at site-year
MMscfd	float	million scf per day	Average daily flaring rate equivalent
Flaring vol. (million m^3^)	float	million m^3^	Annual flared volume (redundant with bcm × 1000)
Field type	categorical	—	Asset type: OIL, GAS, LNG (plus UNKNOWN)
Location	categorical	—	ONHORE/OFFSHORE classification
Flare level	categorical	—	Magnitude class: Small, Medium, Large

**Table 2:** Projected population exposure to gas flaring in 2030 by proximity and settlement type (SMOD classification).

Category	Population (People)
1 km Total	2,700,770.64
3 km Total	14,853,912.41
1 km SMOD 13 (Rural Clusters)	374,329.23
1 km SMOD 21 (Suburban/Peri-Urban Areas)	439,954.46
1 km SMOD 22 (Semi-Dense Urban Clusters)	104,086.70
1 km SMOD 23 (Dense Urban Clusters)	637,406.32
1 km SMOD 30 (Urban Centres)	631,131.47
3 km SMOD 13 (Rural Clusters)	963,256.93
3 km SMOD 21 (Suburban/Peri-Urban Areas)	2,539,878.61
3 km SMOD 22 (Semi-Dense Urban Clusters)	456,676.39
3 km SMOD 23 (Dense Urban Clusters)	2,819,635.08
3 km SMOD 30 (Urban Centres)	6,323,391.71

## Data Availability

The original contributions presented in this study are included in the article/Supplementary Material. Further inquiries can be directed to the corresponding author.

## Supplementary Materials

The following supporting information can be downloaded at: https://www.mdpi.com/article/10.3390/toxics13121053/s1.

## Abbreviations

The following abbreviations are used in this manuscript:

CATF	Clean Air Task Force
GHSL	Global Human Settlement Layer
MCSAI	Machine Learning and Computer Vision for Spatial Analysis and Intelligence Lab
SMOD	Settlement Model (Level 2)
VIIRS	Visible Infrared Imaging Radiometer Suite
ZRF	Zero Routine Flaring
CO_2_	Carbon Dioxide
CH_4_	Methane
NO_*x*_	Nitrogen Oxides
SO_2_	Sulfur Dioxide
VOC	Volatile Organic Compounds
BCM	Billion Cubic Meters
