# Supplementary material for: Global Mapping of Population Exposure to Upstream Gas Flaring Using Integrated VIIRS Nightfire and GHSL Data, 2016–2023, with Projections to 2030
Source: Toxics. 2025 Dec 5;13(12):1053. doi: 10.3390/toxics13121053 (PMC12737142; doi:10.3390/toxics13121053)
Supplement: Supplementary file 1 [file toxics-13-01053-s001.zip › Supplementary Figures S1-S51.pdf]

Supplementary Materials for

Global Mapping of Population Exposure to Upstream Gas Flaring Using Integrated VIIRS Nightfire and GHSL Data, 2016–2023, with Projections to 2030

This PDF includes:

Supplementary Figures S1–S51

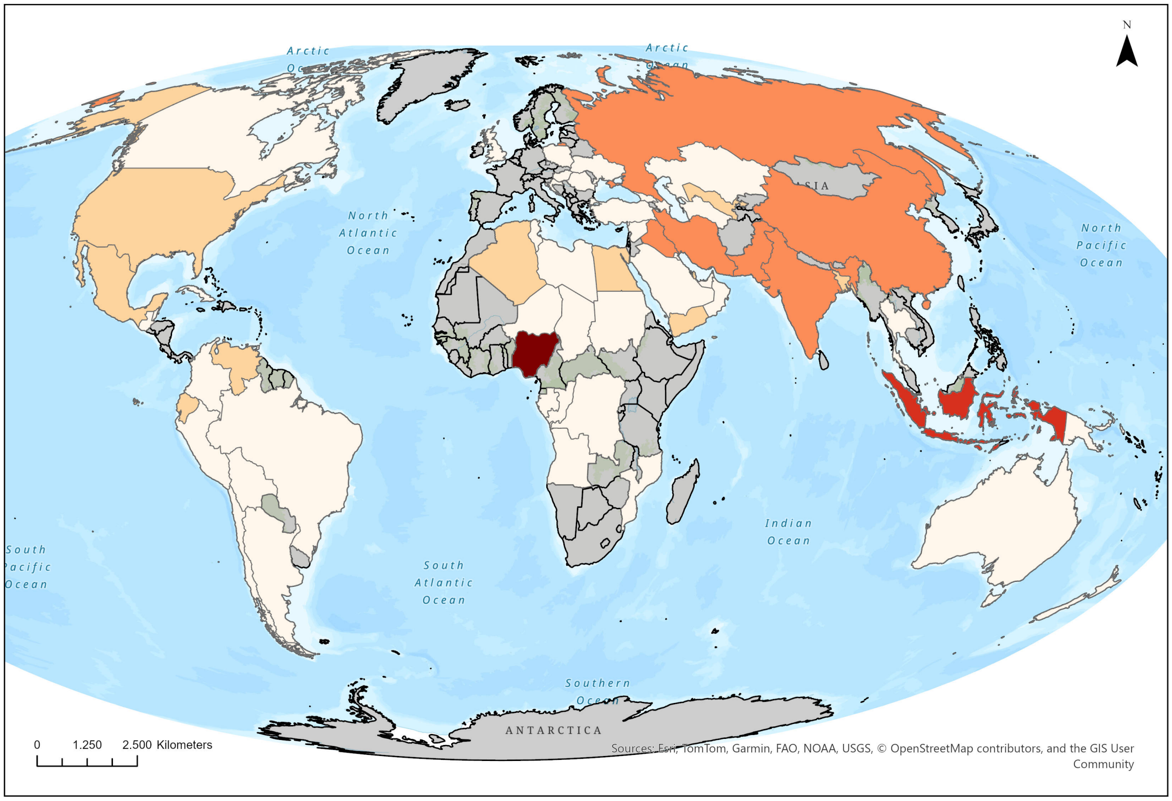

Figure S1: Global map of the maximum number of people residing within 1 km of active upstream flaring site for the year 2023

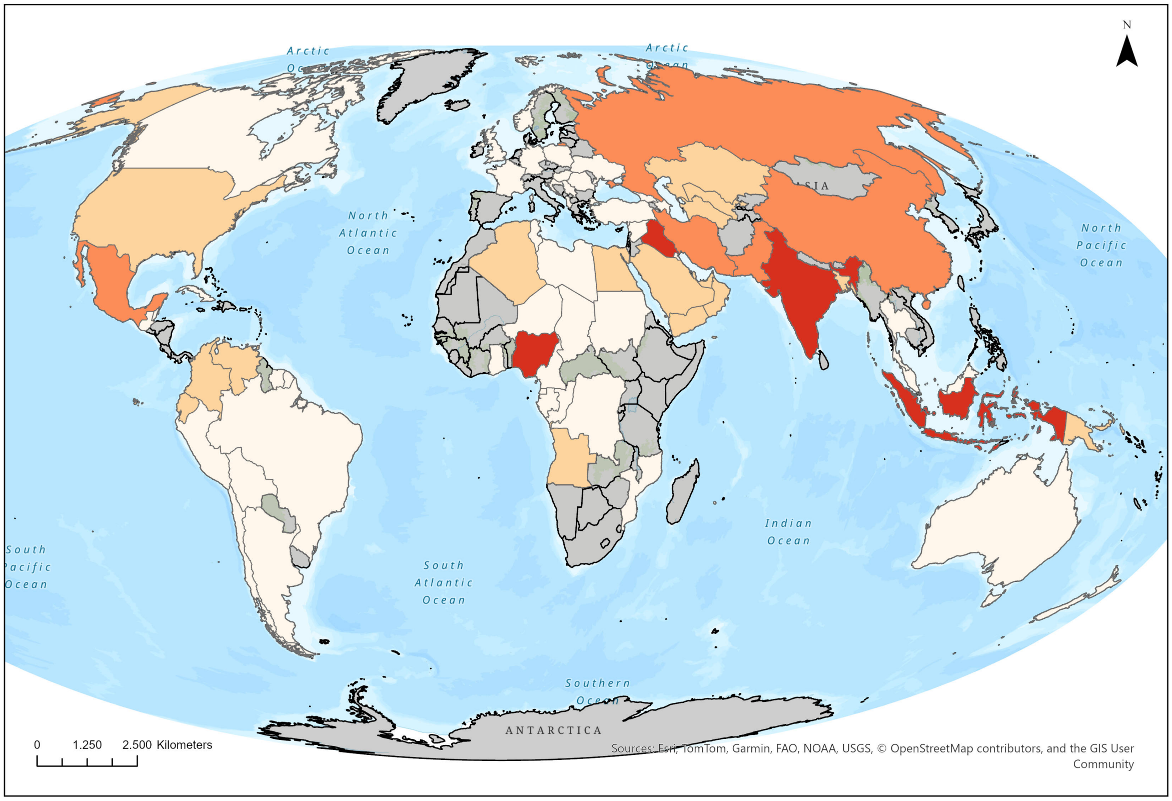

Figure S2: Global map of the maximum number of people residing within 1 km of active upstream flaring site for the year 2022

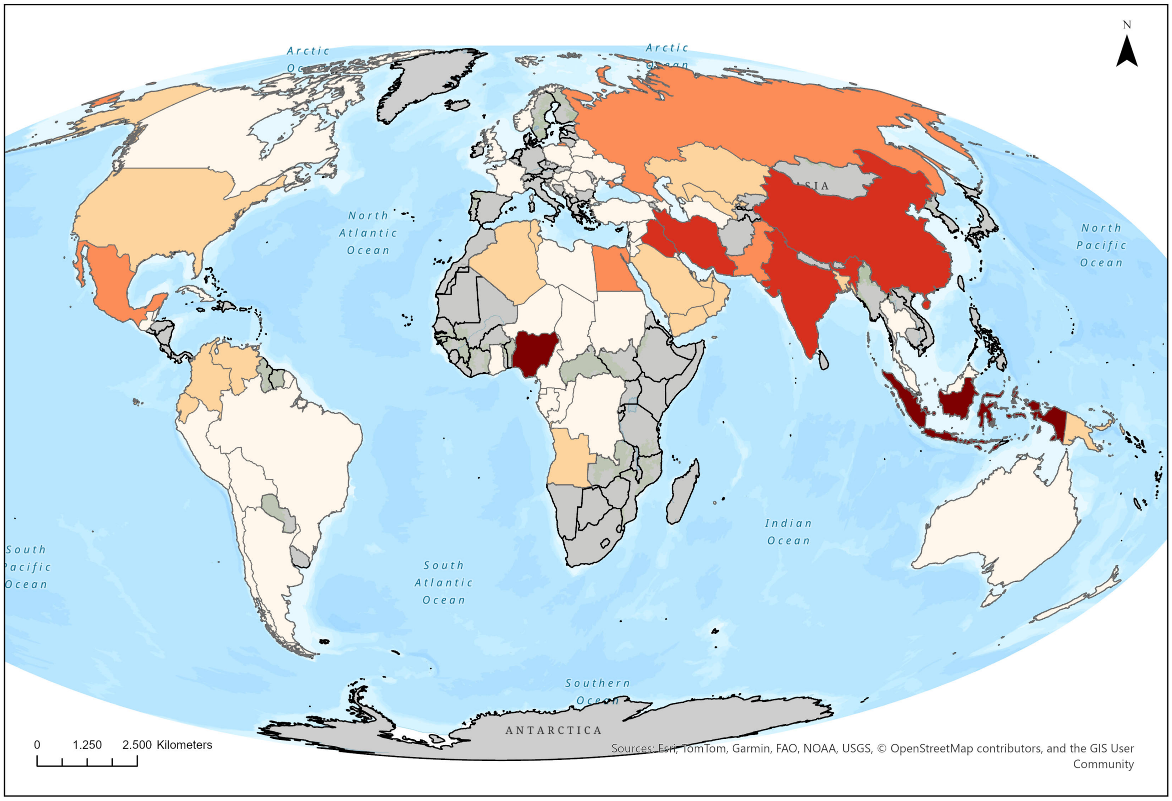

Figure S3: Global map of the maximum number of people residing within 1 km of active upstream flaring site for the year 2021

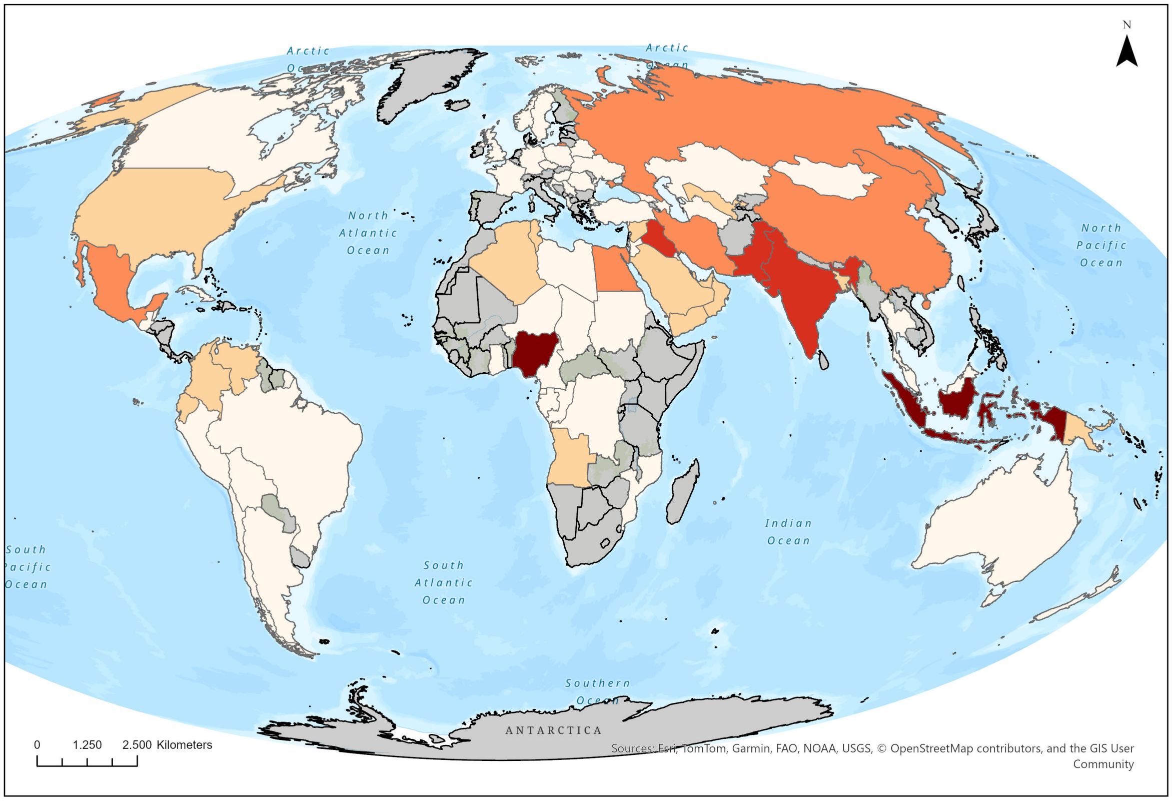

Figure S4: Global map of the maximum number of people residing within 1 km of active upstream flaring site for the year 2020

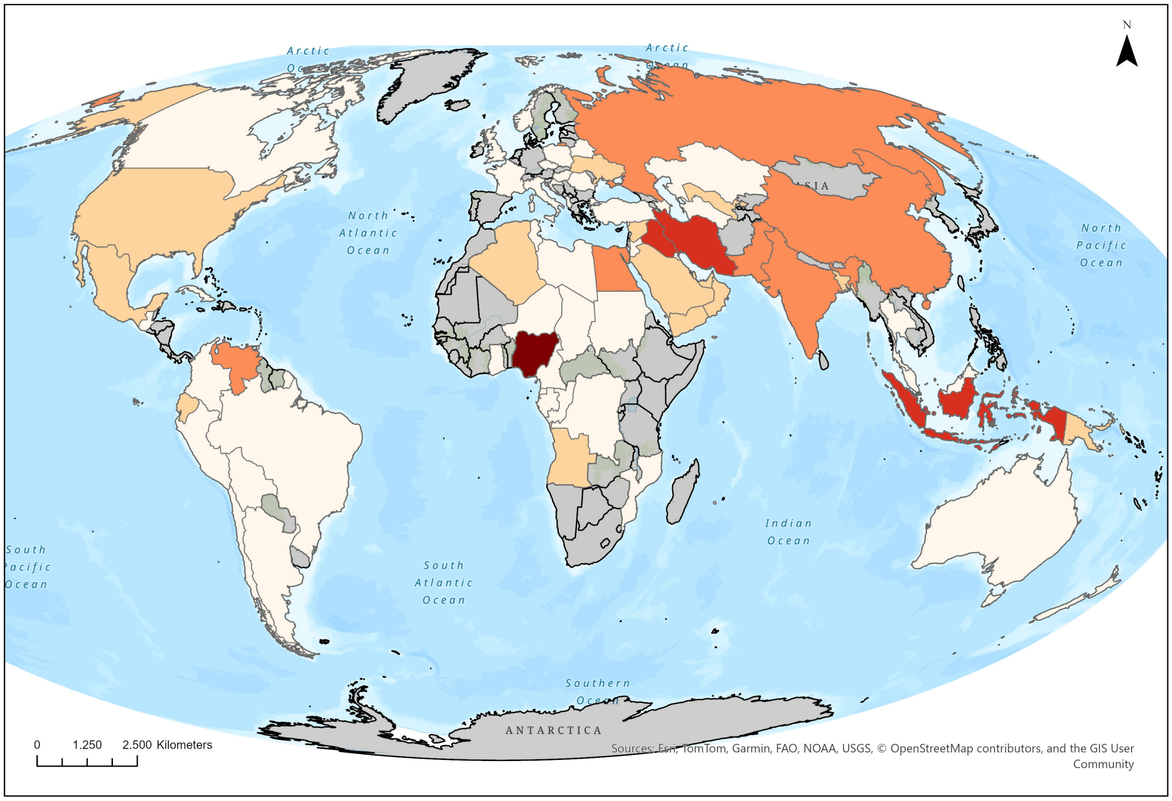

Figure S5: Global map of the maximum number of people residing within 1 km of active upstream flaring site for the year 2019

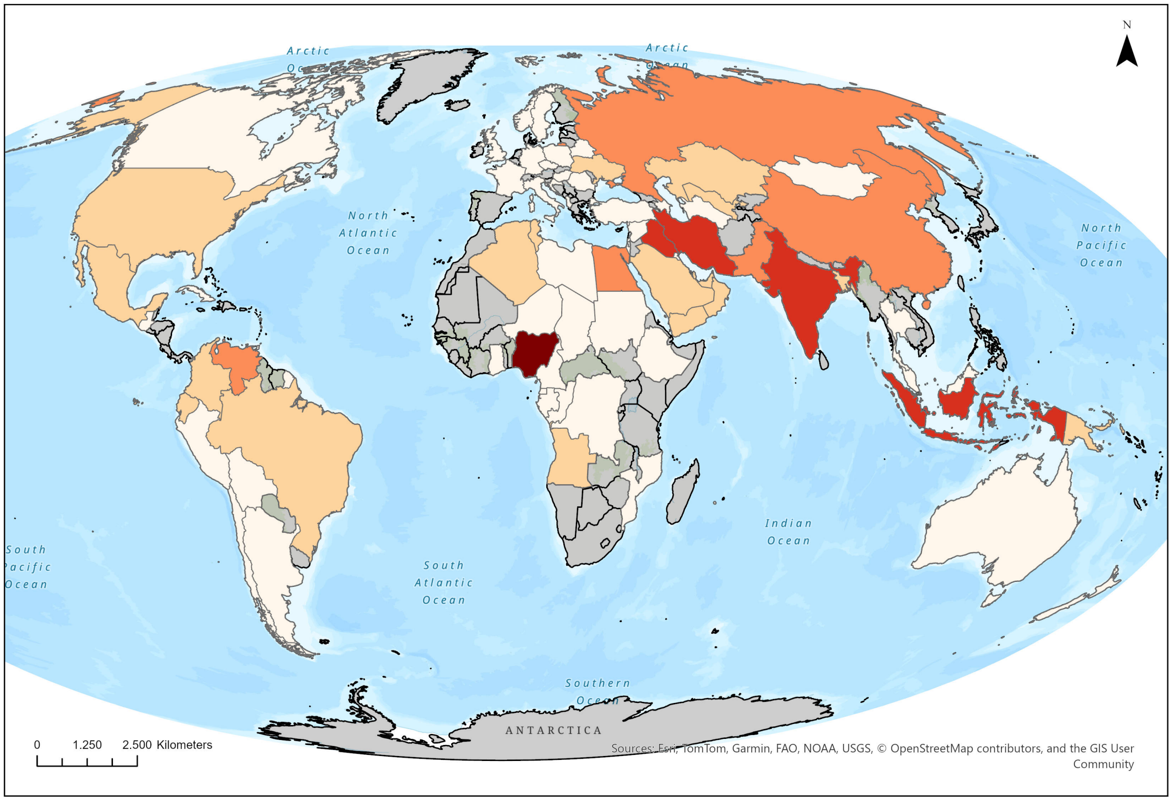

Figure S6: Global map of the maximum number of people residing within 1 km of active upstream flaring site for the year 2018

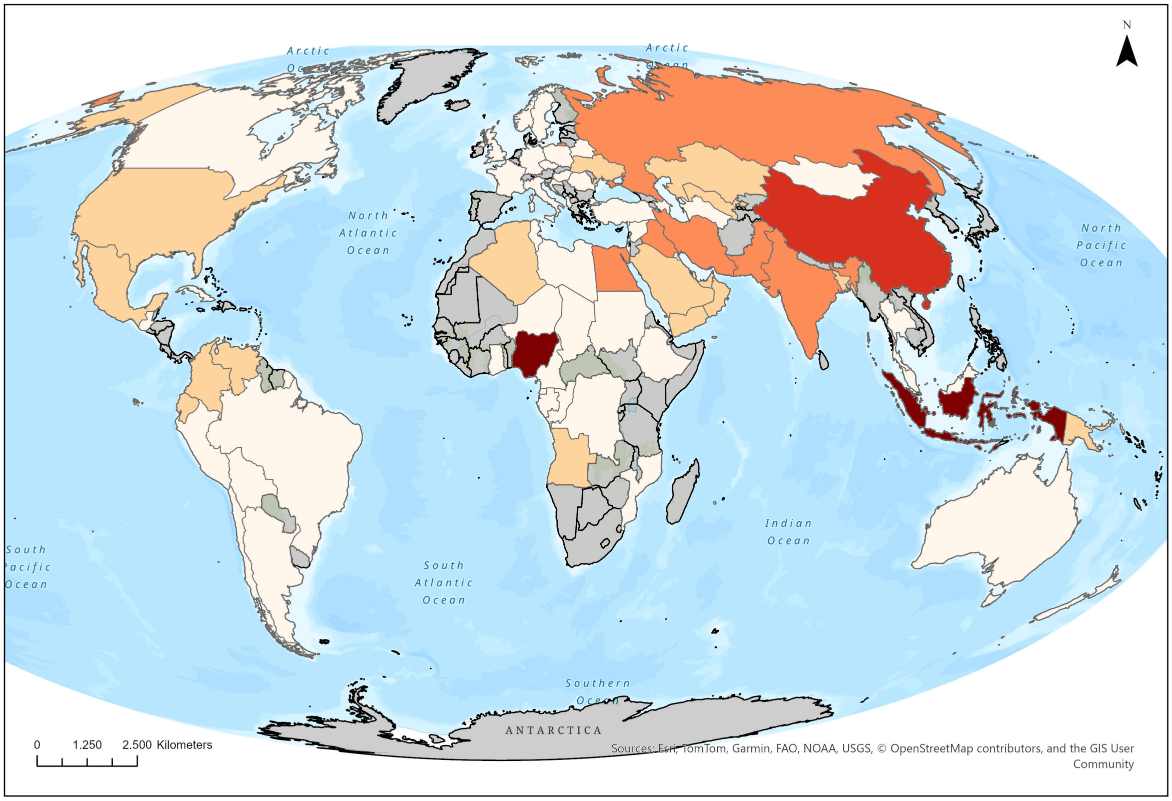

Figure S7: Global map of the maximum number of people residing within 1 km of active upstream flaring site for the year 2017

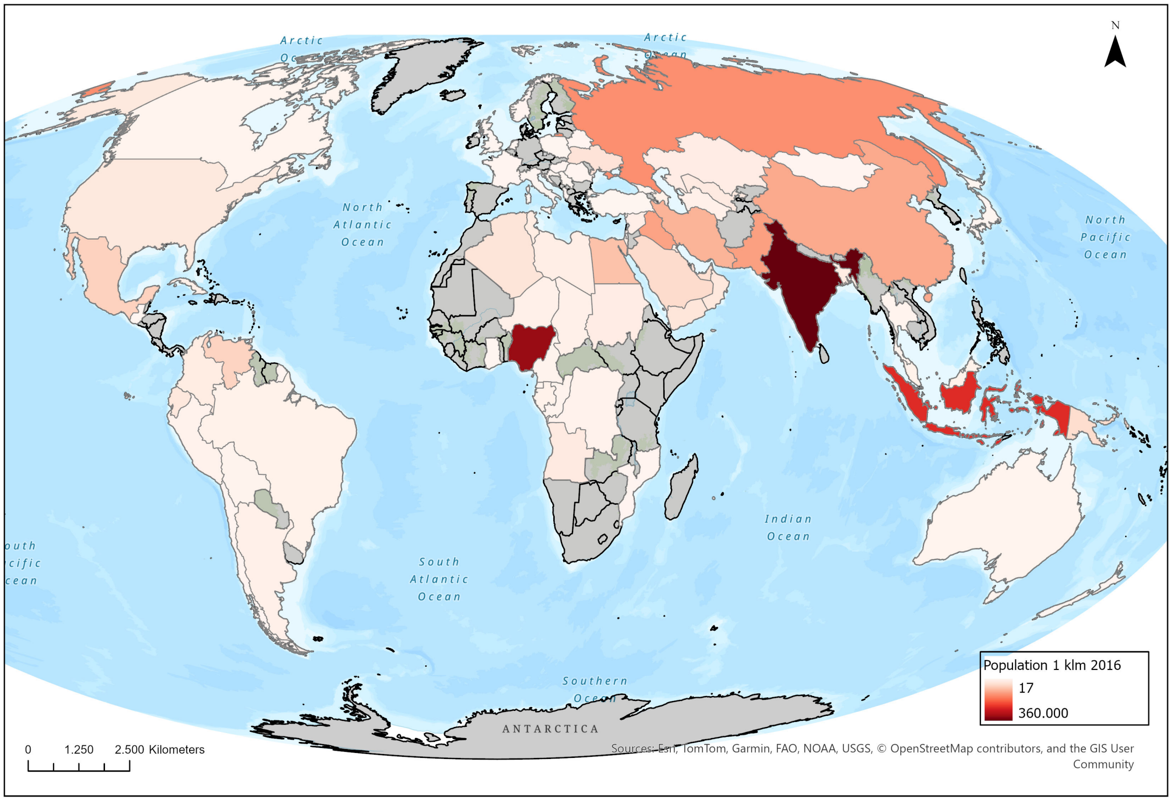

Figure S8: Global map of the maximum number of people residing within 1 km of active upstream flaring site for the year 2016

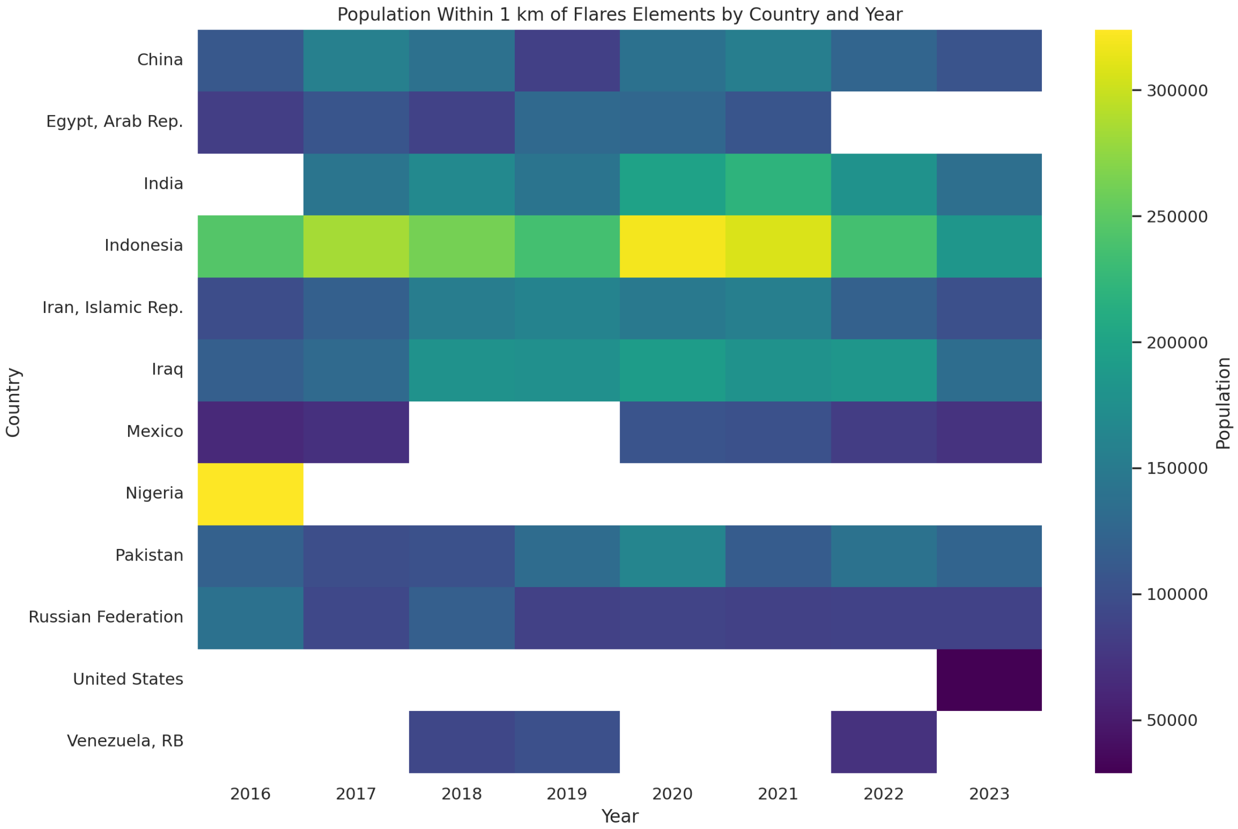

Figure S9: Heatmap of Population Near Flares by Country and Year

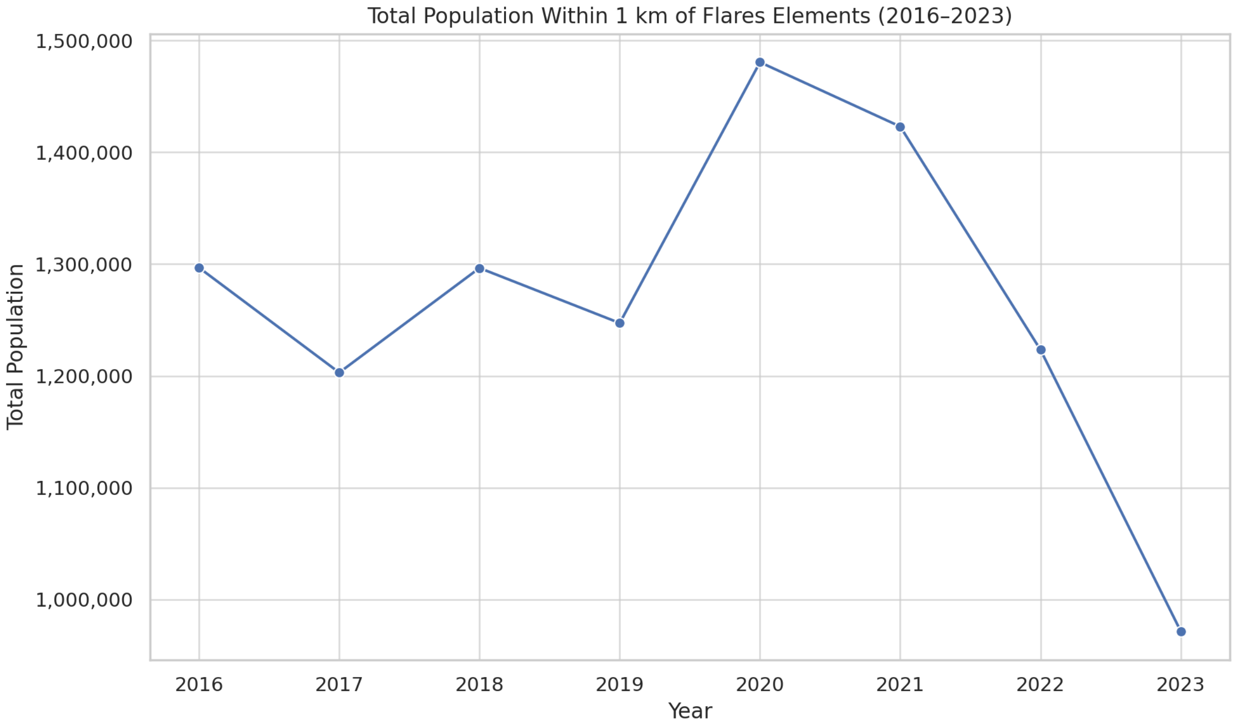

Figure S10: Total Population Within 1 km of Flares (2016–2023)

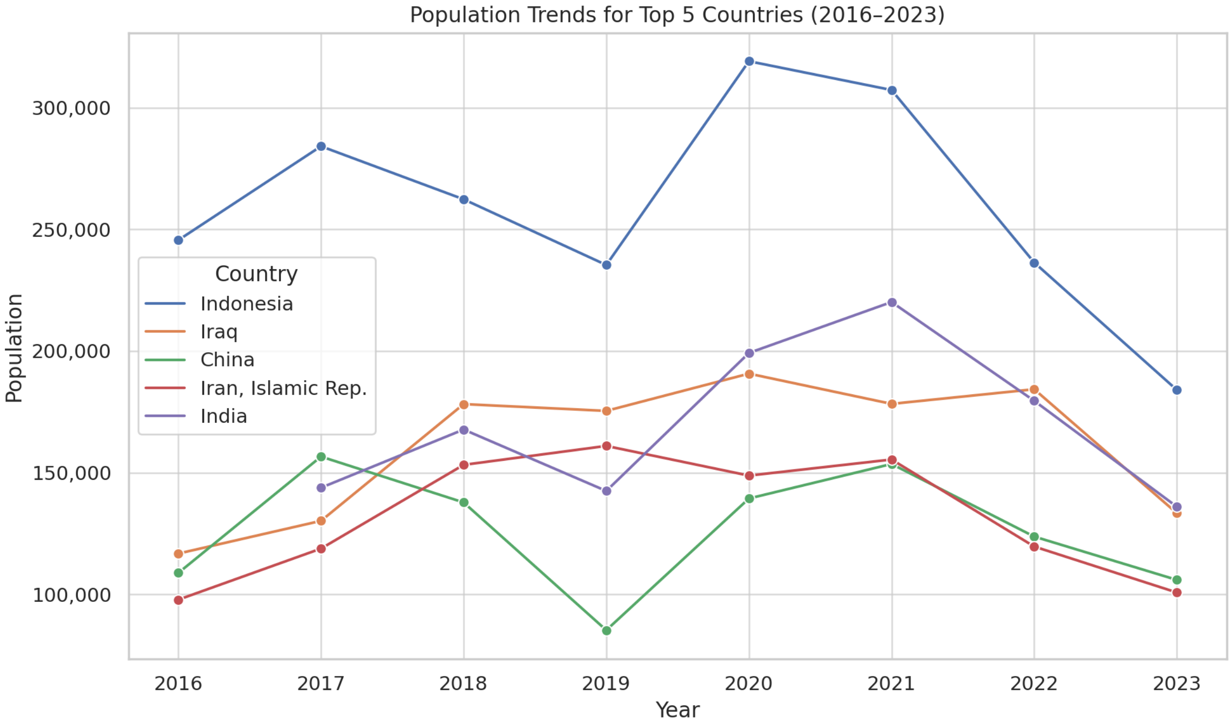

Figure S11: Top 5 Countries by Population Near Flares (2016–2023)

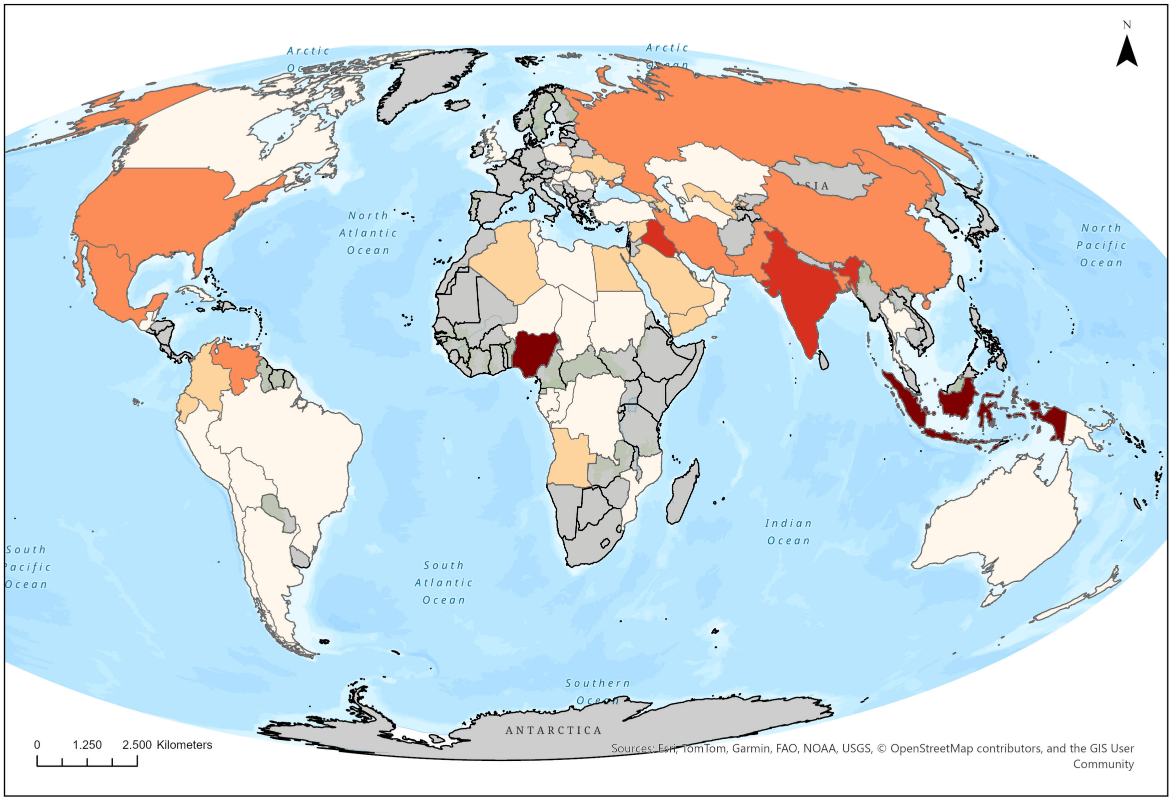

Figure S12: Global map of the maximum number of people residing within 3 km of active upstream flaring site for the year 2023

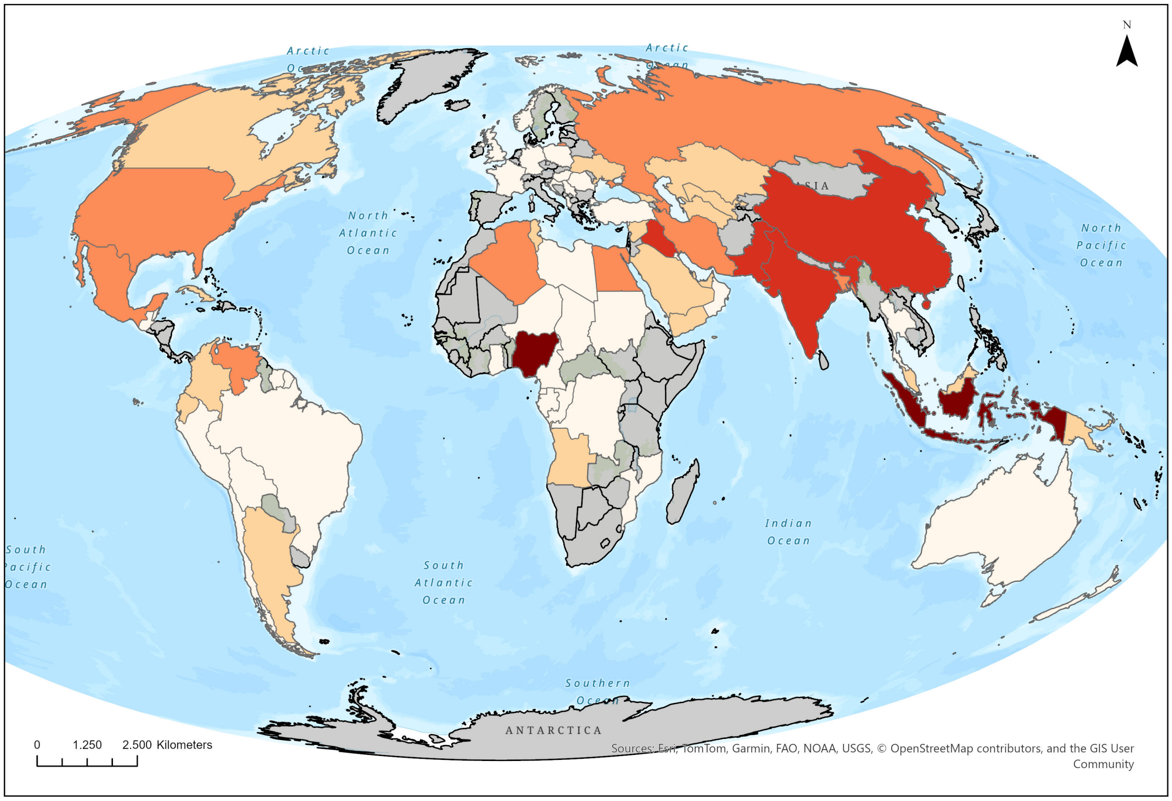

Figure S13: Global map of the maximum number of people residing within 3 km of active upstream flaring site for the year 2022

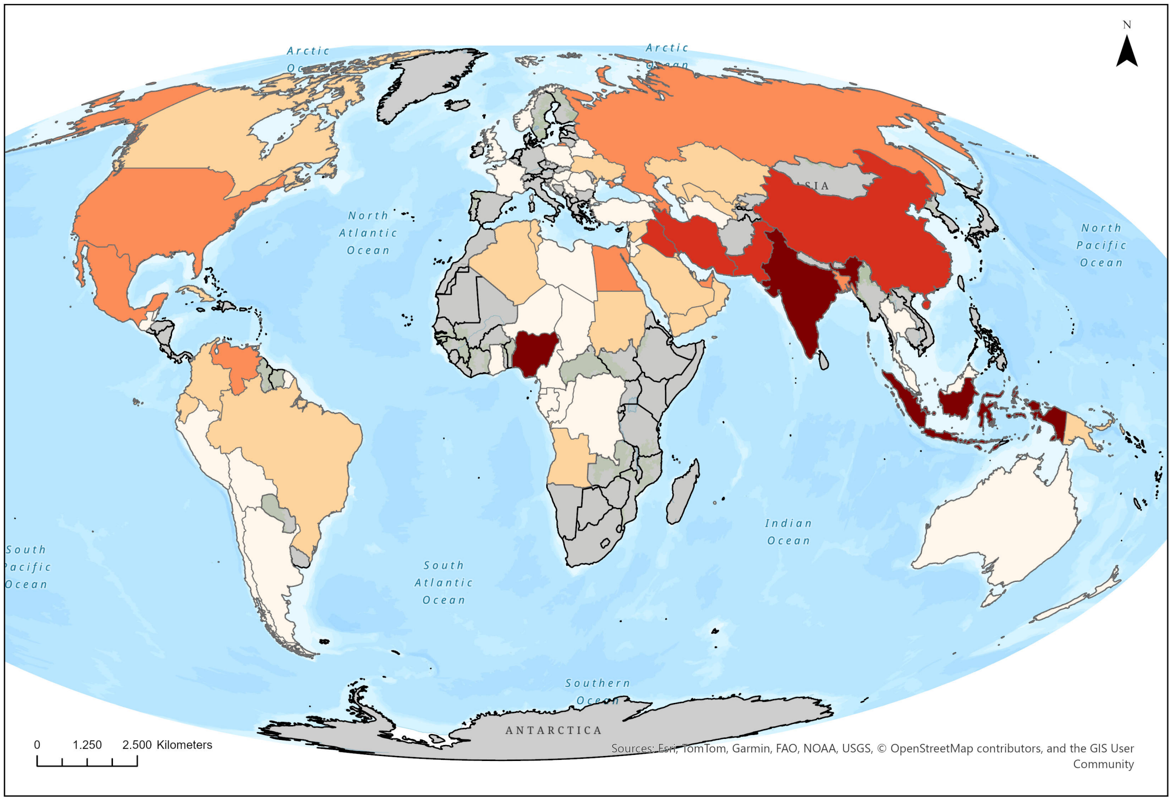

Figure S14: Global map of the maximum number of people residing within 3 km of active upstream flaring site for the year 2021

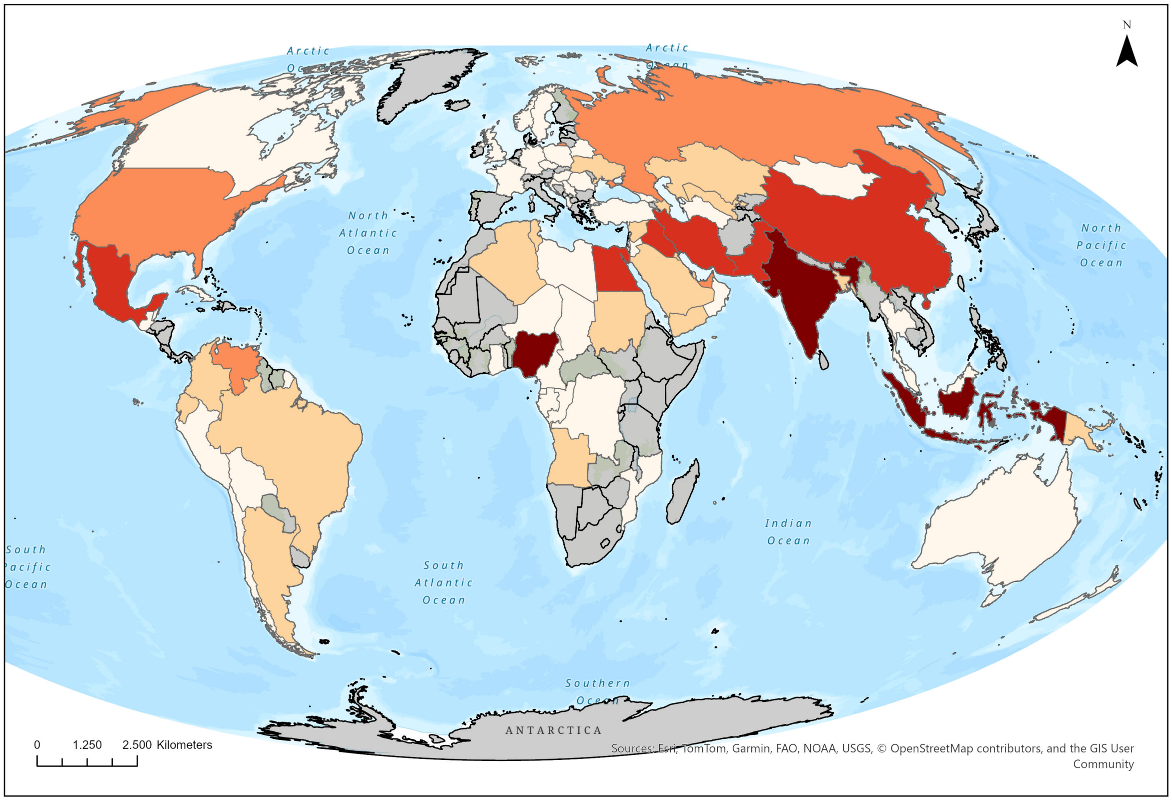

Figure S15: Global map of the maximum number of people residing within 3 km of active upstream flaring site for the year 2020

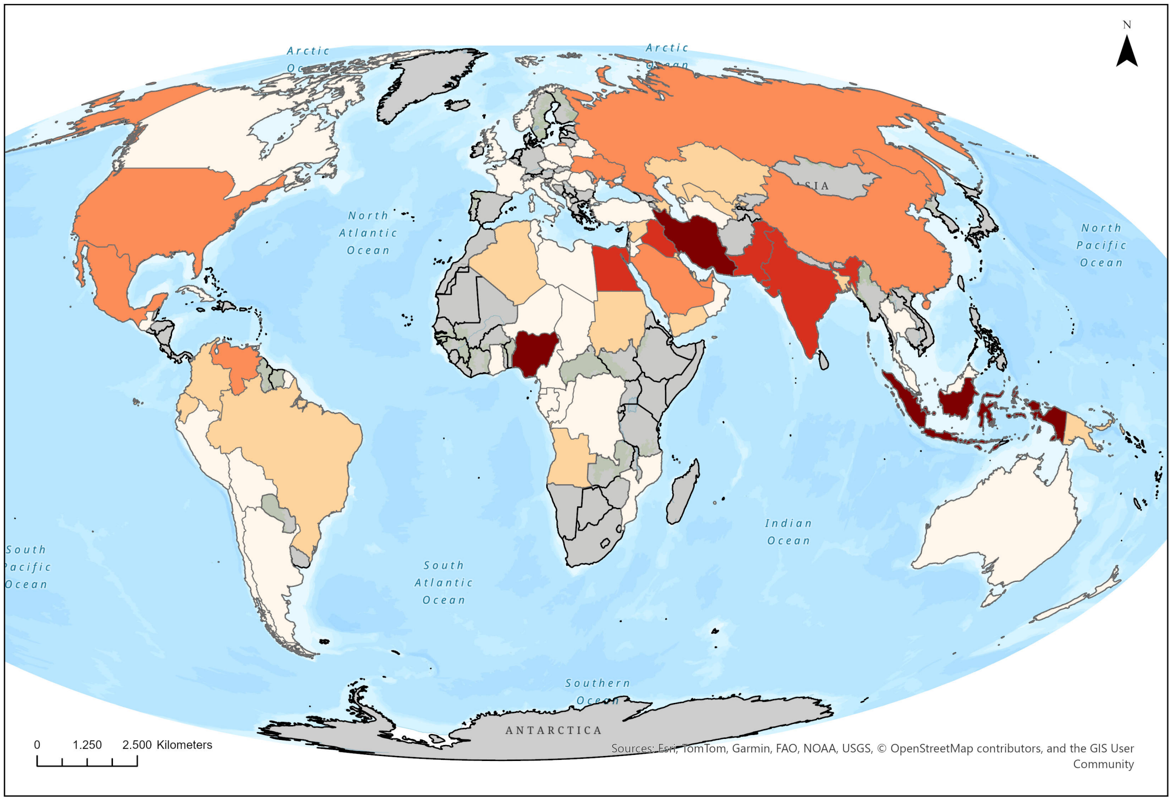

Figure S16: Global map of the maximum number of people residing within 3 km of active upstream flaring site for the year 2019

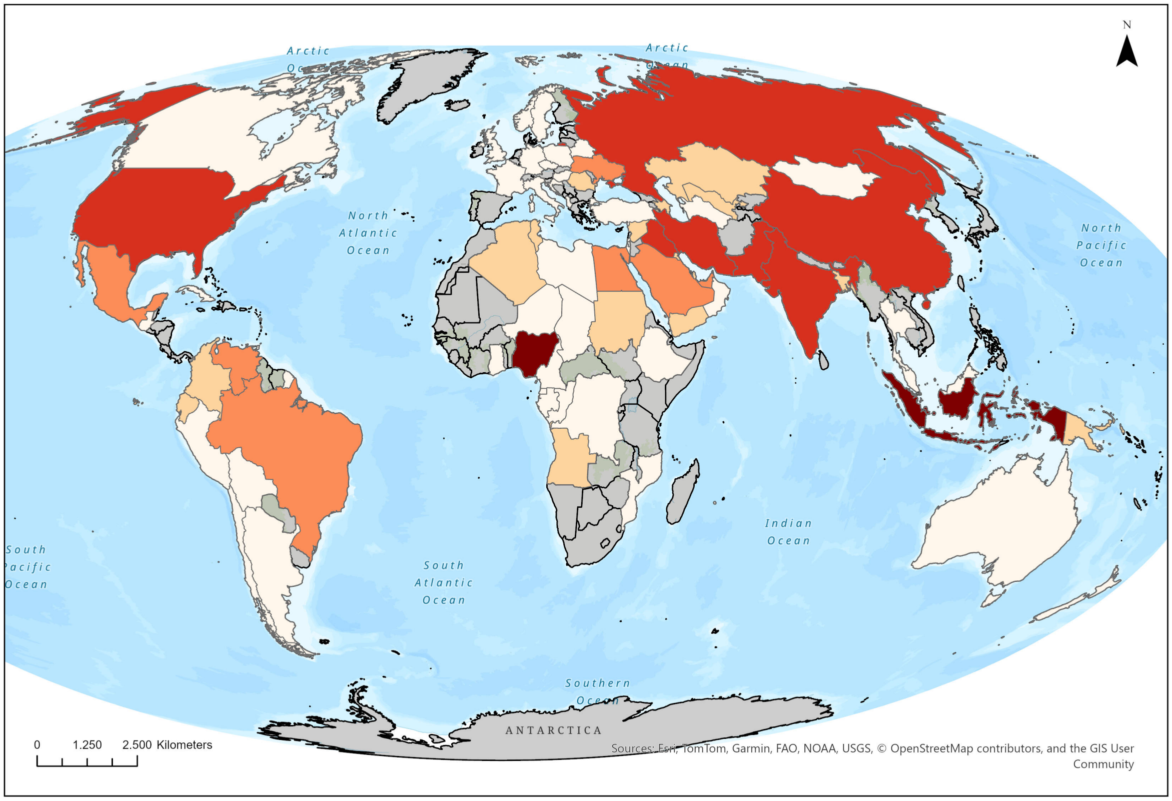

Figure S17: Global map of the maximum number of people residing within 3 km of active upstream flaring site for the year 2018

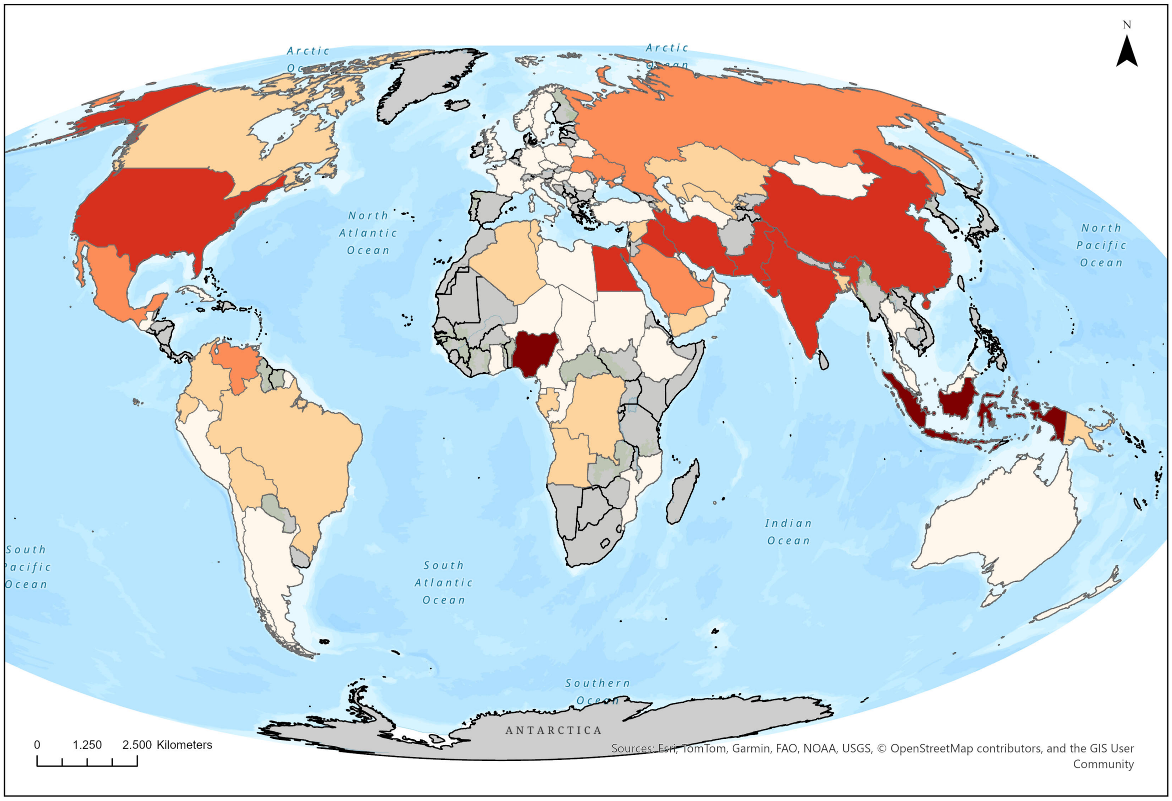

Figure S18: Global map of the maximum number of people residing within 3 km of active upstream flaring site for the year 2017

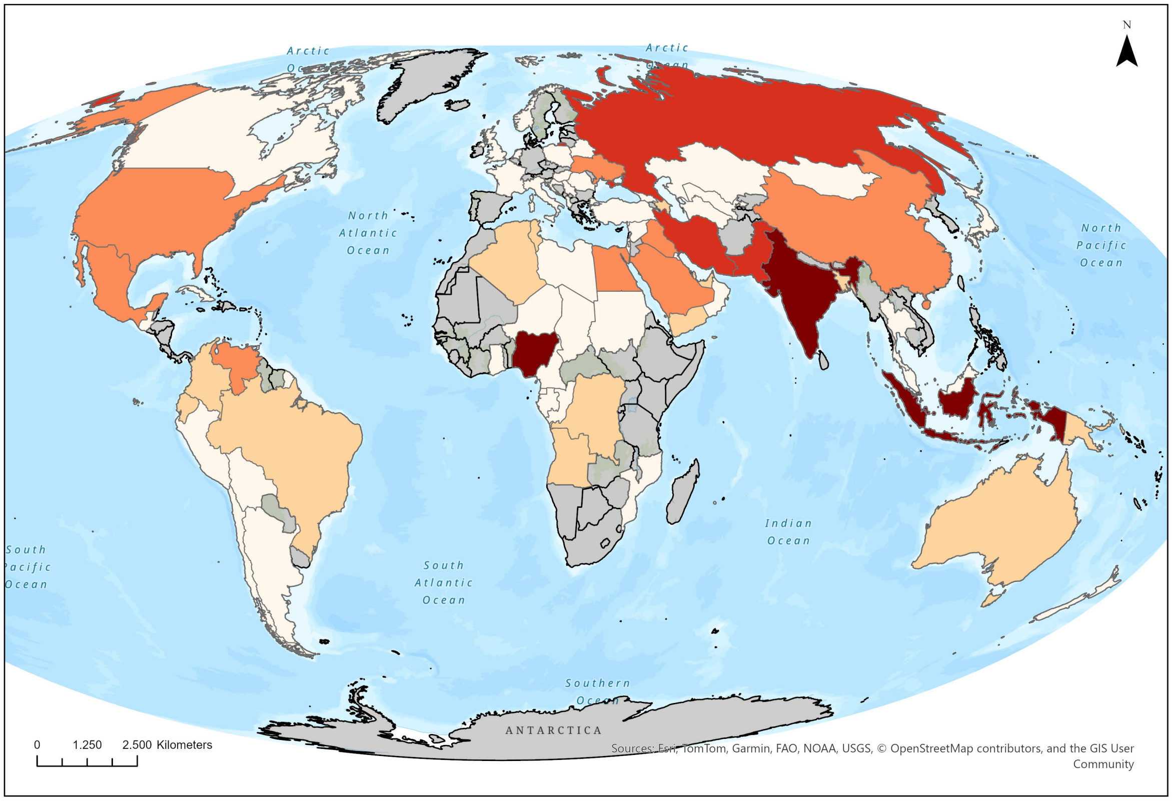

Figure S19: Global map of the maximum number of people residing within 3 km of active upstream flaring site for the year 2016

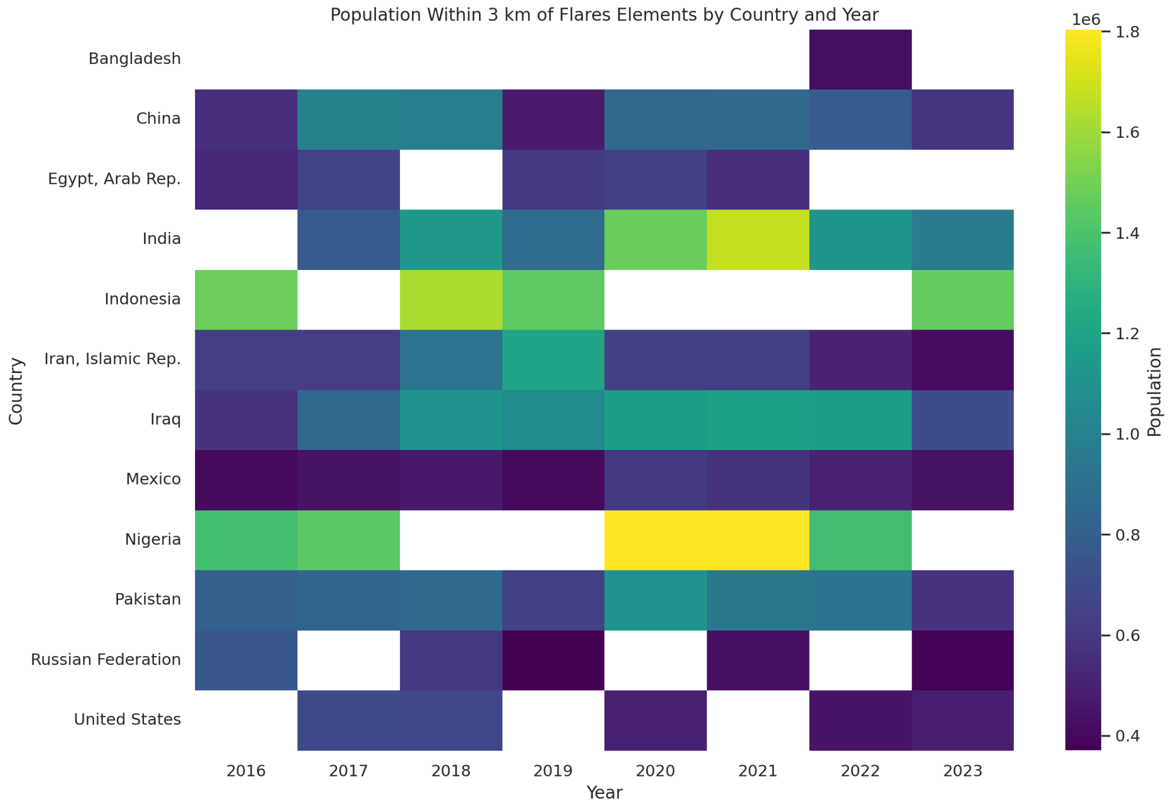

Figure S20: Heatmap of Population Near Flares by Country and Year

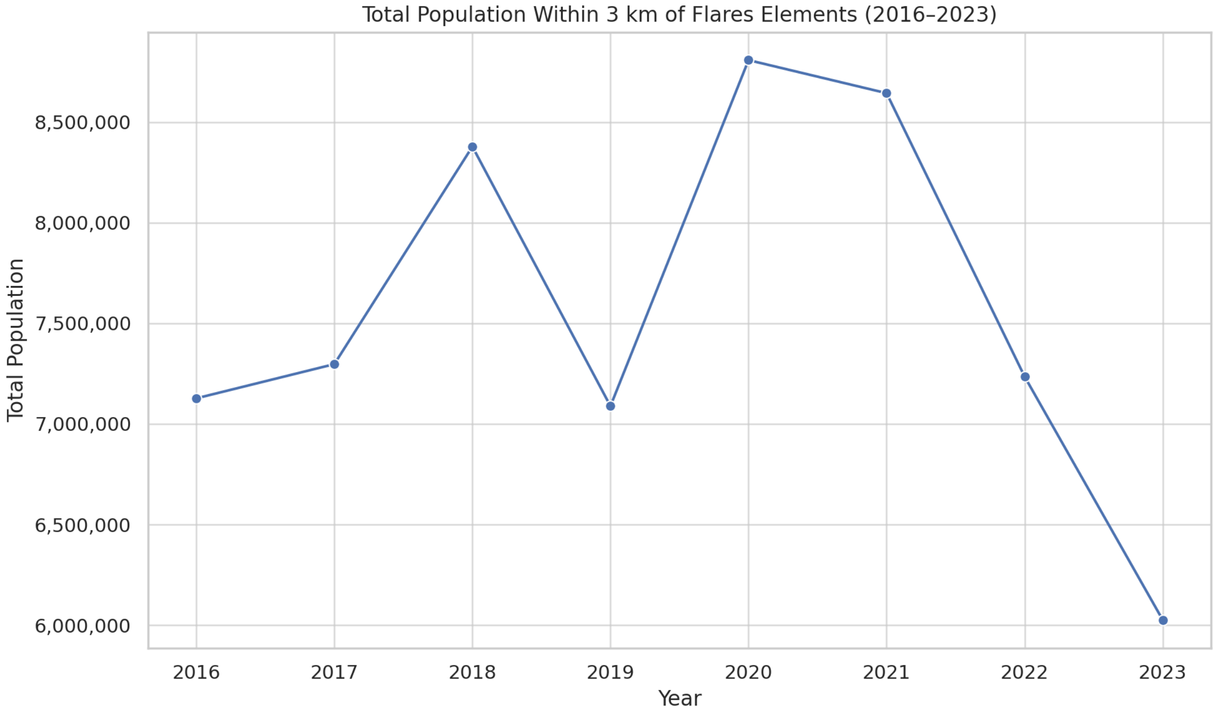

Figure S21: Total Population Within 3 km of Flares (2016–2023)

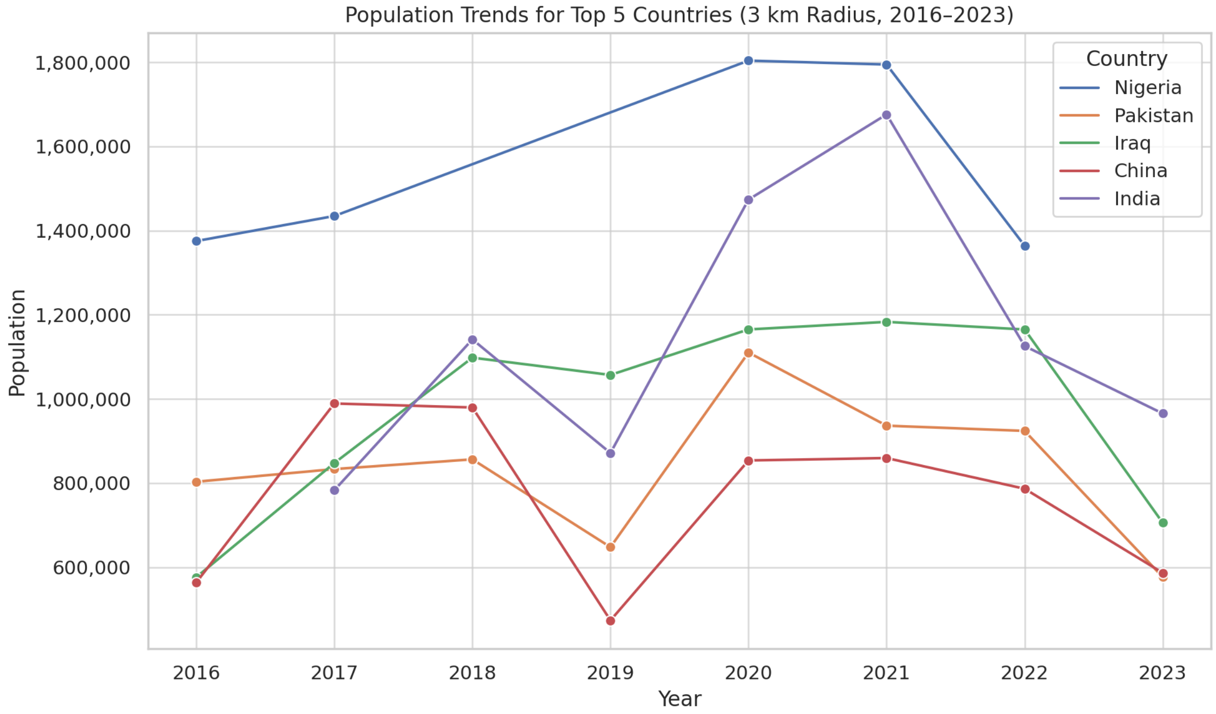

Figure S22: Top 5 Countries by Population Near Flares (2016–2023)

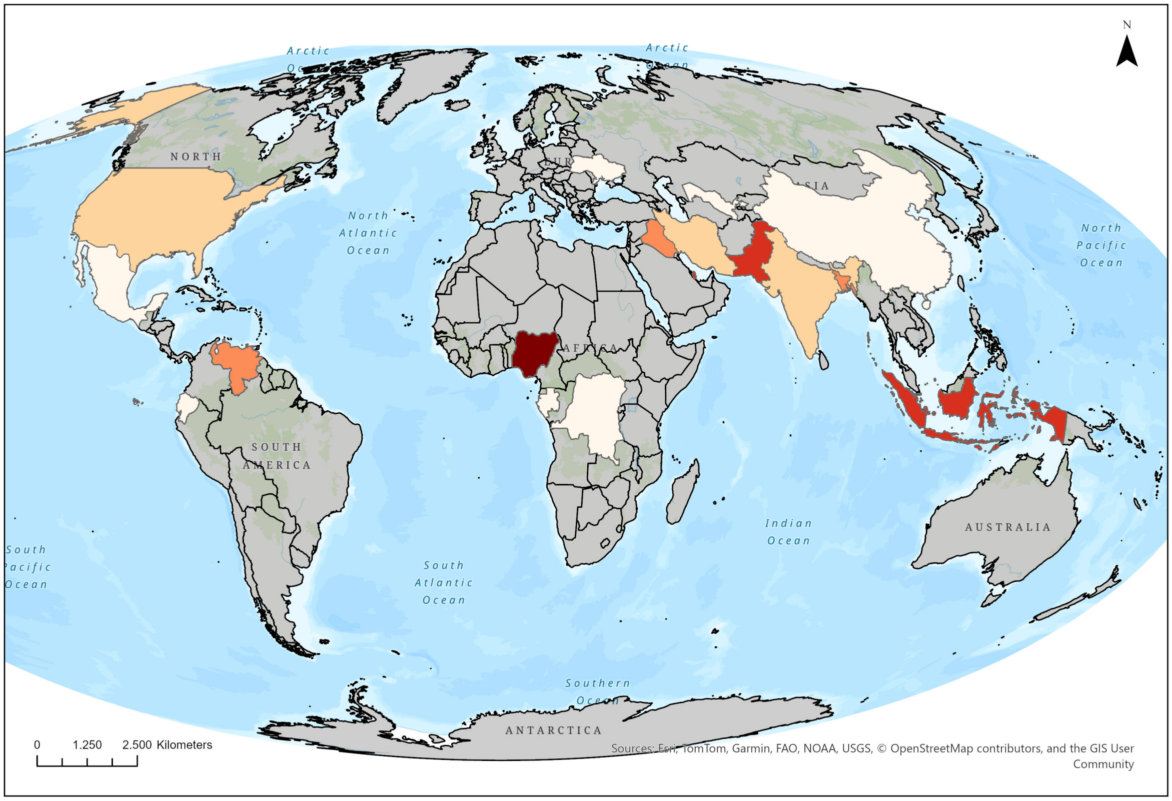

Figure S23: Global maps showing, for each country, the maximum number of people residing in urban centre areas within a 1 km radius of active flaring sites for the year 2023

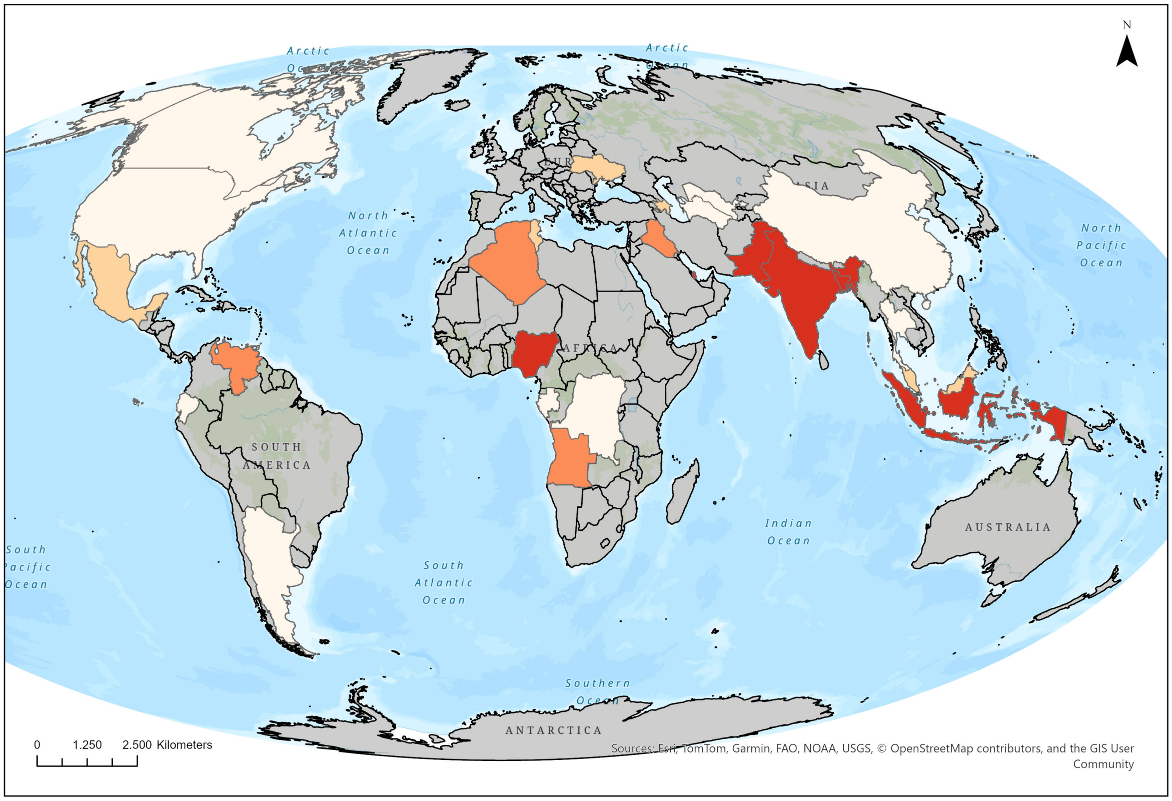

Figure S24: Global maps showing, for each country, the maximum number of people residing in urban centre areas within a 1 km radius of active flaring sites for the year 2022

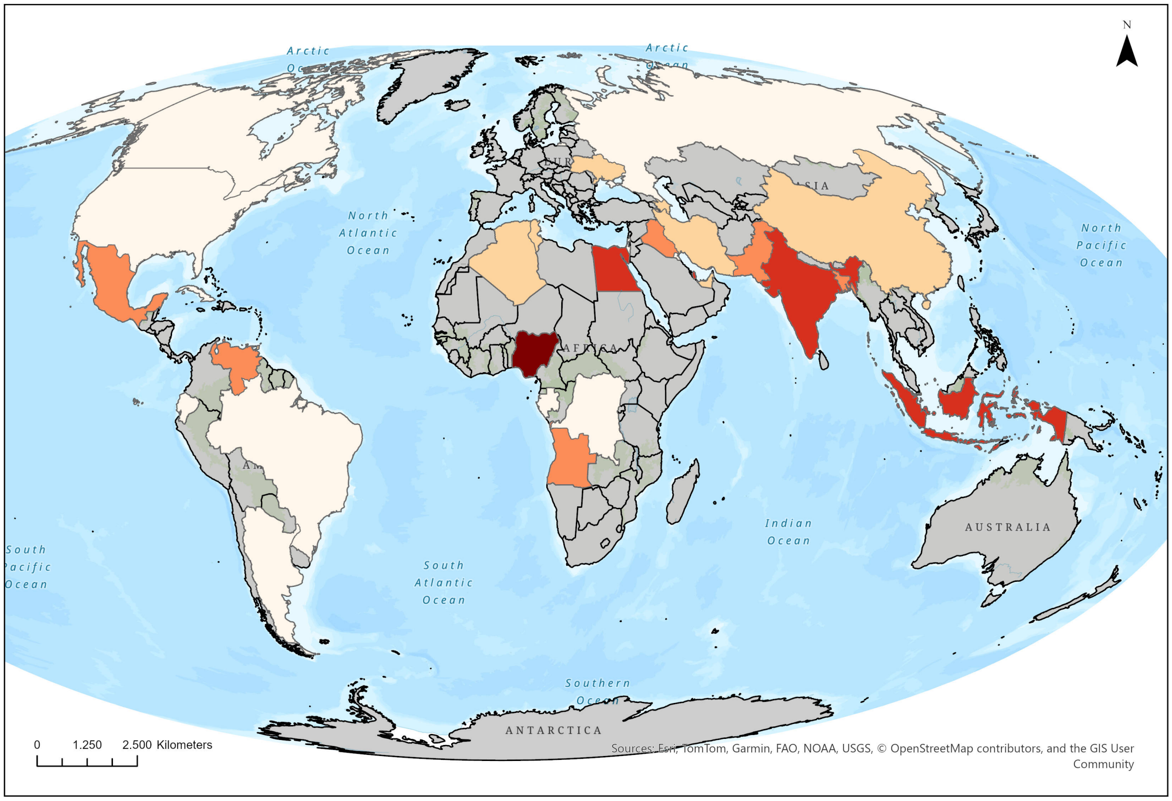

Figure S25: Global maps showing, for each country, the maximum number of people residing in urban centre areas within a 1 km radius of active flaring sites for the year 2021

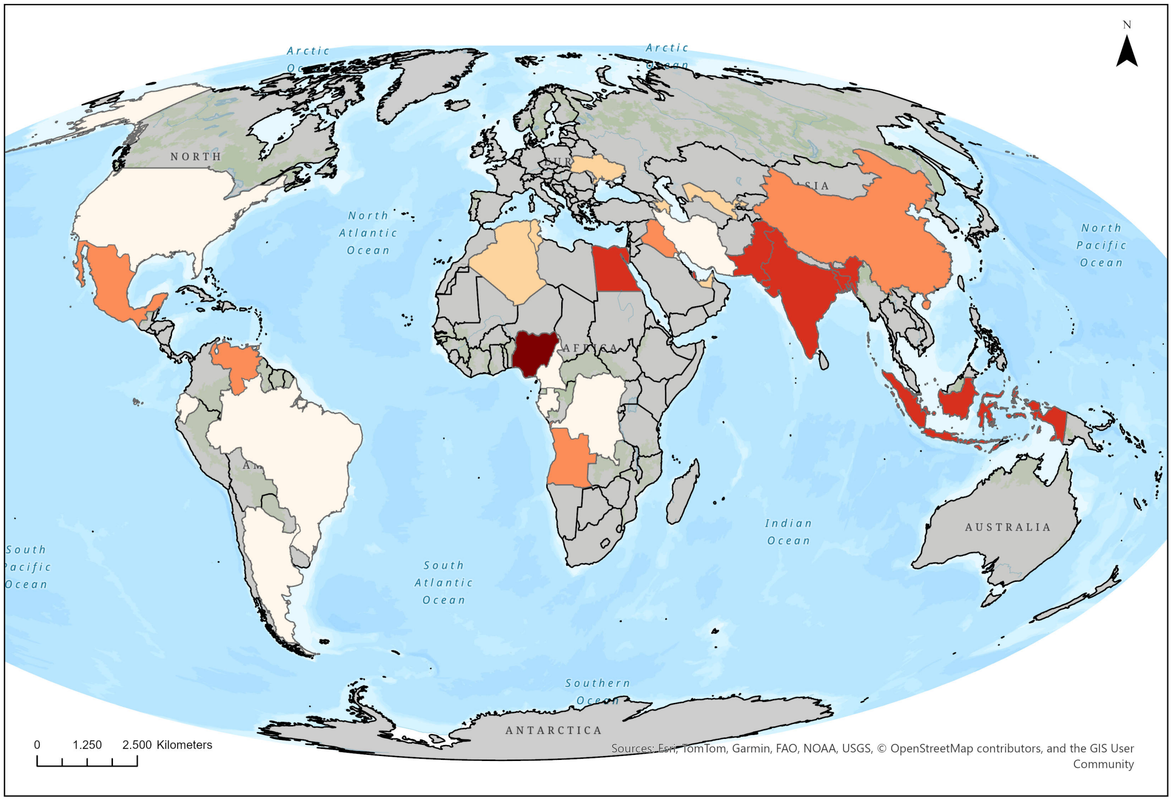

Figure S26: Global maps showing, for each country, the maximum number of people residing in urban centre areas within a 1 km radius of active flaring sites for the year 2020

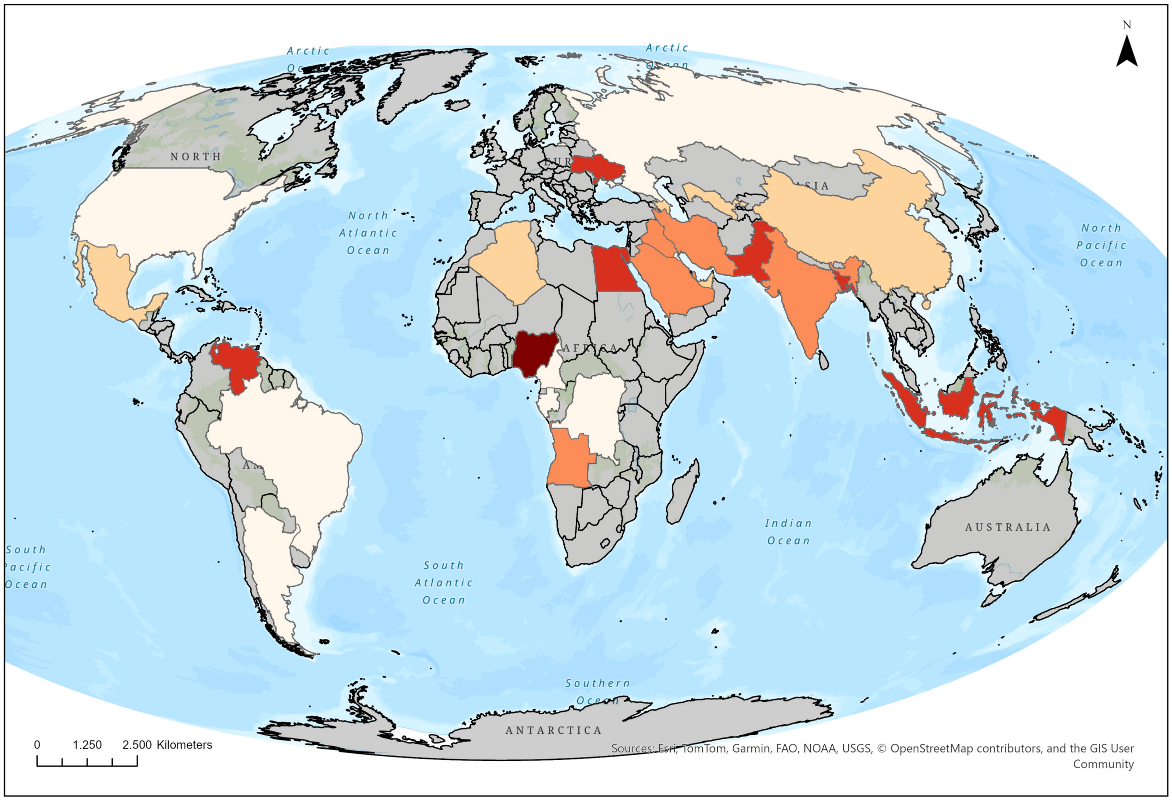

Figure S27: Global maps showing, for each country, the maximum number of people residing in urban centre areas within a 1 km radius of active flaring sites for the year 2019

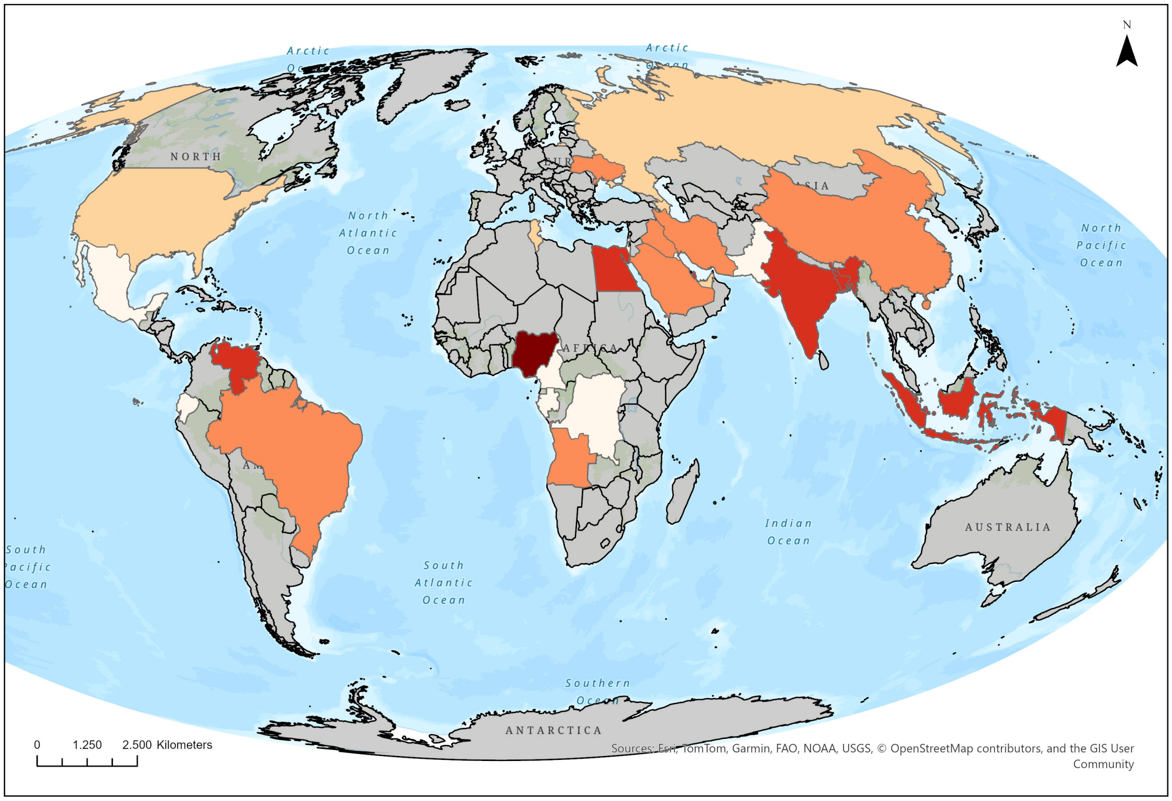

Figure S28: Global maps showing, for each country, the maximum number of people residing in urban centre areas within a 1 km radius of active flaring sites for the year 2018

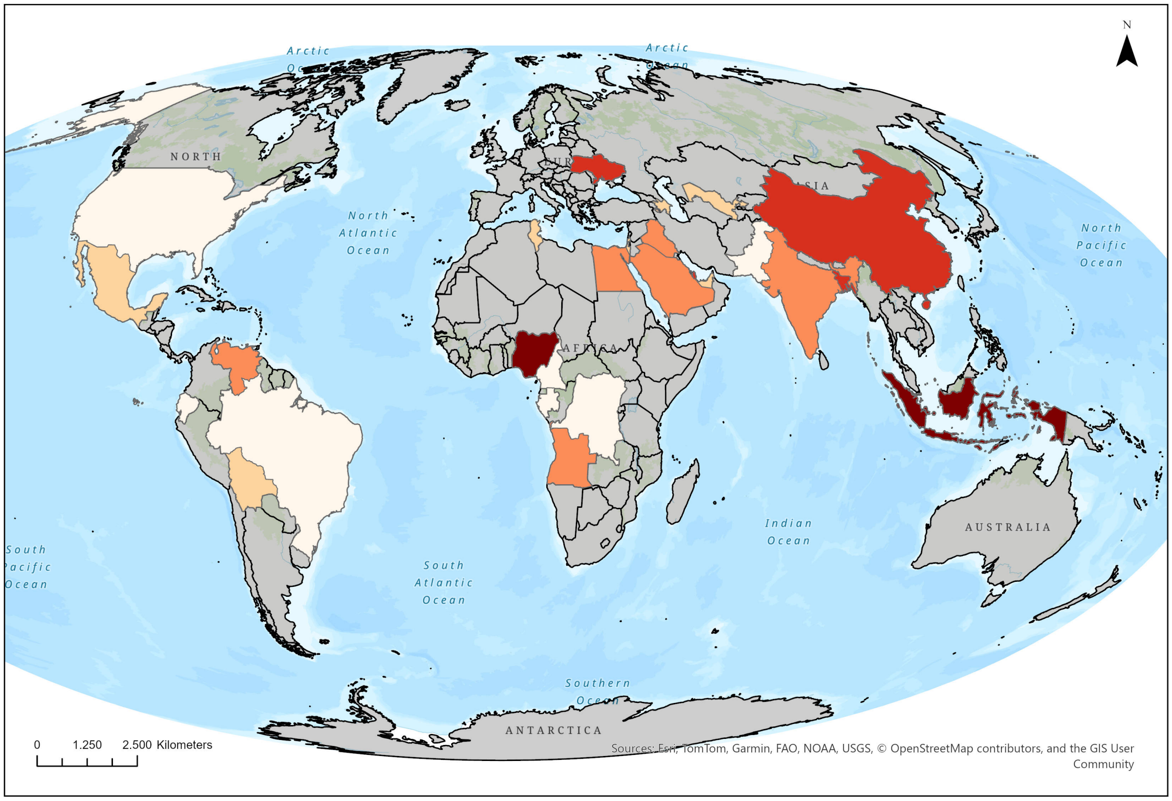

Figure S29: Global maps showing, for each country, the maximum number of people residing in urban centre areas within a 1 km radius of active flaring sites for the year 2017

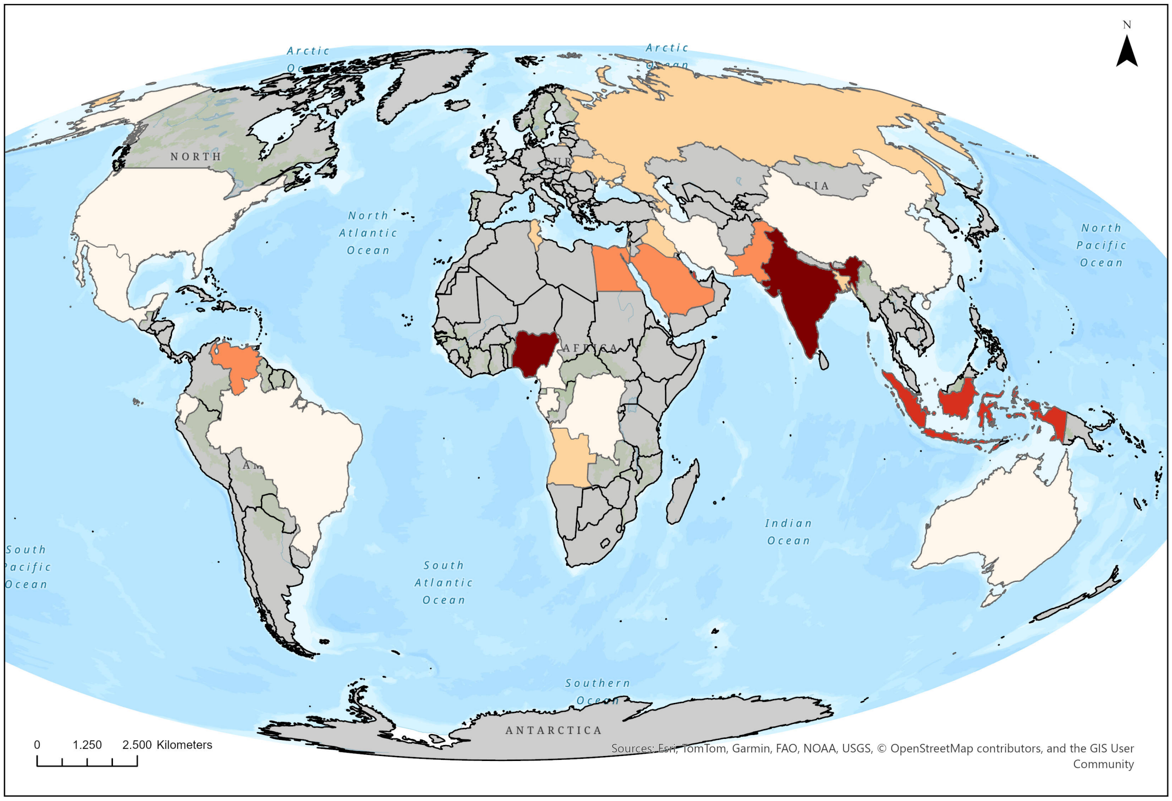

Figure S30: Global maps showing, for each country, the maximum number of people residing in urban centre areas within a 1 km radius of active flaring sites for the year 2016

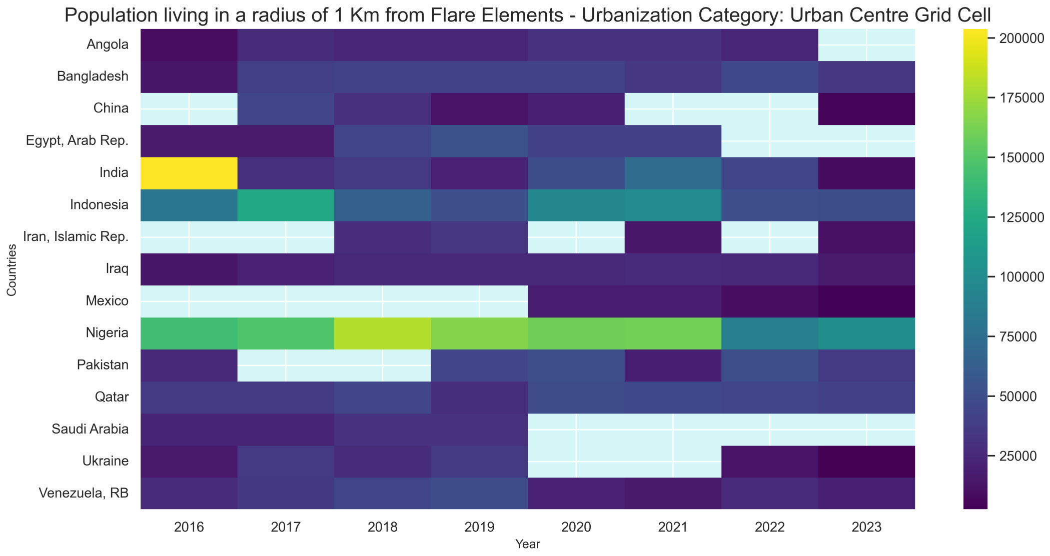

Figure S31: Heatmap of Urban Centre Population (within 1 km of flares) — Top 15 Countries, 2016–2023

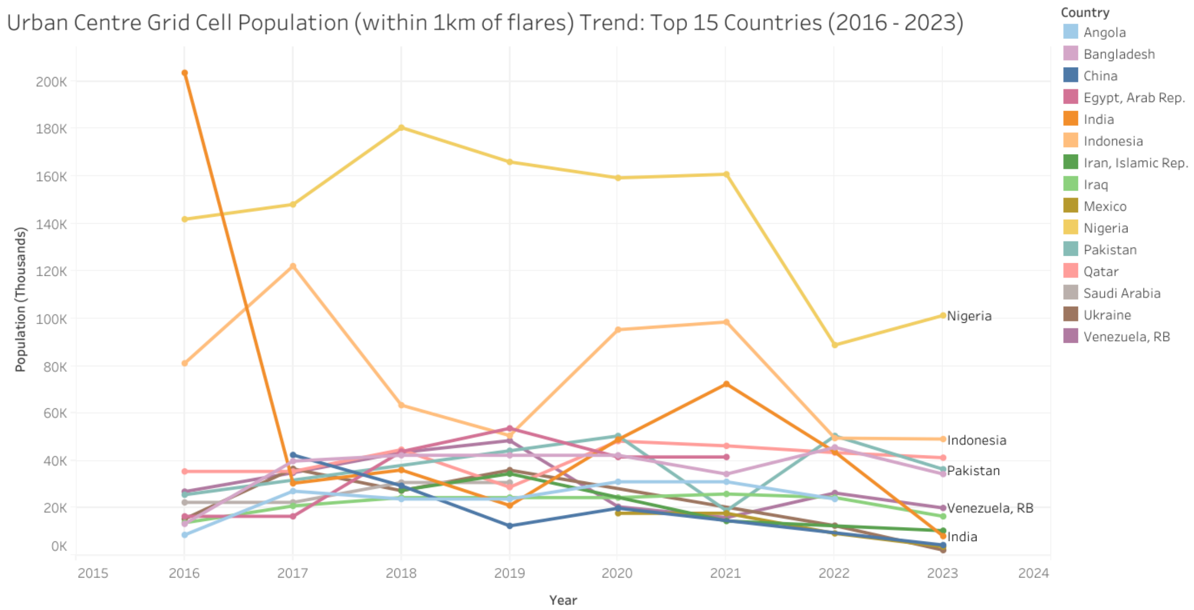

Figure S32: Urban Centre Population (within 1 km of flares) — Top 15 Countries, 2016–2023

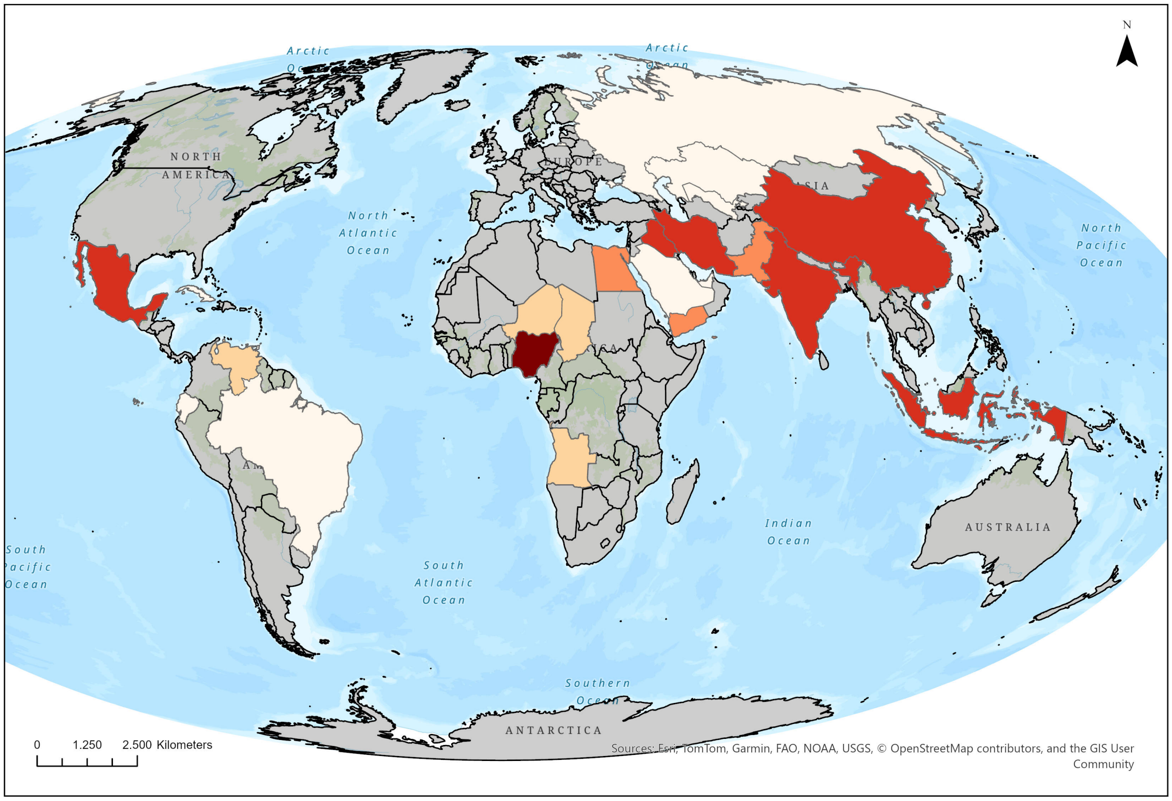

Figure S33: Global maps showing, for each country, the maximum number of people residing in dense urban areas within a 1 km radius of active flaring sites for the year 2023

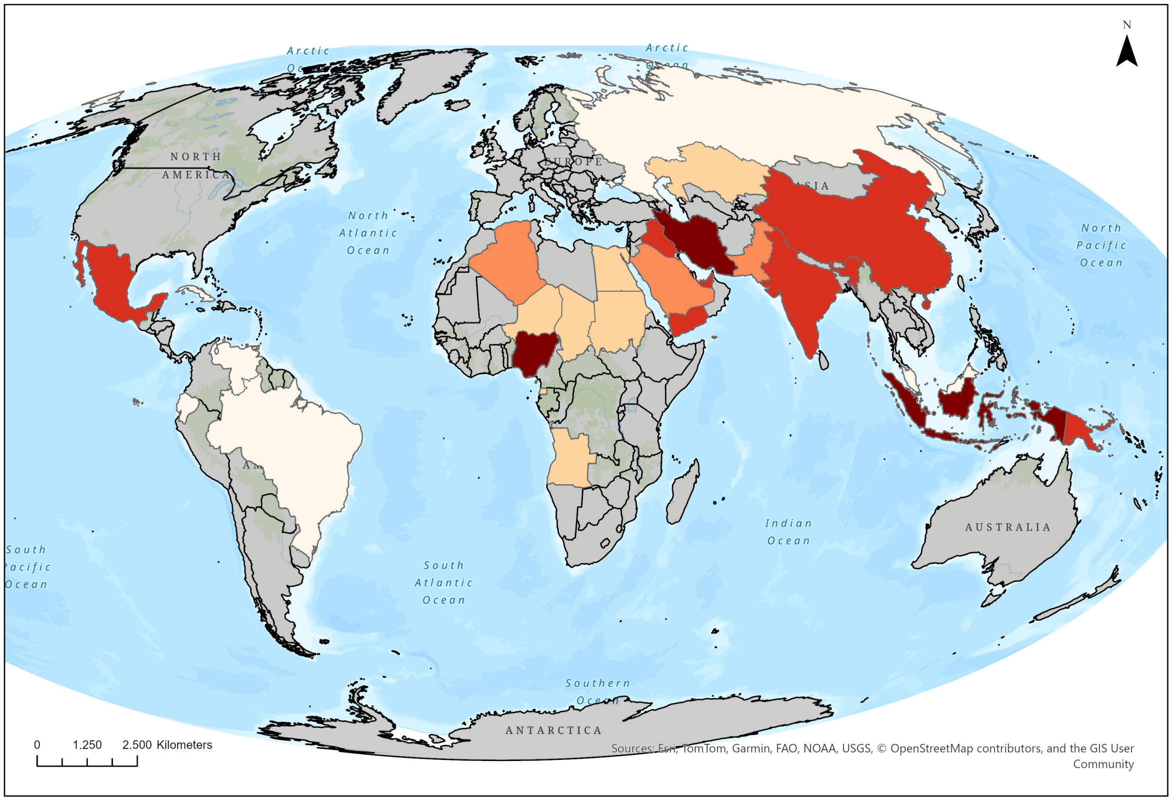

Figure S34: Global maps showing, for each country, the maximum number of people residing in dense urban areas within a 1 km radius of active flaring sites for the year 2022

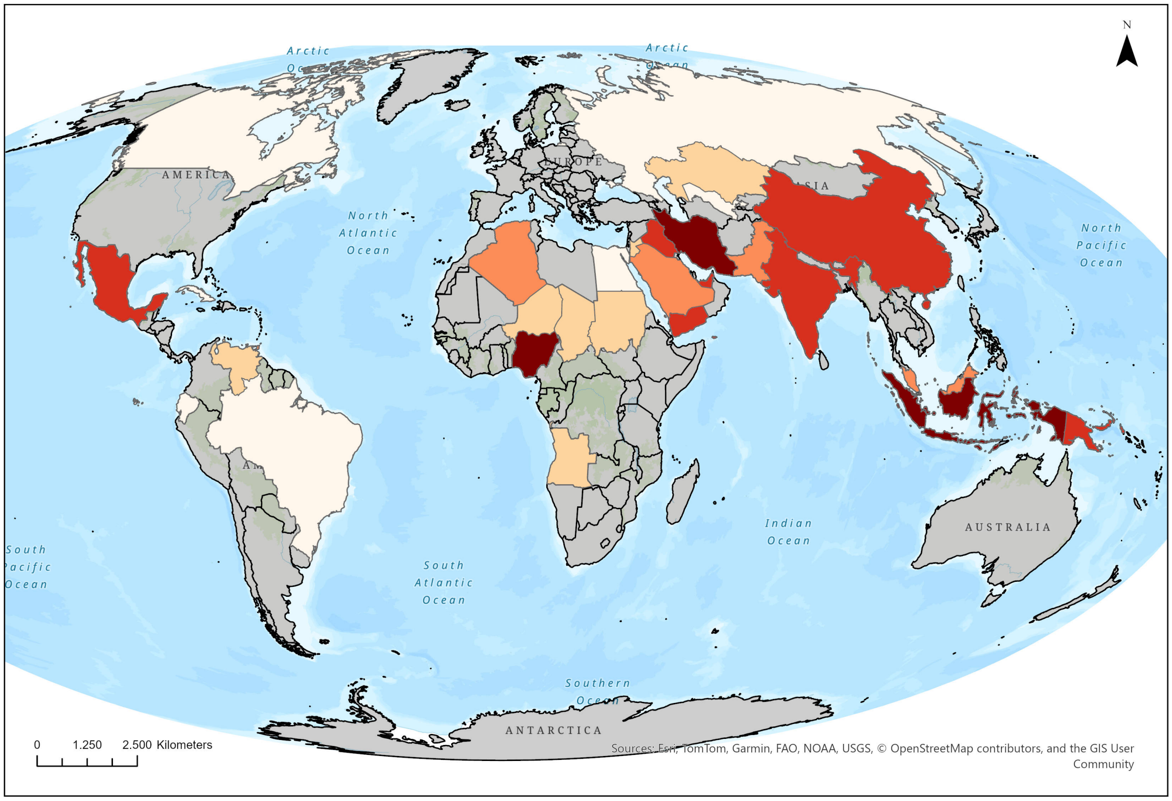

Figure S35: Global maps showing, for each country, the maximum number of people residing in dense urban areas within a 1 km radius of active flaring sites for the year 2021

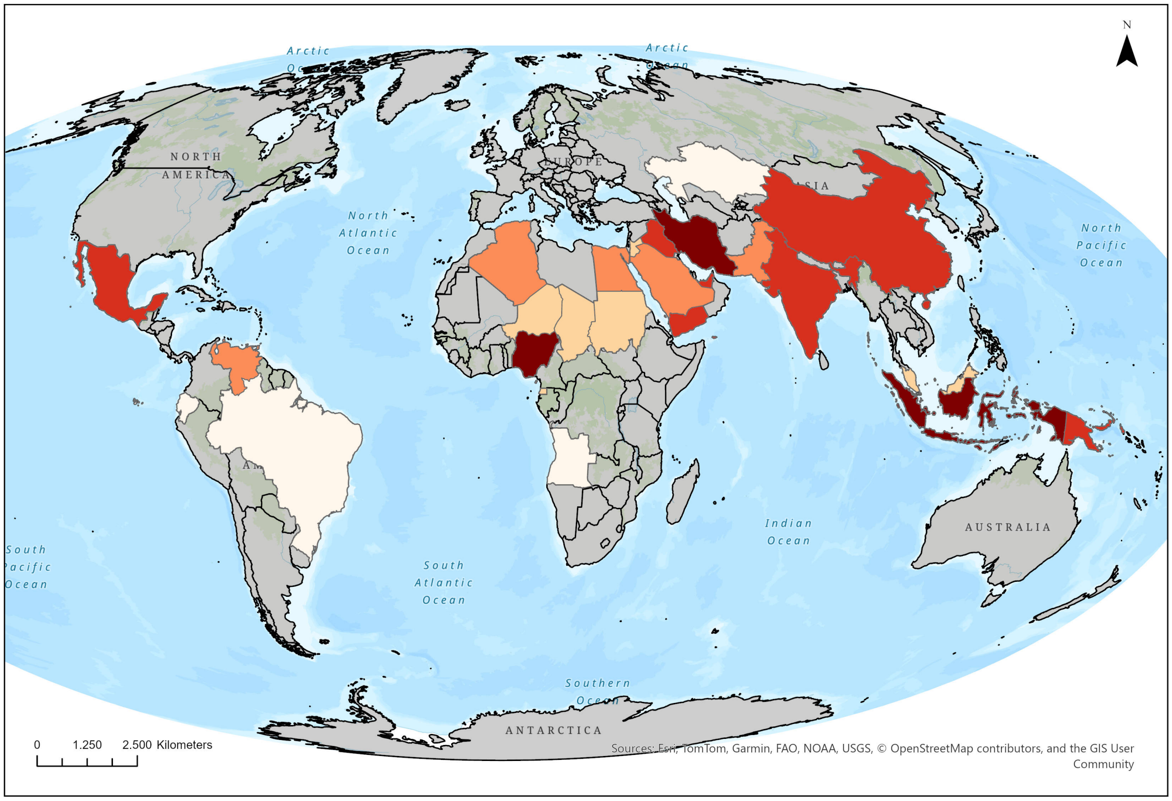

Figure S36: Global maps showing, for each country, the maximum number of people residing in dense urban areas within a 1 km radius of active flaring sites for the year 2020

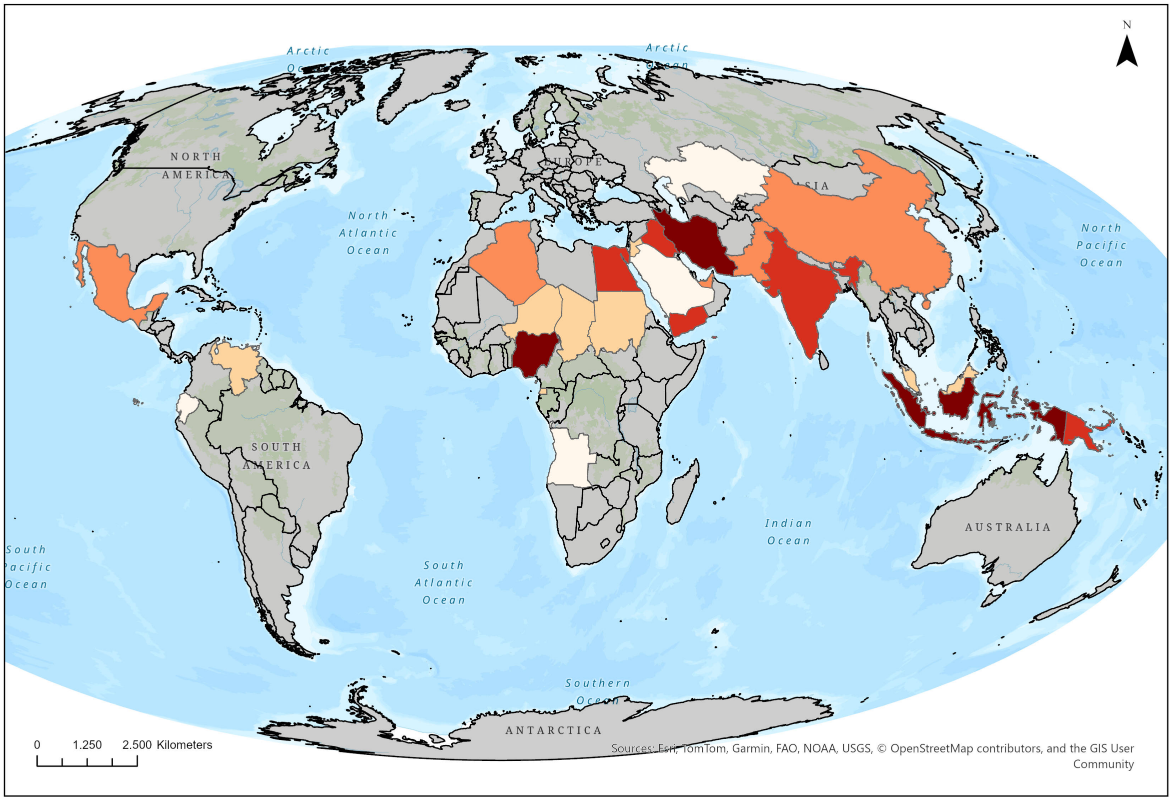

Figure S37: Global maps showing, for each country, the maximum number of people residing in dense urban areas within a 1 km radius of active flaring sites for the year 2019

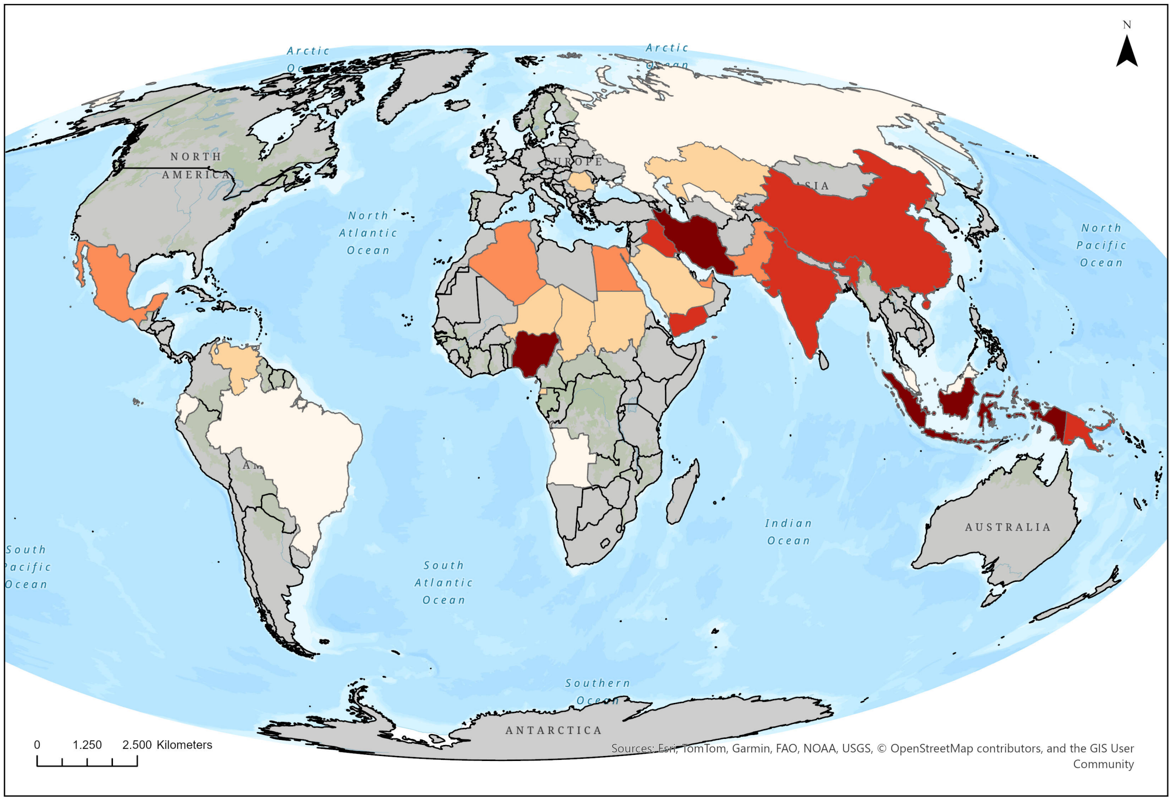

Figure S38: Global maps showing, for each country, the maximum number of people residing in dense urban areas within a 1 km radius of active flaring sites for the year 2018

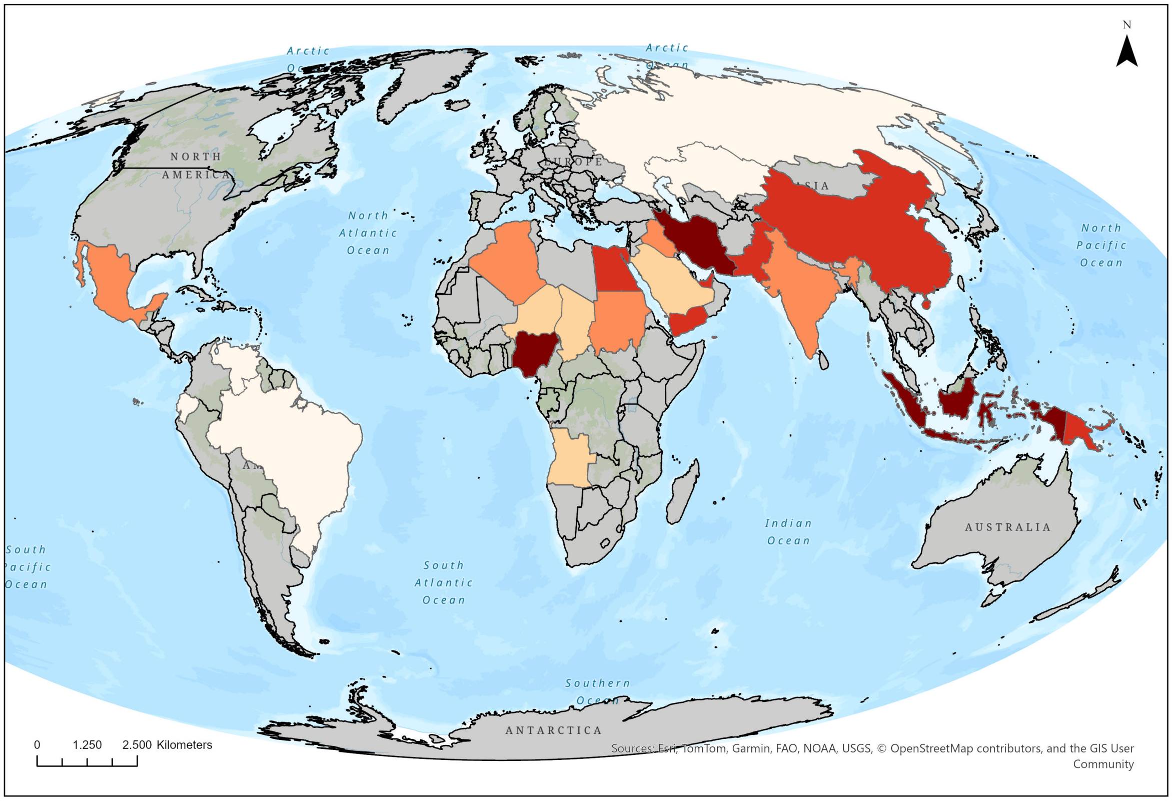

Figure S39: Global maps showing, for each country, the maximum number of people residing in dense urban areas within a 1 km radius of active flaring sites for the year 2017

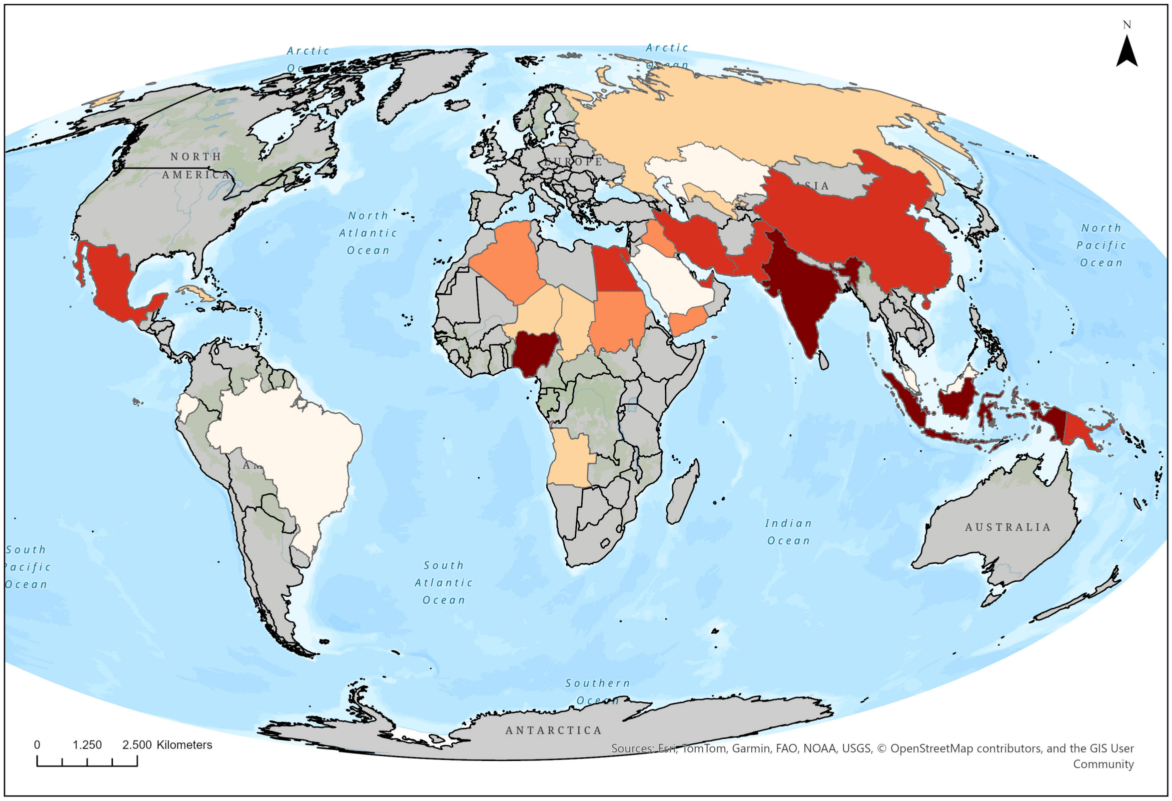

Figure S40: Global maps showing, for each country, the maximum number of people residing in dense urban areas within a 1 km radius of active flaring sites for the year 2016

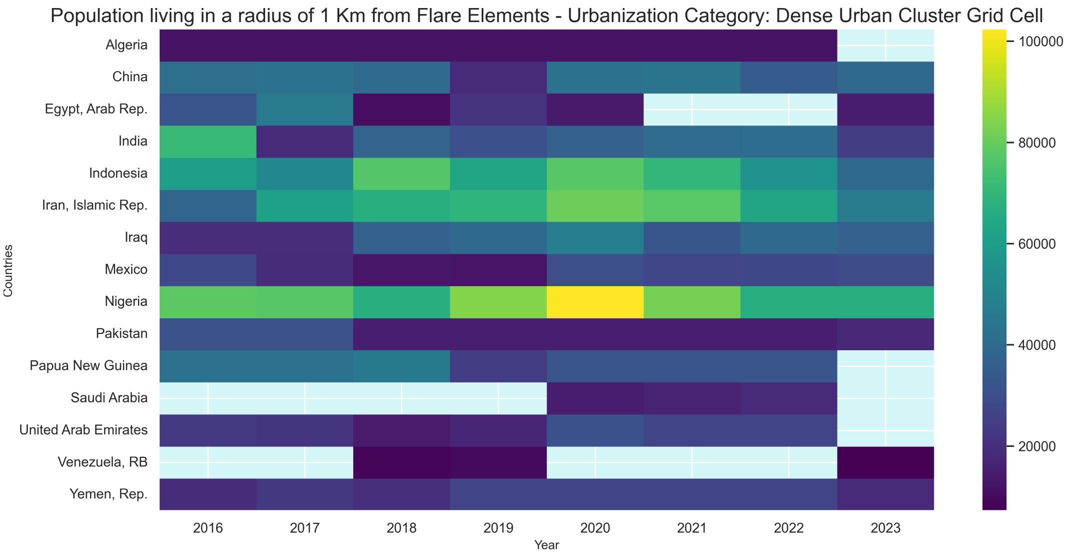

Figure S41: Heatmap of dense urban population (within 1 km of flares) for the top 15 countries, 2016–2023

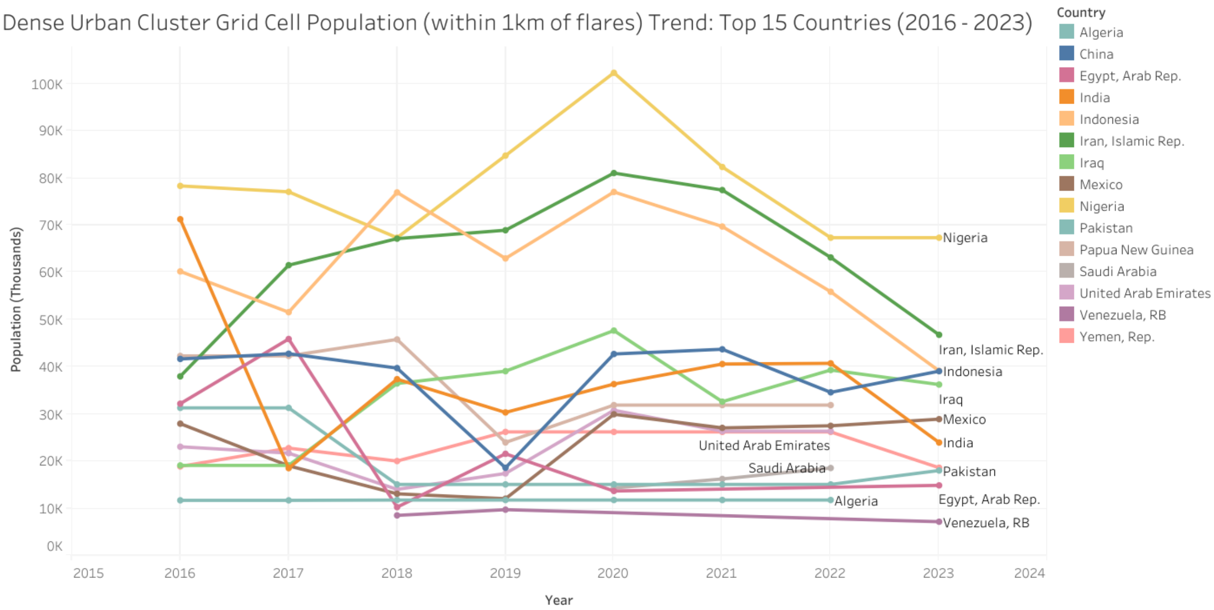

Figure S42: Dense urban population (within 1 km of flares) for the top 15 countries, 2016–2023

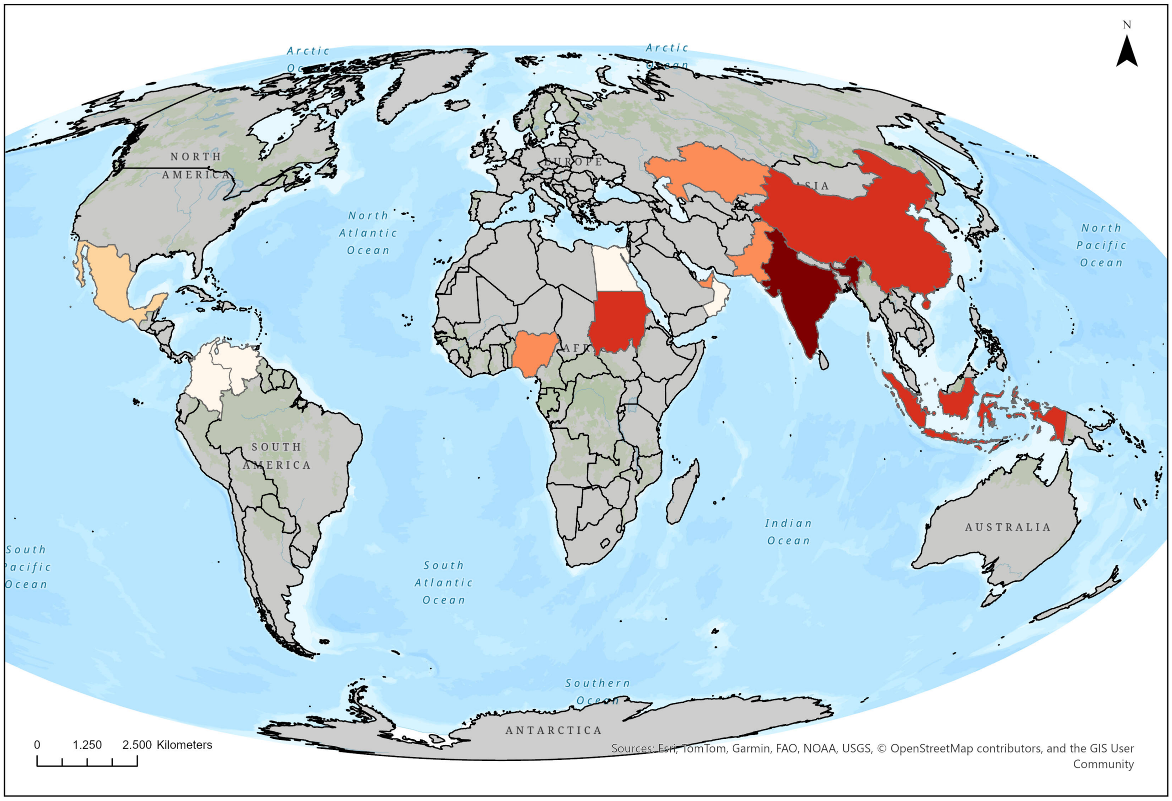

Figure S43: Global maps showing, for each country, the maximum number of people residing in semi-dense urban areas within a 1 km radius of active flaring sites for the year 2023

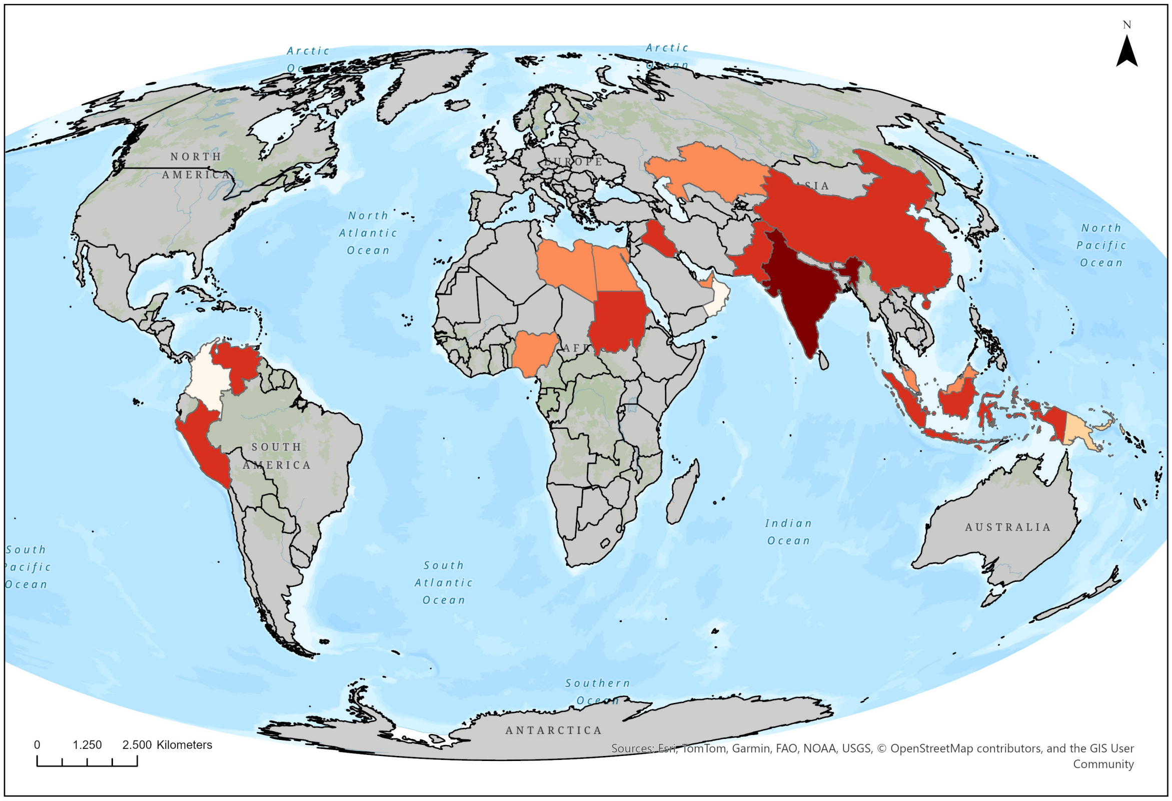

Figure S44: Global maps showing, for each country, the maximum number of people residing in semi-dense urban areas within a 1 km radius of active flaring sites for the year 2022

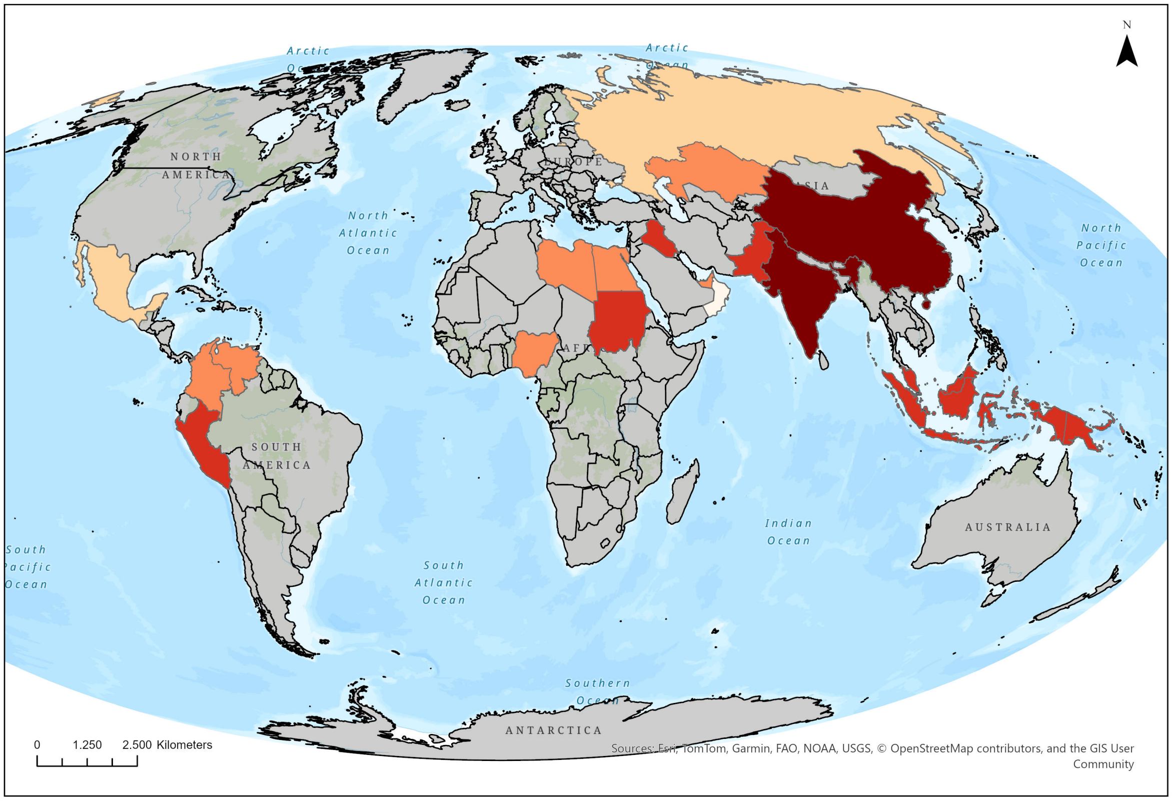

Figure S45: Global maps showing, for each country, the maximum number of people residing in semi-dense urban areas within a 1 km radius of active flaring sites for the year 2021

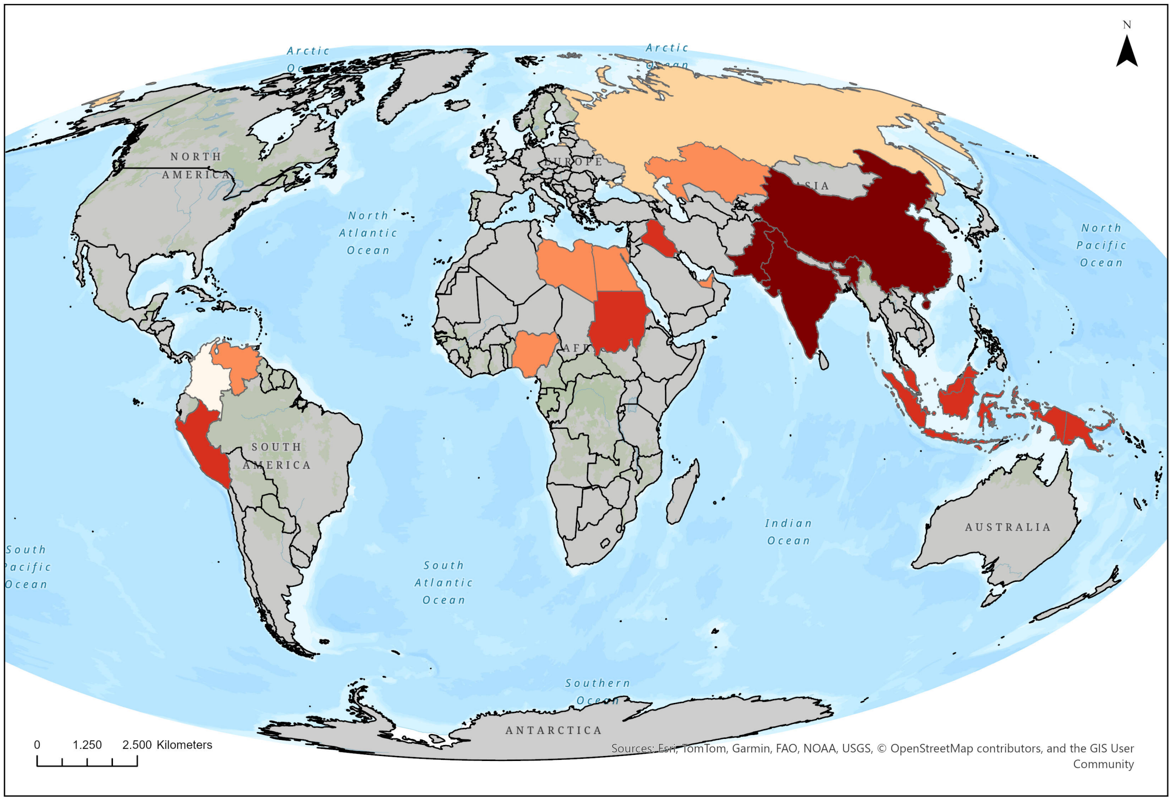

Figure S46: Global maps showing, for each country, the maximum number of people residing in semi-dense urban areas within a 1 km radius of active flaring sites for the year 2020

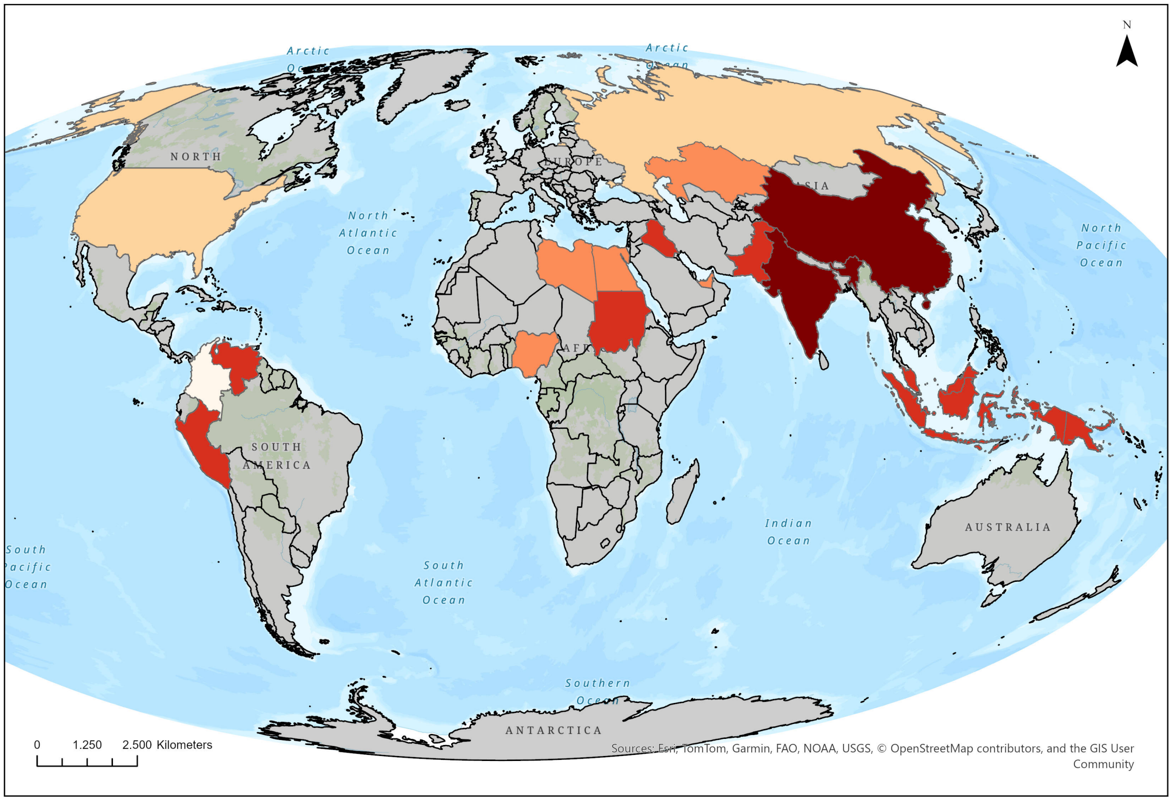

Figure S47: Global maps showing, for each country, the maximum number of people residing in semi-dense urban areas within a 1 km radius of active flaring sites for the year 2019

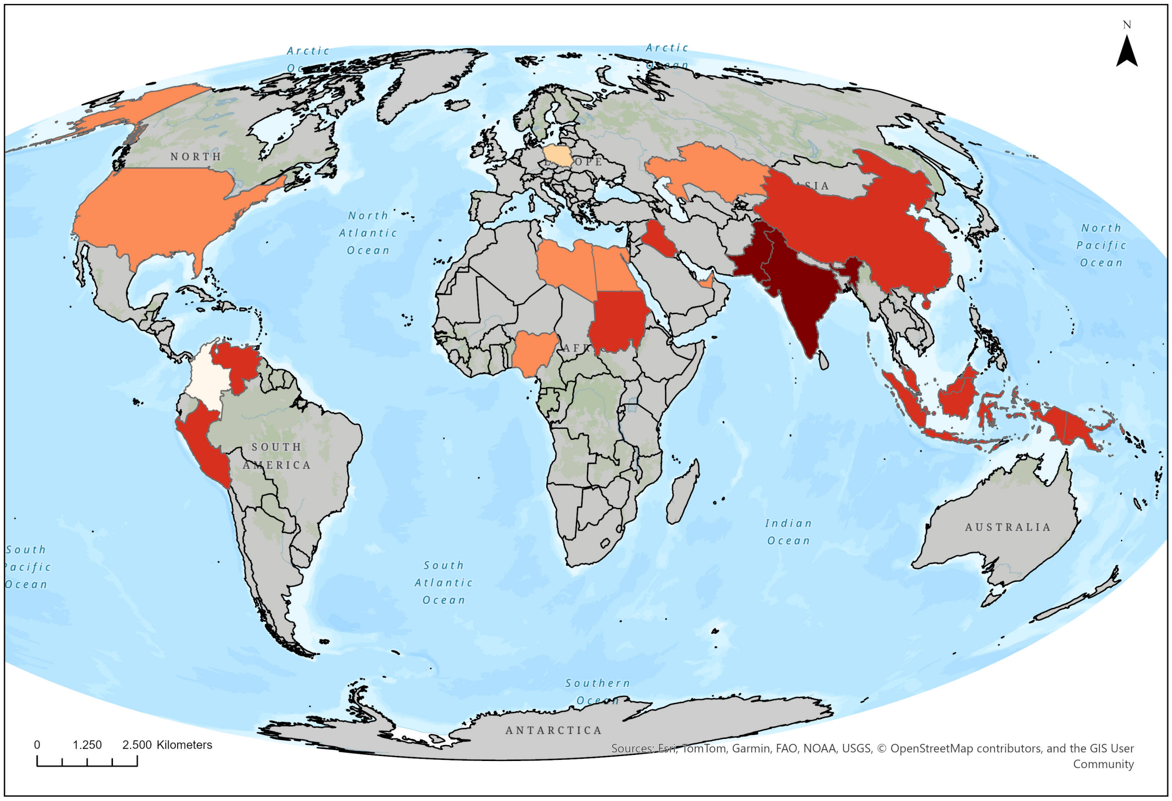

Figure S48: Global maps showing, for each country, the maximum number of people residing in semi-dense urban areas within a 1 km radius of active flaring sites for the year 2018

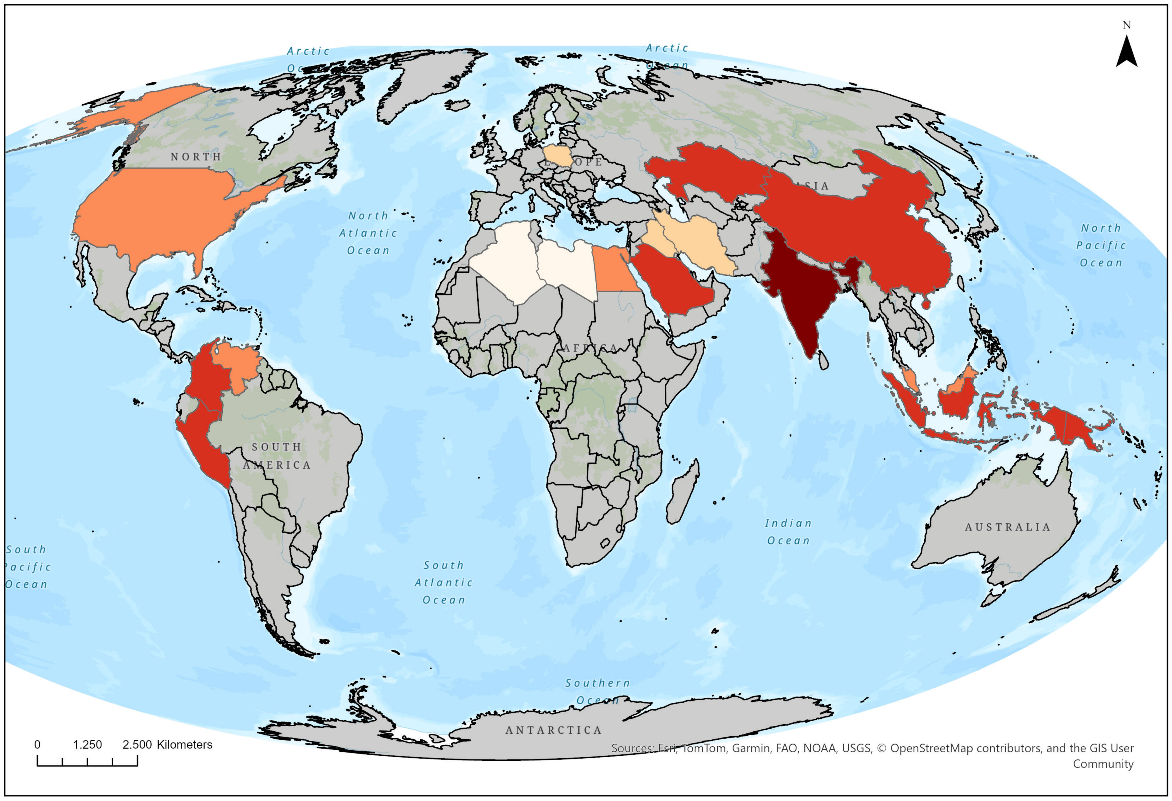

Figure S49: Global maps showing, for each country, the maximum number of people residing in semi-dense urban areas within a 1 km radius of active flaring sites for the year 2017

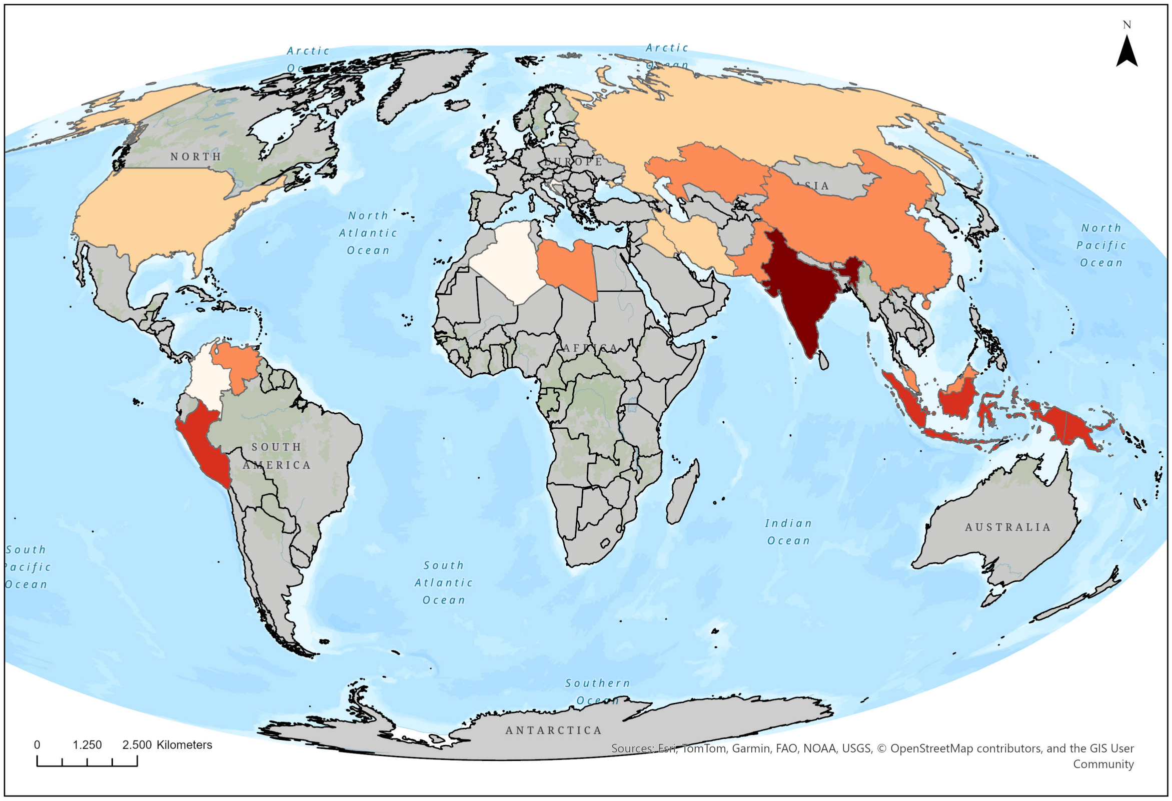

Figure S50: Global maps showing, for each country, the maximum number of people residing in semi-dense urban areas within a 1 km radius of active flaring sites for the year 2016

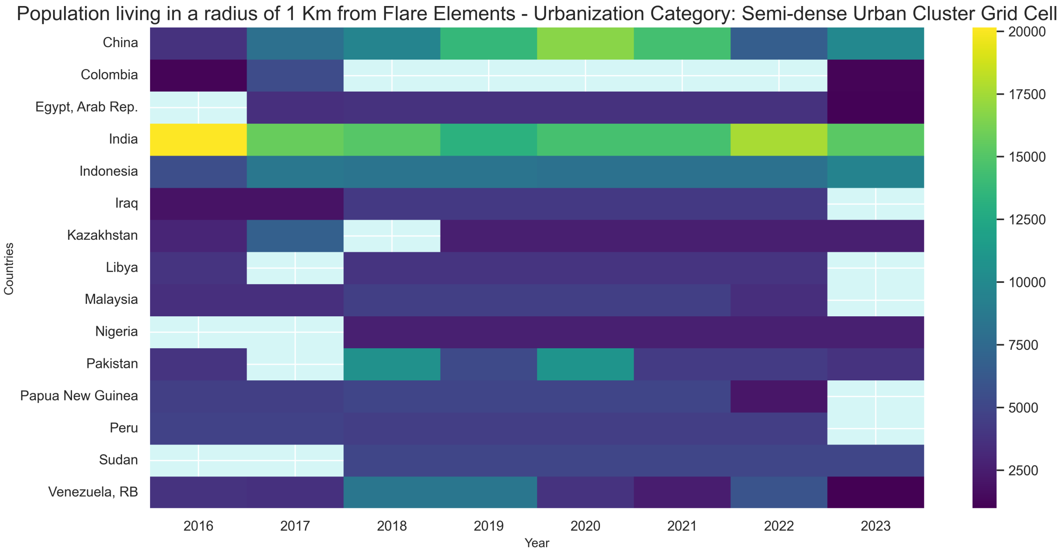

Figure S51: Heatmap of semi-dense urban population (within 1 km of flares) for the top 15 countries, 2016–2023
