# Supplementary material for: Global Mapping of Population Exposure to Upstream Gas Flaring Using Integrated VIIRS Nightfire and GHSL Data, 2016–2023, with Projections to 2030
Source: Toxics. 2025 Dec 5;13(12):1053. doi: 10.3390/toxics13121053 (PMC12737142; doi:10.3390/toxics13121053)
Supplement: Supplementary file 1 [file toxics-13-01053-s001.zip › Supplementary Figures S52-S72.pdf]

Supplementary Materials for

Global Mapping of Population Exposure to Upstream Gas Flaring Using Integrated VIIRS Nightfire and GHSL Data, 2016–2023, with Projections to 2030

This PDF includes:

Supplementary Figures S52–S72

Supplementary Figures

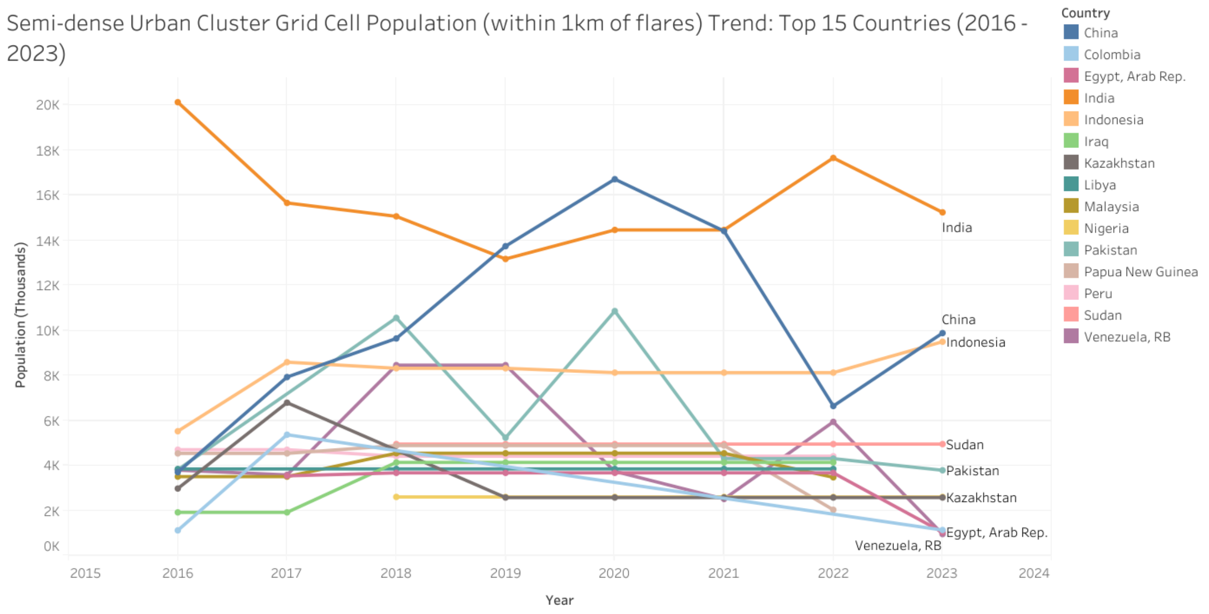

Figure S52: Total semi-dense urban population (within 1 km of flares) for the top 15 countries, 2016–2023

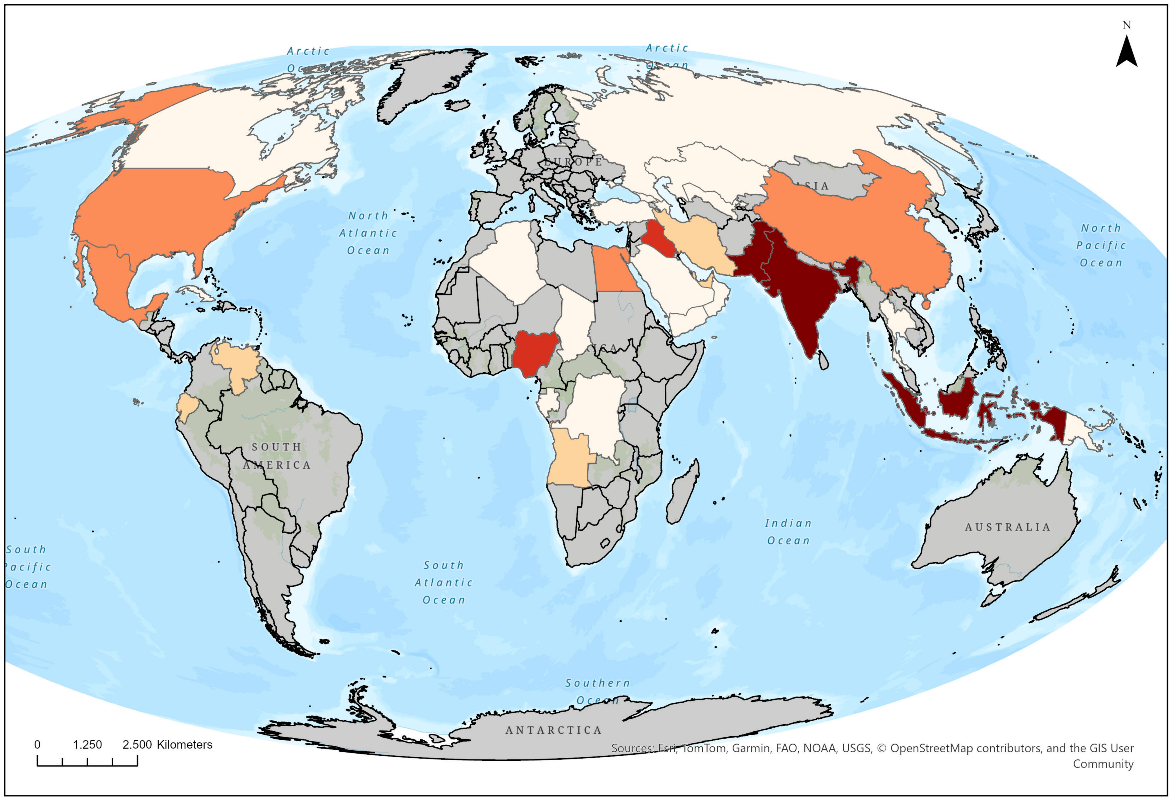

Figure S53: Global maps showing, for each country, the maximum number of people residing in suburban/peri-urban areas within a 1 km radius of active flaring sites for the year 2023

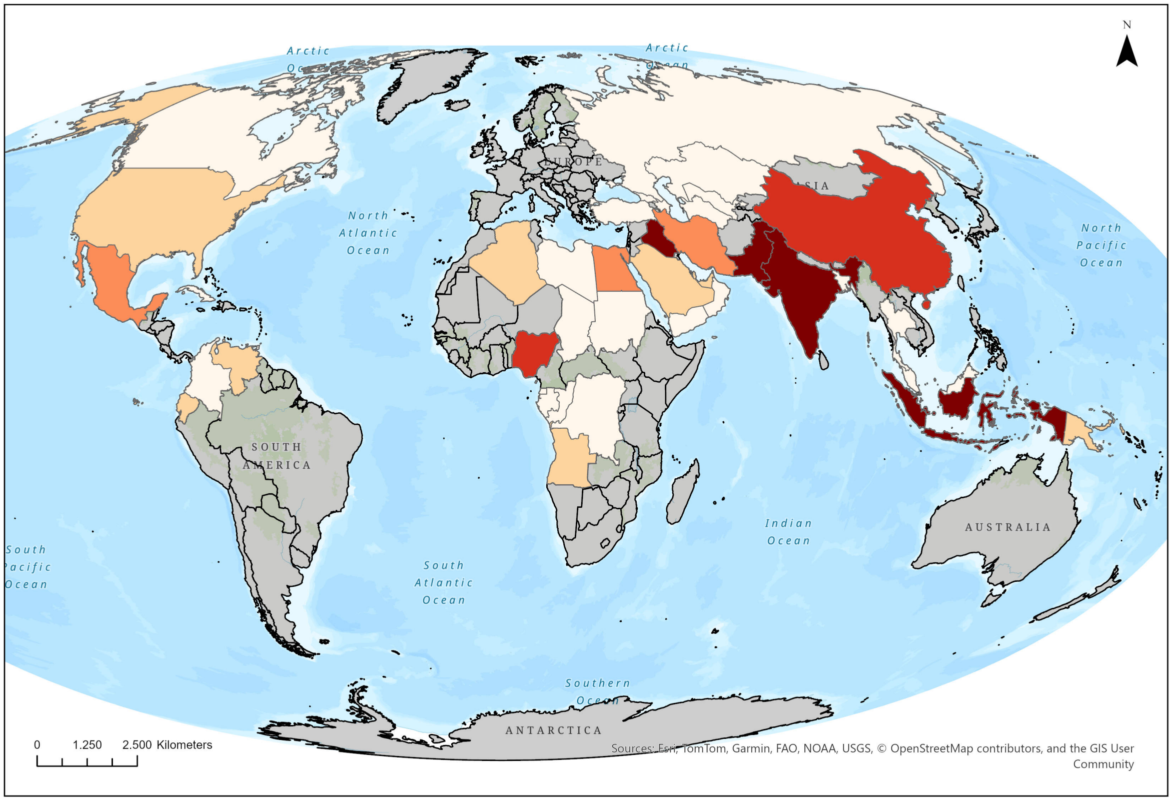

Figure S54: Global maps showing, for each country, the maximum number of people residing in suburban/peri-urban areas within a 1 km radius of active flaring sites for the year 2022

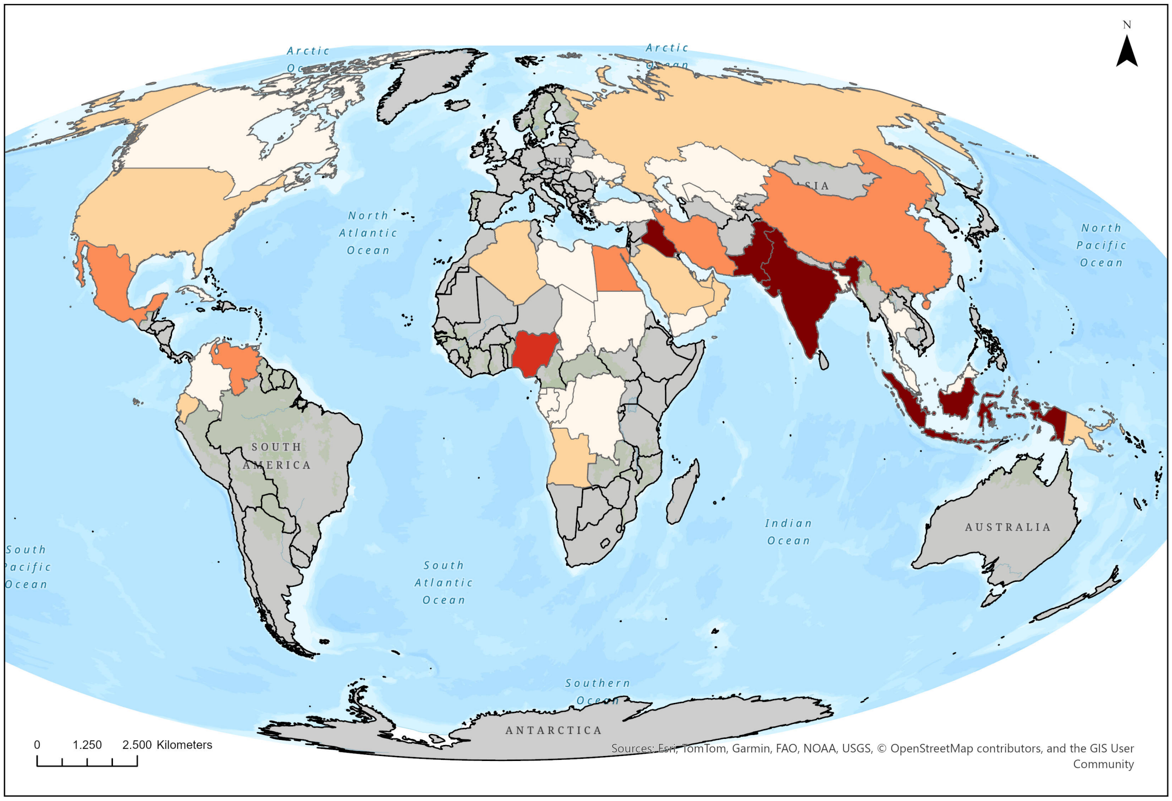

Figure S55: Global maps showing, for each country, the maximum number of people residing in suburban/peri-urban areas within a 1 km radius of active flaring sites for the year 2021

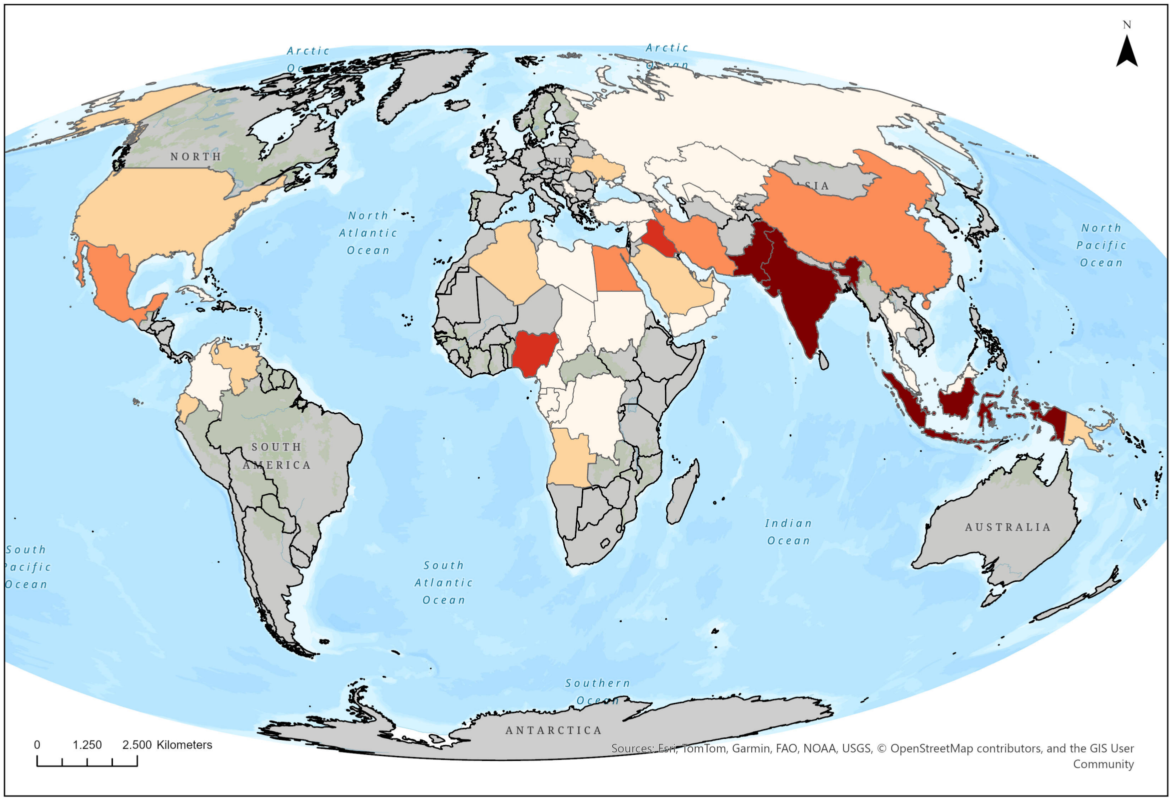

Figure S56: Global maps showing, for each country, the maximum number of people residing in suburban/peri-urban areas within a 1 km radius of active flaring sites for the year 2020

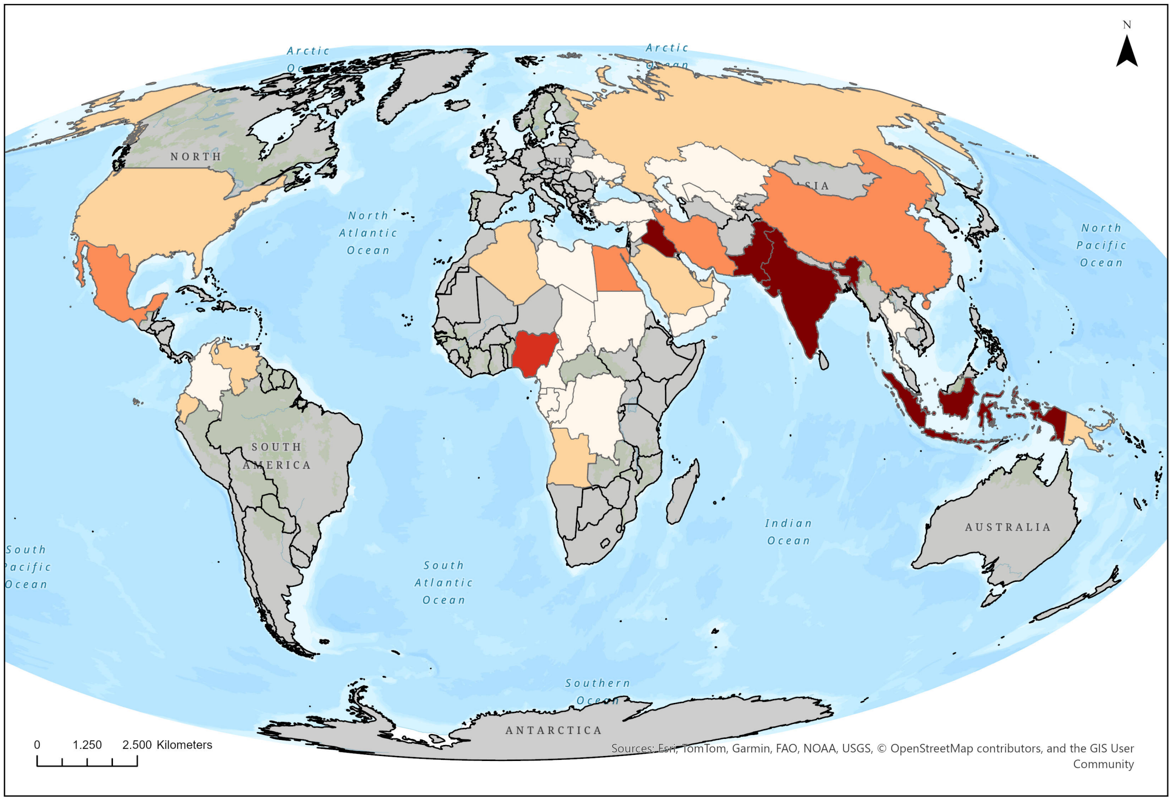

Figure S57: Global maps showing, for each country, the maximum number of people residing in suburban/peri-urban areas within a 1 km radius of active flaring sites for the year 2019

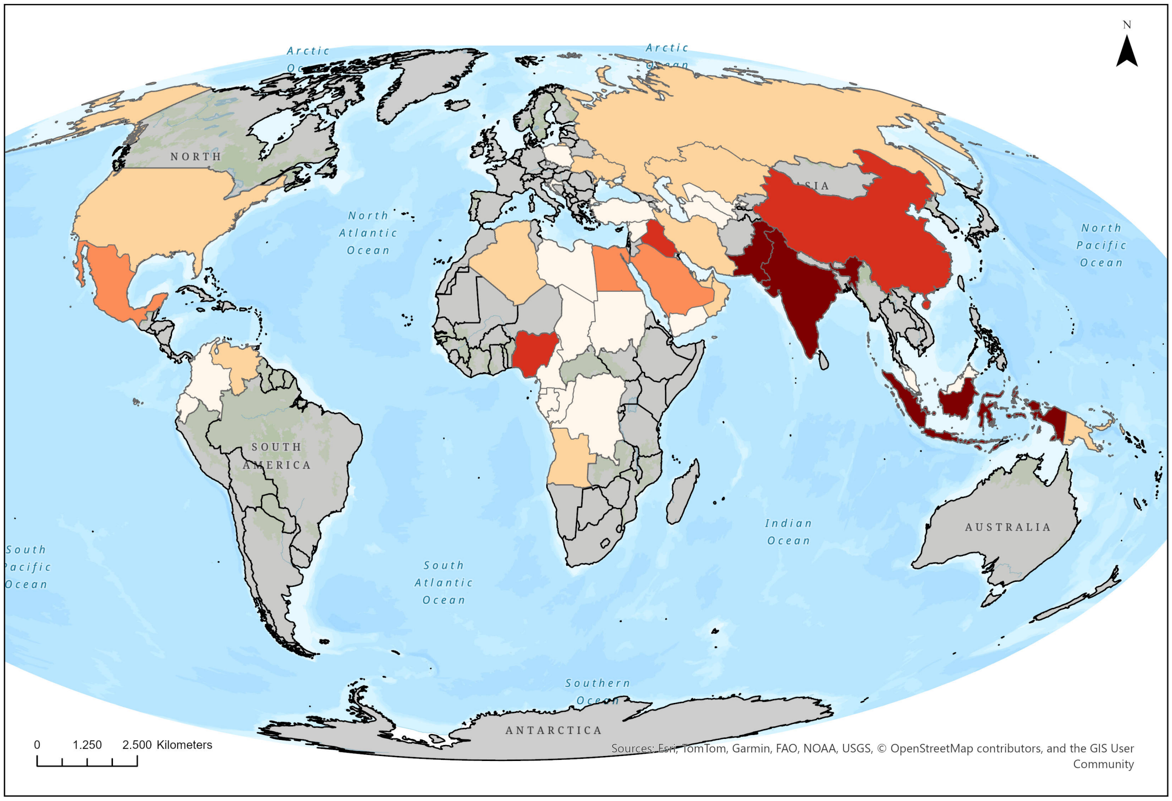

Figure S58: Global maps showing, for each country, the maximum number of people residing in suburban/peri-urban areas within a 1 km radius of active flaring sites for the year 2018

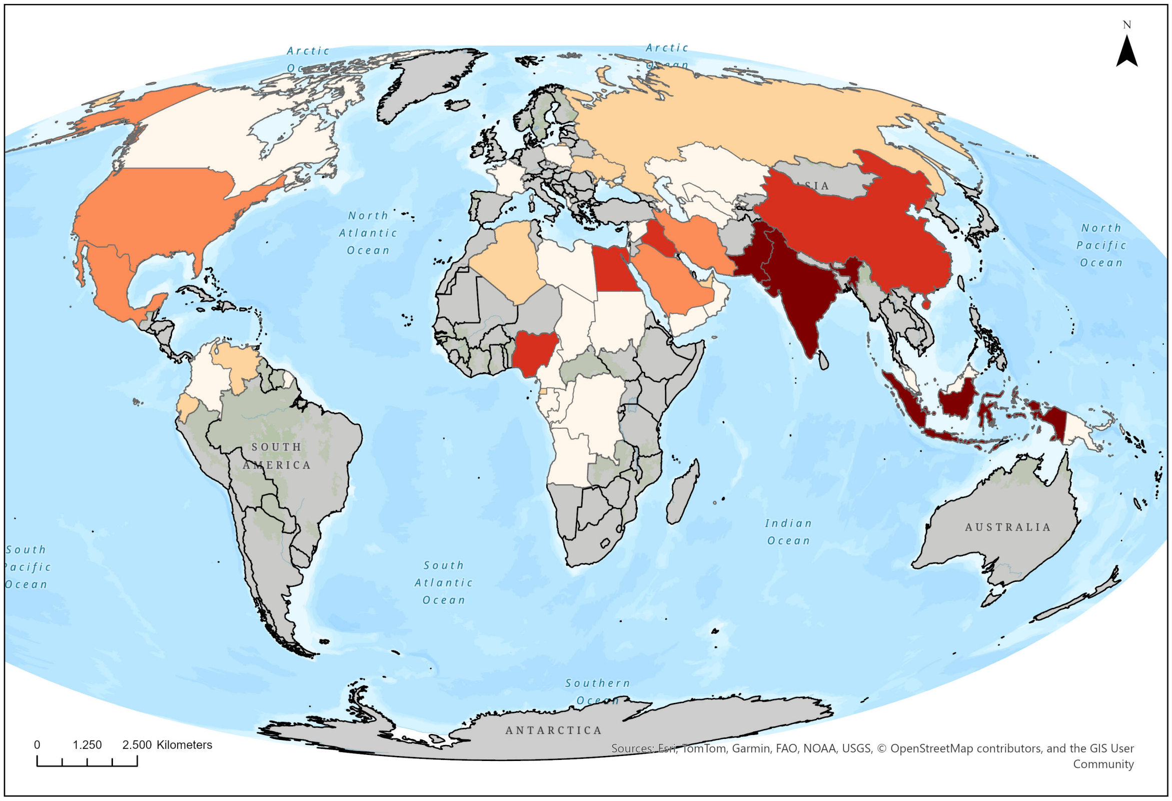

Figure S59: Global maps showing, for each country, the maximum number of people residing in suburban/peri-urban areas within a 1 km radius of active flaring sites for the year 2017

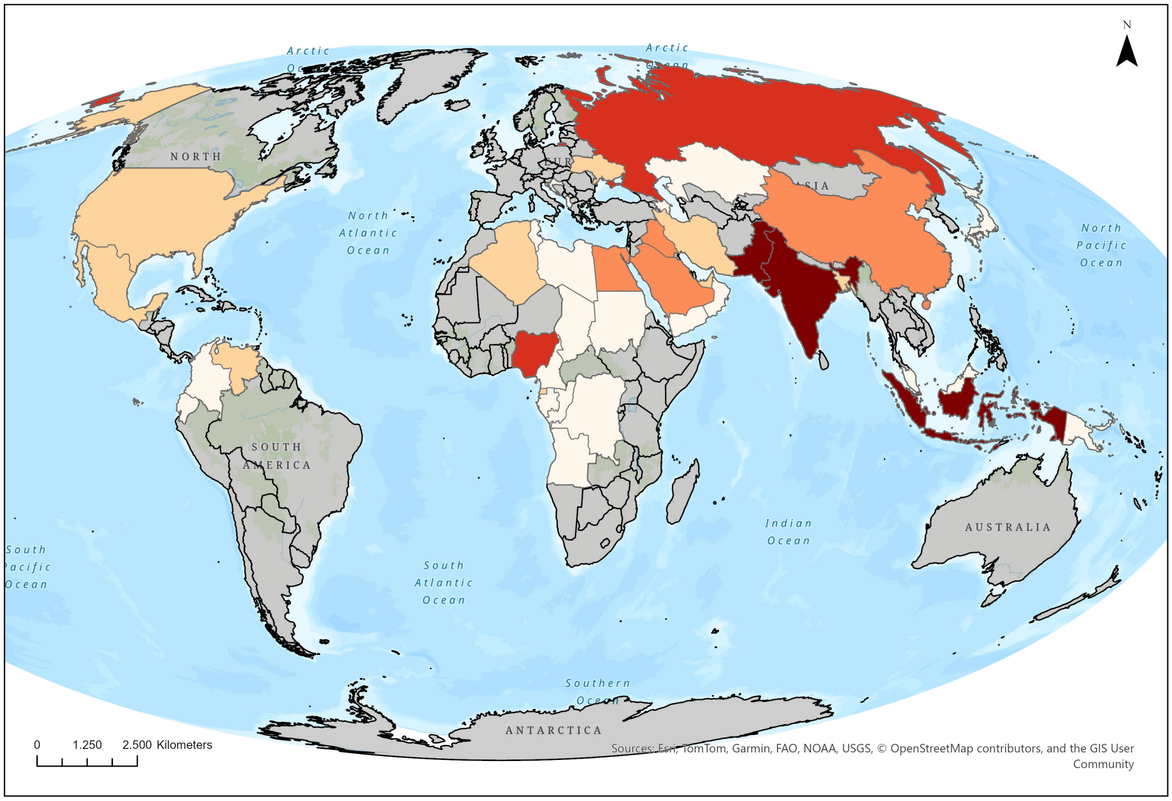

Figure S60: Global maps showing, for each country, the maximum number of people residing in suburban/peri-urban areas within a 1 km radius of active flaring sites for the year 2016

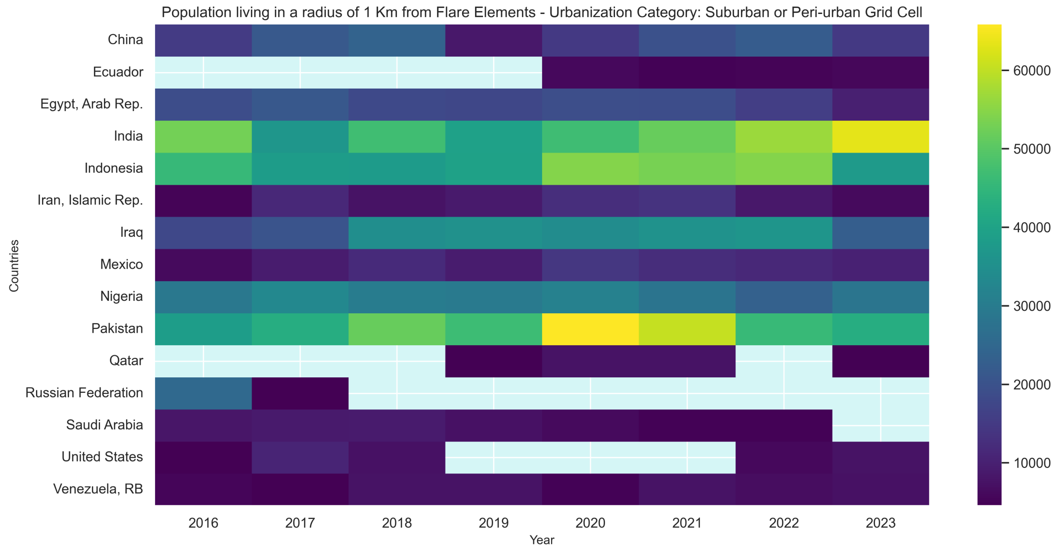

Figure S61: Heatmap of suburban population (within 1 km of flares) for the top 15 countries, 2016–2023

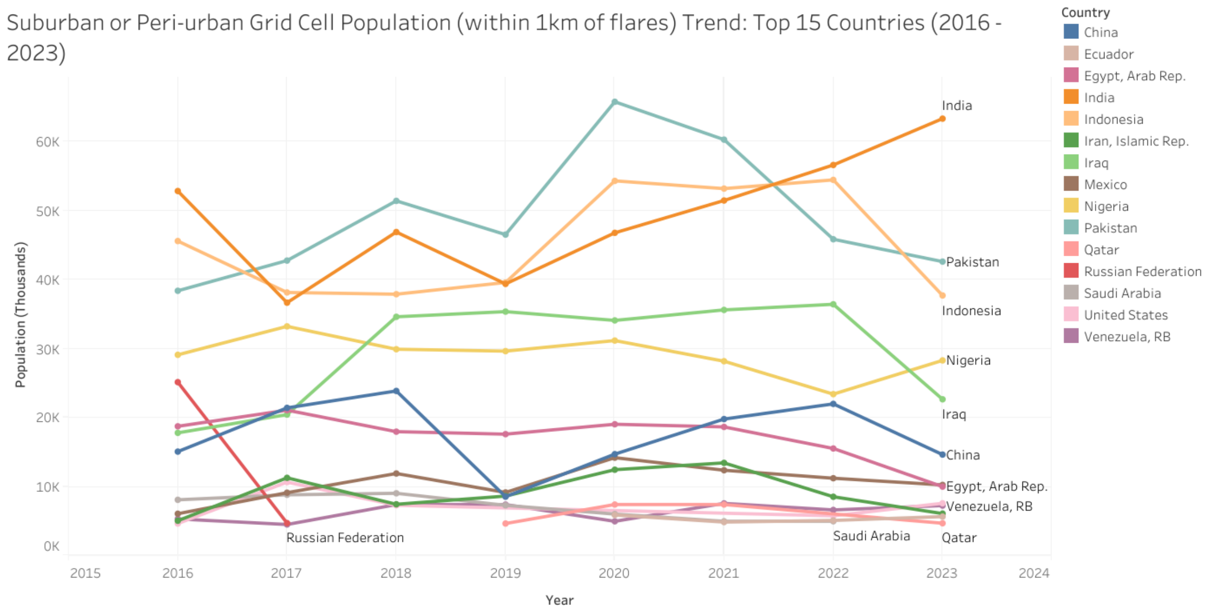

Figure S62: Suburban population (within 1 km of flares) for the top 15 countries, 2016–2023

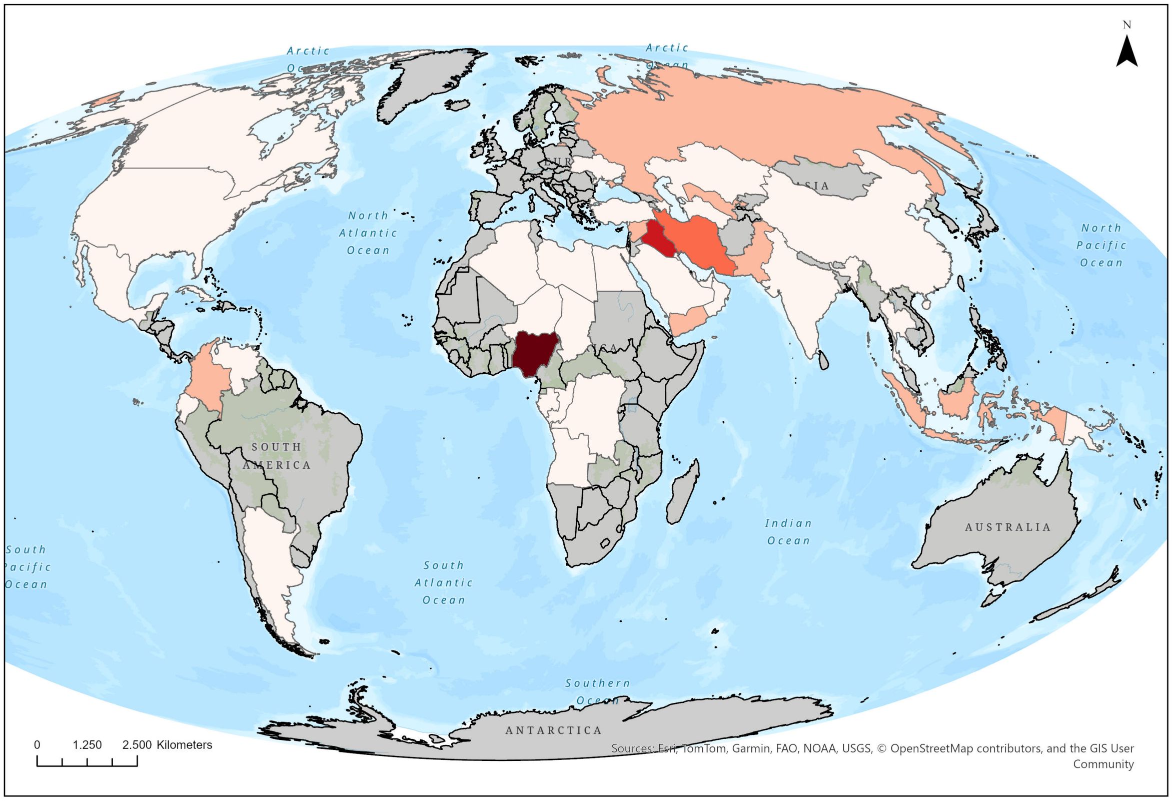

Figure S63: Global maps showing, for each country, the maximum number of people residing in **rural areas** within a 1 km radius of active flaring sites for the year 2023

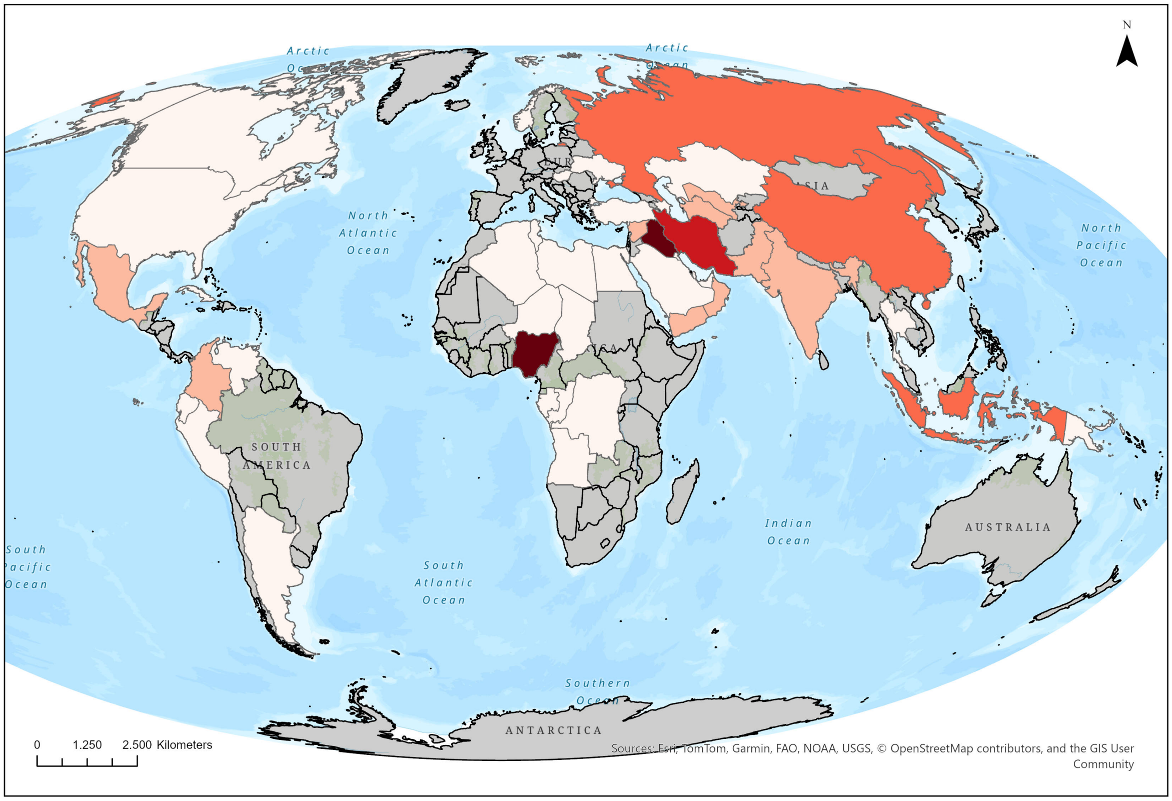

Figure S64: Global maps showing, for each country, the maximum number of people residing in **rural areas** within a 1 km radius of active flaring sites for the year 2022

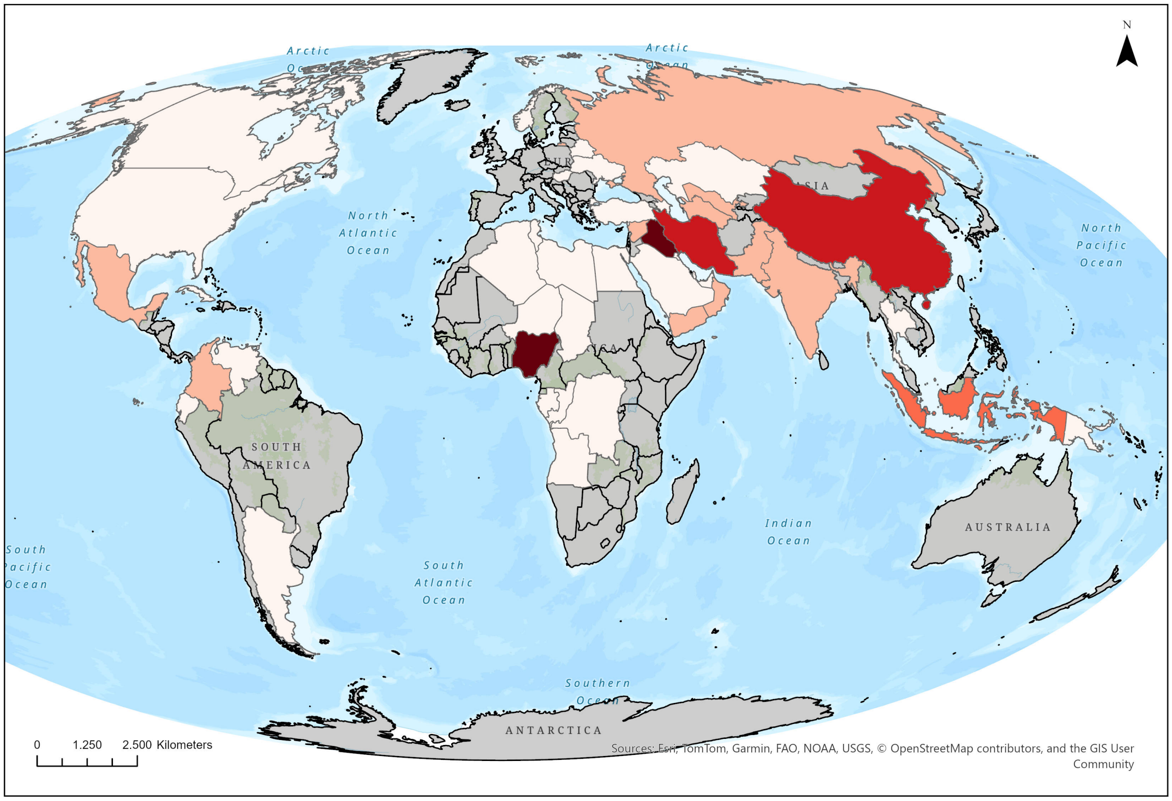

Figure S65: Global maps showing, for each country, the maximum number of people residing in **rural areas** within a 1 km radius of active flaring sites for the year 2021

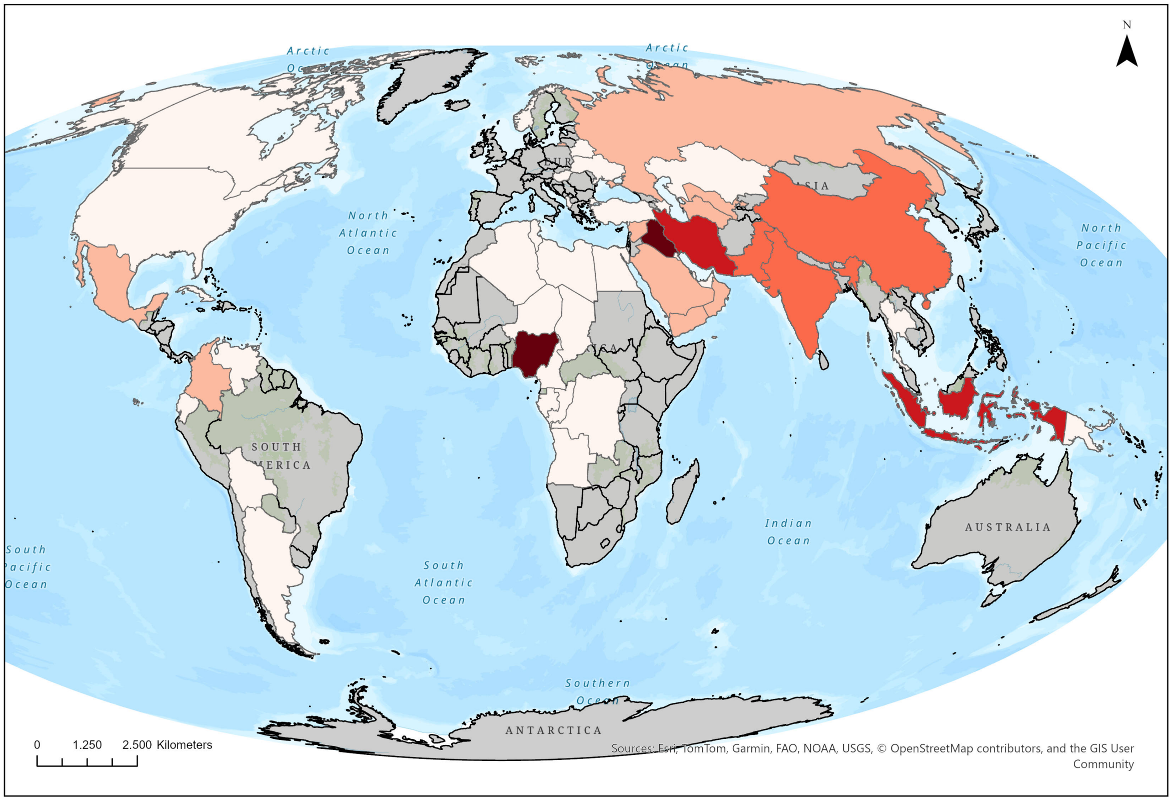

Figure S66: Global maps showing, for each country, the maximum number of people residing in **rural areas** within a 1 km radius of active flaring sites for the year 2020

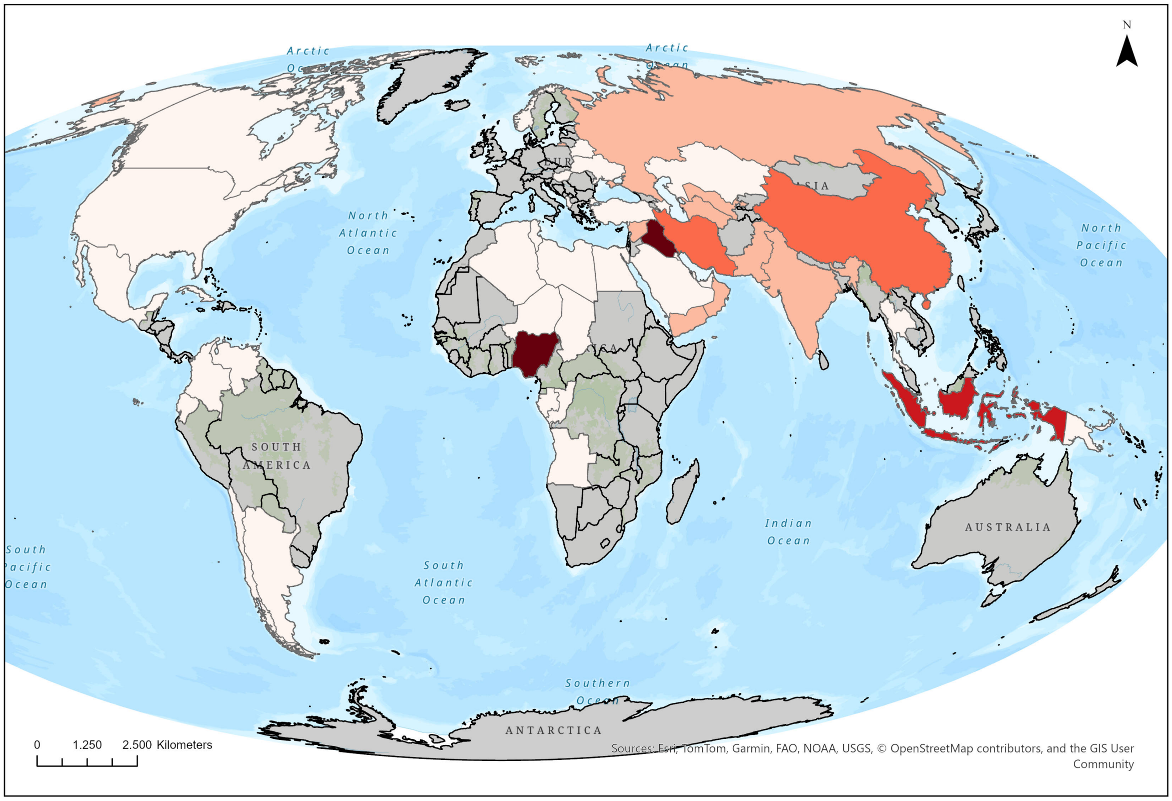

Figure S67: Global maps showing, for each country, the maximum number of people residing in **rural areas** within a 1 km radius of active flaring sites for the year 2019

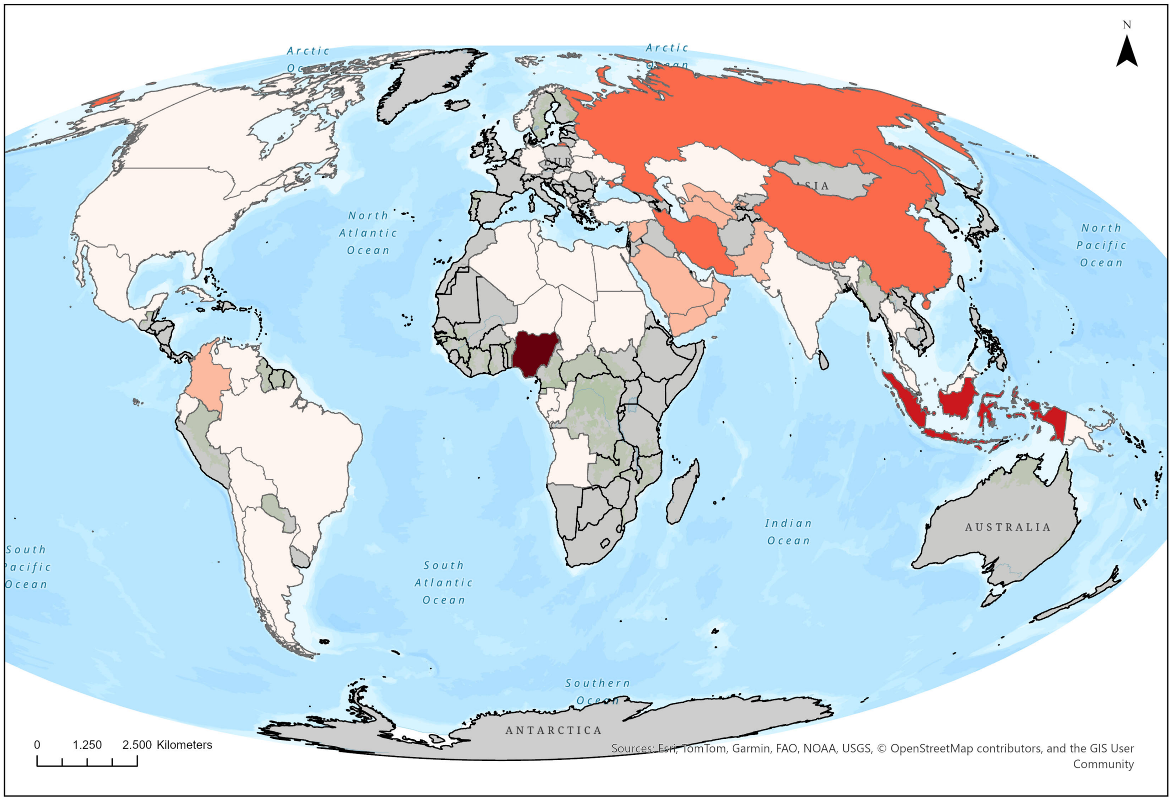

Figure S68: Global maps showing, for each country, the maximum number of people residing in **rural areas** within a 1 km radius of active flaring sites for the year 2018

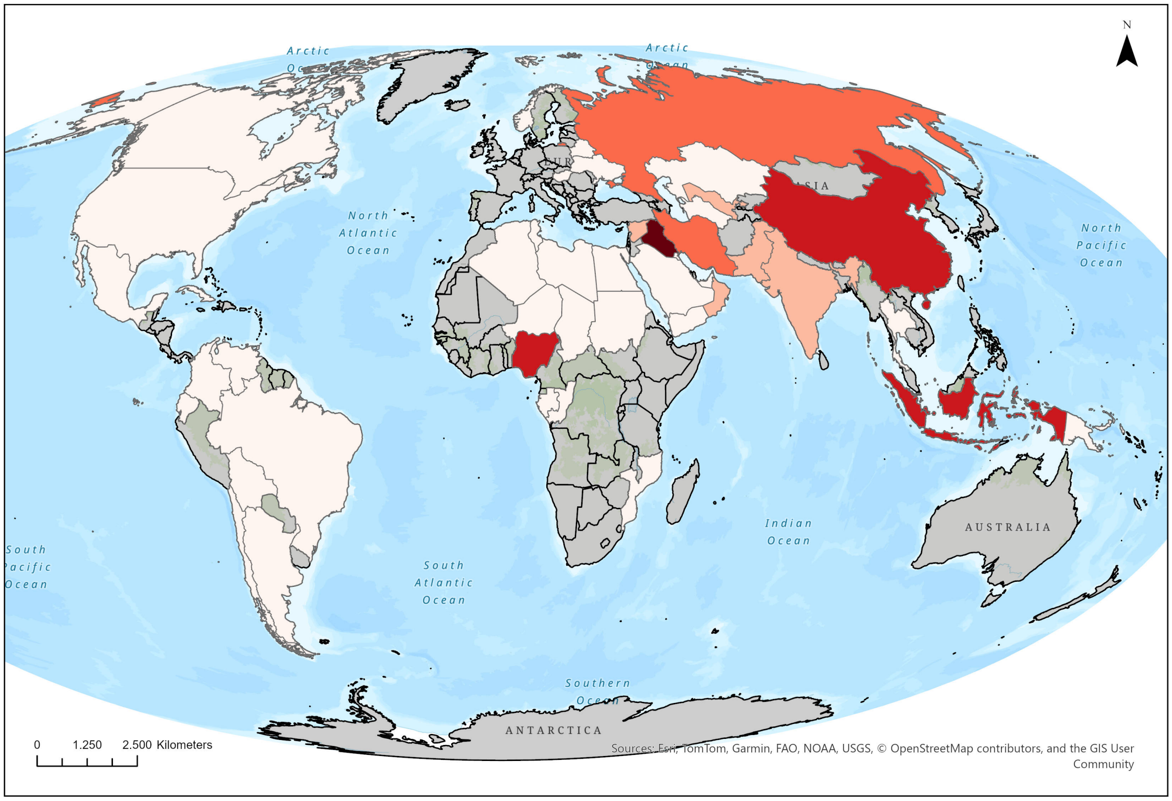

Figure S69: Global maps showing, for each country, the maximum number of people residing in **rural areas** within a 1 km radius of active flaring sites for the year 2017

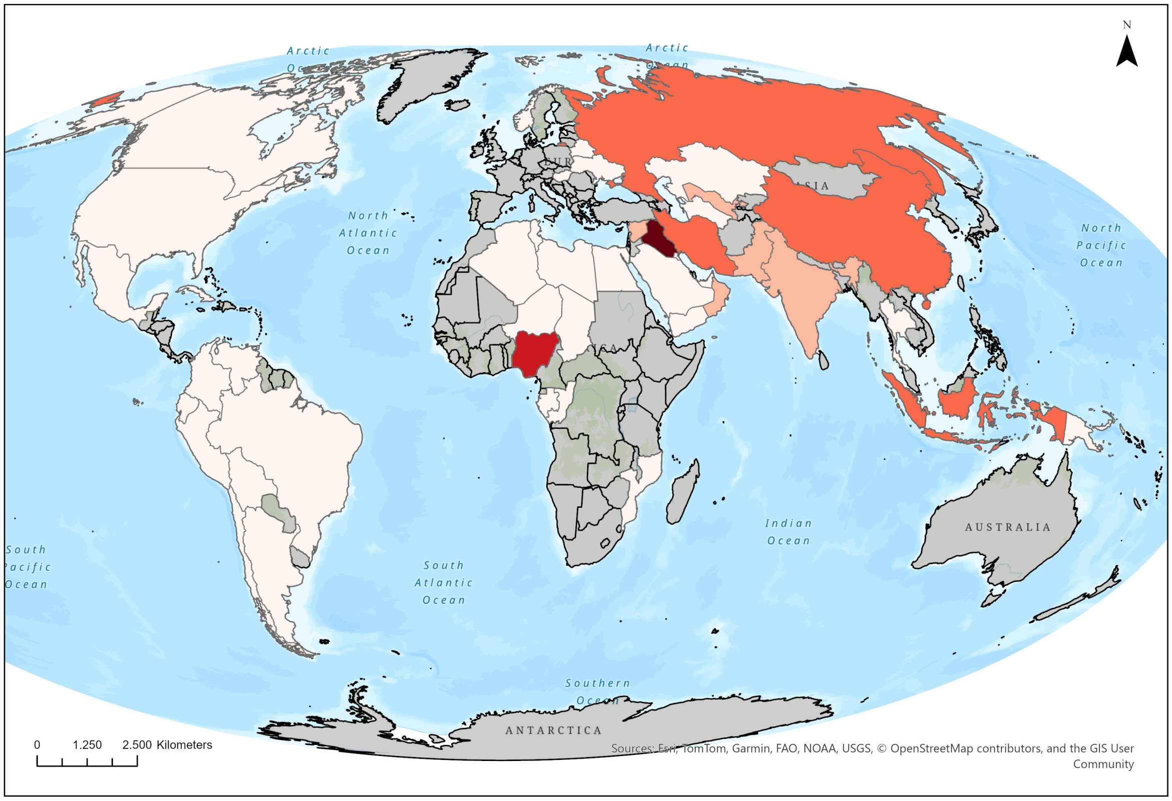

Figure S70: Global maps showing, for each country, the maximum number of people residing in **rural areas** within a 1 km radius of active flaring sites for the year 2016

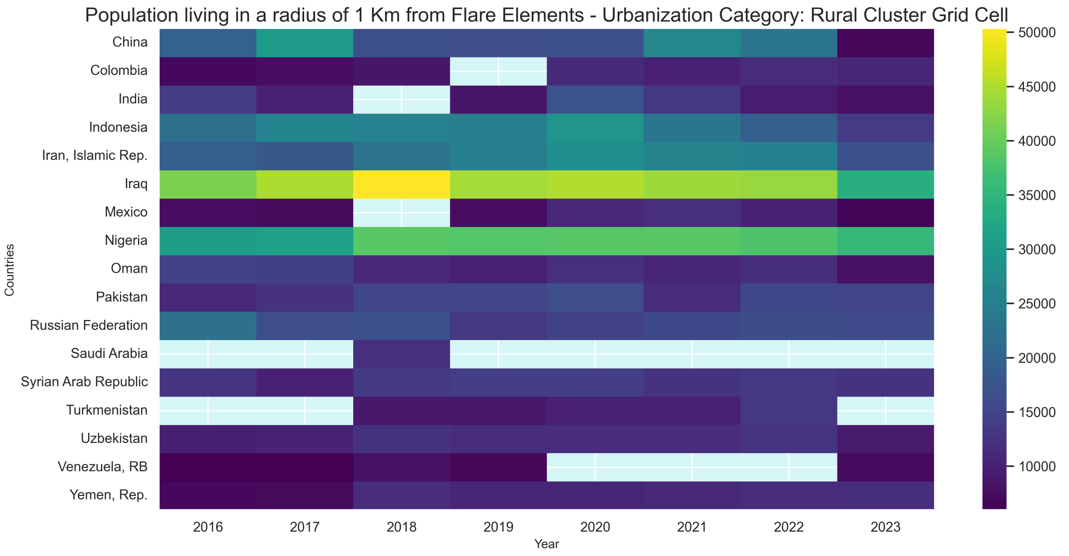

Figure S71: Heatmap of rural population (within 1 km of flares) for the top 15 countries, 2016–2023

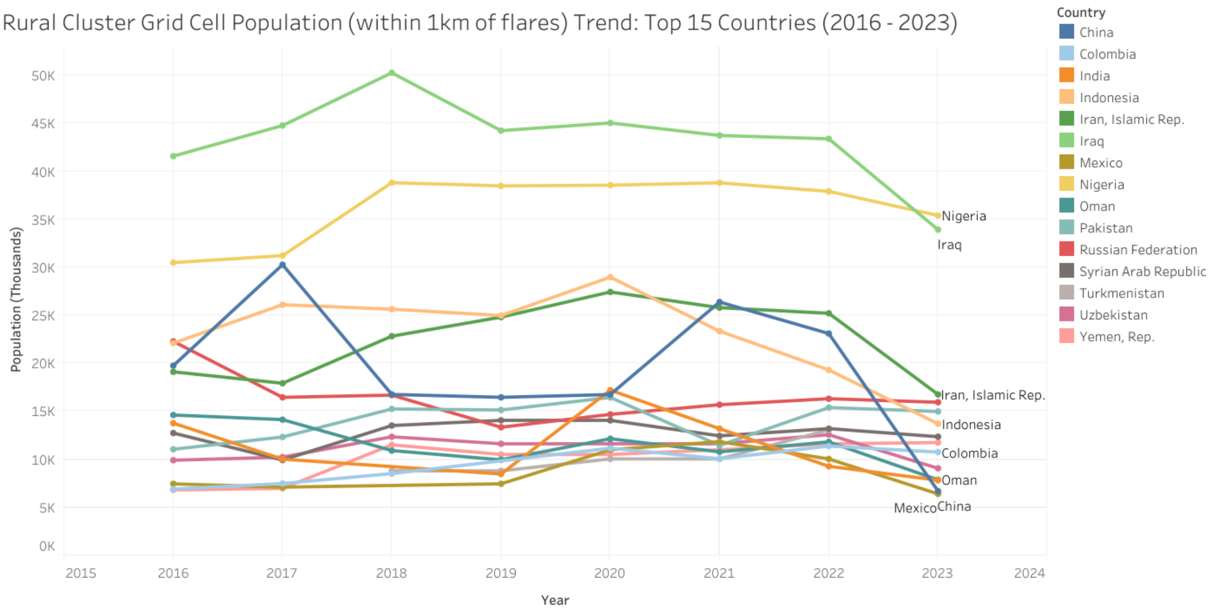

Figure S72: Total rural population (within 1 km of flares) for the top 15 countries, 2016–2023
